# Stroke Mechanism and Severity After Left Atrial Appendage Occlusion: Insights From the LAAOS III Randomized Clinical Trial

**DOI:** 10.1001/jamaneurol.2025.4478

**Published:** 2025-11-17

**Authors:** Aristeidis H. Katsanos, Richard P. Whitlock, Emilie P. Belley-Côté, Katheryn Brady, Angela Wang, Abhilekh Srivastava, Gregory Jacquin, Viktor Weiss, Ondřej Volný, Martin Sramek, Andre Peeters, João Pedro Marto, Pawel Wrona, Anthoula Tsolaki, Linxin Li, Antonia Nucera, Robert Mikulik, Kanjana Perera, Luciana Catanese, Ashkan Shoamanesh, Mukul Sharma

**Affiliations:** 1 Population Health Research Institute, and Hamilton Health Sciences, Hamilton, Ontario, Canada; 2 Division of Neurology, Department of Medicine, McMaster University, Hamilton, Ontario, Canada; 3 Division of Cardiac Surgery, Department of Surgery, McMaster University, Hamilton, Ontario, Canada; 4 Divisions of Cardiology and Critical Care, Department of Medicine, McMaster University, Hamilton, Ontario, Canada; 5 Université de Montréal, Faculté de Médecine, Département de Neurosciences, Montréal Québec, Canada; 6 Department of Neurology, St. Anne’s University Hospital, Masaryk University, Brno, Czech Republic & Department of Neurology, Tomas Bata Hospital, Zlin, Czech Republic; 7 University of Ostrava Faculty of Medicine, Department of Clinical Neurosciences, Ostrava, Czech Republic; 8 Department of Neurology, Military University Hospital Prague, Prague, Czech Republic; 9 Department of Neurology, Cliniques Universitaires Saint Luc, UCLouvain, Brussels, Belgium; 10 Department of Neurology, Centro Hospitalar Lisboa Ocidental, Lisbon, Portugal; 11 Department of Neurology, Jagiellonian University Medical College, Krakow, Poland; 12 First Department of Neurology, Medical School, Faculty of Health Sciences, Aristotle University of Thessaloniki, Macedonia, Greece; 13 Wolfson Centre for Prevention of Stroke and Dementia, Nuffield Department of Clinical Neurosciences, Oxford University, United Kingdom; 14 Neurovascular Treatment Unit, Spaziani Hospital, Frosinone, Italy

## Abstract

**Importance:**

In the Left Atrial Appendage Occlusion Study III (LAAOS III), surgical occlusion of the LAA during cardiac surgery for patients with known history of atrial fibrillation (AF) substantially reduced the risk of stroke.

**Objective:**

To assess the impact of LAAO on ischemic stroke subtype and outcome.

**Design, Setting, and Participants:**

This was a post hoc exploratory analysis of the LAAOS III randomized clinical trial. Data were adjudicated from June 28, 2023, to November 29, 2023, and the main analyses took place from December 18, 2023, to April 29, 2024. The LAAOS III trial recruited participants from 105 centers in 27 countries between July 2012 and October 2018. Patients with AF and a CHA_2_DS_2_-VASc score of at least 2 undergoing cardiac surgery for other indications were included in the analysis.

**Interventions:**

Surgical LAAO plus standard care vs standard care alone.

**Main Outcomes and Measures:**

For strokes occurring during the trial, the functional outcome as measured by the modified Rankin Scale (mRS) score at day 7 or discharge, mortality, the presence of cortical infarcts, and the occurrence of infarcts of presumed cardioembolic origin were examined.

**Results:**

Of 4811 participants in the LAAOS III trial followed up for 3.8 years, 273 had a first ischemic stroke. The mean (SD) age of participants at the time of the first ischemic stroke was 75 (7) years, 104 were female (38%), and 169 were male (62%). Participants allocated to receive LAAO had reduced (common odds ratio [OR], 0.80; 95% CI, 0.65-0.99) mRS scores at 7 days or discharge and a lower risk for mortality at 30 days (16.5% vs 20.1%; hazard ratio [HR], 0.55; 95% CI, 0.31-0.97) after a stroke event. Participants allocated to LAAO had fewer cortical infarcts on neuroimaging (46.2% vs 61.3%; difference in proportions: −15.2%; 95% CI, −26.7% to −3.7%), as well as a lower proportion of ischemic strokes of presumed cardioembolic etiology when compared with ischemic strokes in the no-LAAO group (42.9% vs 57.9%; difference in proportions: −15.1%; 95% CI, −26.5% to −3.7%).

**Conclusions and Relevance:**

This study found that LAAO in patients with AF undergoing cardiac surgery was associated with a decreased risk of presumed cardioembolic stroke, reduced disability, and mortality from stroke. These findings underscore the benefit of LAAO for patients with AF undergoing cardiac surgery.

**Trial Registration:**

ClinicalTrials.gov Identifier: NCT01561651

## Key Points

**Question:**

Are ischemic stroke events after surgical occlusion of the left atrial appendage (LAAO) during cardiac surgery for patients with known history of atrial fibrillation (AF) of milder severity and less likely to be cardioembolic compared with ischemic stroke events occurring in individuals with AF and without LAAO?

**Findings:**

This post hoc exploratory analysis of the Left Atrial Appendage Occlusion Study III (LAAOS III) trial, which randomized 4811 participants with a history of atrial fibrillation undergoing cardiac surgery to receive surgical LAAO or no LAAO, found that LAAO reduced the mortality and disability from ischemic stroke and the proportion of cardioembolic strokes.

**Meaning:**

Study findings complement the reduction in stroke occurrence observed in the primary analysis of the LAAOS III trial and further highlight the benefit of LAAO in stroke prevention among patients with AF undergoing cardiac surgery.

## Teaser

This secondary analysis of the Left Atrial Appendage Occlusion Study III (LAAOS III) randomized clinical trial investigates the impact of LAAO on ischemic stroke subtype and outcome.

## Introduction

Atrial fibrillation (AF) elevates the risk of stroke 5-fold and accounts for over 40% of all strokes in individuals older than 80 years.^1,2^ Over the past 3 decades, the incidence of cardioembolic strokes attributed to AF has tripled and is expected to rise further with the aging population.^3^ Strokes caused by AF double the risk of death and disability, when compared with strokes of other etiology, and carry a high risk of recurrence.^4,5^

Oral anticoagulation with vitamin K antagonists has been shown to reduce the risk of stroke by 64% and death by 26% in patients with AF.^6^ Non–vitamin K oral anticoagulants reduced the risk of stroke by an additional 19% and halved the risk of intracranial bleeding when compared with warfarin.^7^ Despite this progress for patients with AF, the long-term risk of stroke occurrence remains considerable.^8^ In the Left Atrial Appendage Occlusion Study III (LAAOS III),^9^ surgical occlusion of the LAA during cardiac surgery for patients with history of AF reduced the risk of stroke or systemic embolism by 33%.^9^

We sought to characterize stroke topography, presumed subtype, disability, and mortality to assess the impact of LAAO on ischemic stroke mechanism and outcome. Our hypotheses for this post hoc exploratory analysis were that ischemic stroke events after LAAO would be of lower severity and less likely to have cortical involvement and a presumed cardioembolic subtype than in participants without LAAO. We also hypothesized that LAAO would not be associated with a higher incidence of perioperative strokes (during surgery or within 30 days after surgery).

## Methods

The protocol of the LAAOS III trial was approved by health authorities and institutional review boards in all participating countries, and written informed consent was obtained from all participants.^9^ Ethics approval was not required for this exploratory analysis because these analyses use preexisting, anonymized data that were already collected under the approved study protocol. The trial protocol and statistical analysis plan are available in Supplement 1 and Supplement 2, respectively. This study followed the Consolidated Standards of Reporting Trials (CONSORT) reporting guidelines.

The LAAOS III trial was a large, randomized clinical trial that evaluated the efficacy of LAAO during cardiac surgery performed for other reasons in reducing the risk of stroke in patients with AF. The primary results have been previously published.^9^ The trial enrolled 4811 participants with AF and a CHA_2_DS_2_-VASc score (calculated using point values for congestive heart failure, hypertension, years of age, diabetes, stroke or transient ischemic attack, vascular disease, and sex category) of at least 2 who were undergoing cardiac surgery for other indications. Participants were randomized to undergo surgical LAAO or to receive standard care without occlusion.^9^ Participants were expected to receive guideline-directed stroke prevention and other usual-care measures, including anticoagulation. Participants, treating teams, and research teams were blinded to the treatment allocation. Treatment allocation was known only to the treating surgeon and not entered into the medical record. The primary outcome was the occurrence of ischemic stroke or systemic embolism.^9^ Participant race and ethnicity information was not available in the dataset.

Stroke neurologists, blinded to treatment allocation, reviewed the imaging and clinical reports of all ischemic strokes in the LAAOS III trial. The adjudication process followed the principles of similar previous analyses of stroke outcomes in clinical trials.^10,11^ Adjudicators were provided with clinical and imaging reports of strokes that occurred during the trial and asked to classify the localization (cortical vs subcortical) and vascular territory (multiple vs single) of infarcts. Classification of cortical vs subcortical relied on imaging characteristics and clinical features. Ischemic strokes involving or limited to the cerebral cortex on imaging (computed tomography [CT] or magnetic resonance imaging [MRI]) or those having cortical signs and symptoms (aphasia or neglect) were categorized as cortical. Ischemic strokes with infarcts in more than 1 vascular territory on CT or MRI were classified as multiple. Adjudicators were also asked to subtype ischemic strokes based on the suspected etiology and according to a predefined algorithm that was based on modified Trial of Org 10172 in Acute Stroke Treatment (TOAST) criteria^12^ outlined in eFigure 1 in Supplement 3. Because all participants in the LAAOS III trial had underlying AF, a stroke was considered to be cardioembolic if multiple acute infarcts in different vascular territories were present or if the acute infarct met the following characteristics (modified TOAST criteria): (1) cortical infarct or subcortical infarct measuring more than 1.5 cm on CT or MRI; (2) absence of greater than 50% stenosis in a vessel supplying the territory of the infarct on carotid Doppler ultrasound, CT angiography, or MR angiography; and (3) no other specific stroke etiology identified on imaging or patient records.

Outcomes for this analysis included the functional status at day 7 or discharge, measured with the modified Rankin Scale (mRS), and stroke-related mortality at 30 days after first ischemic stroke. We also estimated the proportion of cortical infarcts, multiple infarcts, or infarcts of presumed cardioembolic origin in the first and recurrent ischemic stroke events.

### Statistical Analysis

For the primary analysis, we used the intention-to-treat (ITT) population, categorizing participants into LAAO vs no-LAAO groups as per their original treatment allocation. Baseline characteristics for participants at the time of their first index stroke are presented with absolute numbers and corresponding percentages, whereas continuous data are reported as means with corresponding SDs or medians with corresponding IQRs. Statistical comparisons between participants allocated to LAAO vs no LAAO were performed using the χ^2^ test or Fisher exact test if the expected cell count in 1 of the 2 arms was less than 5 for categorical variables. For continuous variables, we used the unpaired *t* test or Wilcoxon rank sum test when data did not follow a normal distribution. The mRS scores on day 7 or at discharge were compared with the use of ordinal logistic regression (shift analysis) and reported with the use of common odds ratio (OR) and corresponding 95% CI. The risks for perioperative stroke and 30-day stroke mortality were evaluated with Cox proportional hazards models and reported with corresponding hazard ratios (HRs) and accompanying 95% CIs. For baseline characteristics, distribution of mRS scores, and the risks for perioperative stroke and 30-day stroke mortality, we included only the first stroke. For analyses on subtype and topography, we analyzed all ischemic strokes (first and recurrent events) during the trial follow-up period. To test the consistency of our findings in the primary analysis of the ITT population, we performed a post hoc sensitivity analysis of the per-protocol population, excluding crossovers. *P* values for all comparisons were 2-sided, and statistical significance was accepted at the .05 level. The main data analyses took place from December 18, 2023, to April 29, 2024. All analyses were performed using SAS, version 9.4 (SAS Institute).

## Results

The results of the LAAOS III trial have been previously reported.^9^ Briefly, the mean (SD) age was 71 (8) years, 1552 were female (33%), and 3218 were male (67.5%). The mean (SD) CHA_2_DS_2_-VASc score was 4.2 (1.5), and approximately 80% of participants received oral anticoagulation. A total of 273 incident ischemic strokes occurred in the LAAOS III trial during the mean follow-up period of 3.8 years. The mean (SD) age of participants at the time of the first ischemic stroke was 75 (7) years, 104 were female (38%), and 169 were male (62%). The risk of a first ischemic stroke was significantly lower for participants allocated to LAAO compared with no LAAO (4.6% vs 6.9%; HR, 0.66; 95% CI, 0.52-0.84). A first ischemic stroke occurred in the first 30 days from the cardiac surgery in 112 participants, and the risk for perioperative stroke was comparable between the 2 groups (2.1% vs 2.6%; HR, 0.78; 95% CI, 0.54-1.13).

Participant characteristics of those with a first ischemic stroke are presented in Table 1. History of stroke or transient ischemic attack before trial enrollment was reported in 43 participants (15.8%) and 19 participants (7.0%), respectively. A substantial proportion of the participants were taking antiplatelet (119 [43.6%]) and/or anticoagulant (155 [56.8%]) treatment at the time of the first ischemic stroke. No significant differences were noted between the 2 groups.

**Table 1.** Participant Characteristics With a First Ischemic Stroke

Characteristic	LAAO	No LAAO	*P* value
No. of participants	109	164	NA
Demographics			
Age, mean (SD), y	74.5 (7.3)	75.0 (7.4)	.56
Sex, No. (%)			
Female	42 (38.5)	62 (37.8)	.90
Male	67 (61.5)	102 (62.2)	
Height, mean (SD), cm	168.6 (9.1)	168.9 (9.9)	.85
Weight, mean (SD), kg	81.7 (18.0)	81.6 (15.7)	.98
Coexisting medical conditions			
Stroke, No. (%)	19 (17.4)	24 (14.6)	.53
TIA, No. (%)	5 (4.6)	14 (8.5)	.21
Myocardial infarction, No. (%)	28 (25.7)	29 (17.7)	.11
Rheumatic heart disease, No. (%)	4 (3.7)	12 (7.3)	.21
Heart failure, No. (%)	72 (66.1)	111 (67.7)	.78
Hypertension, No. (%)	94 (86.2)	132 (80.5)	.22
Peripheral arterial disease, No. (%)	24 (22.0)	24 (14.6)	.12
Thromboembolism, No. (%)	3 (2.8)	6 (3.7)	>.99
Diabetes, No. (%)	44 (40.4)	58 (35.4)	.40
Smoking status, No. (%)			
Never	63 (57.8)	81 (49.4)	.15
Current	12 (11.0)	19 (11.6)	.90
Former	33 (30.3)	64 (39.0)	.15
Medications			
ASA, No. (%)	42 (38.5)	68 (41.5)	.84
Other antiplatelets, No. (%)	4 (3.7)	5 (3.1)	.74
Oral anticoagulants, No. (%)	58 (53.2)	97 (59.2)	.55

Abbreviations: ASA, acetylsalicylic acid; LAAO, left atrial appendage occlusion; NA, not applicable; TIA, transient ischemic attack.

**Figure 1.**
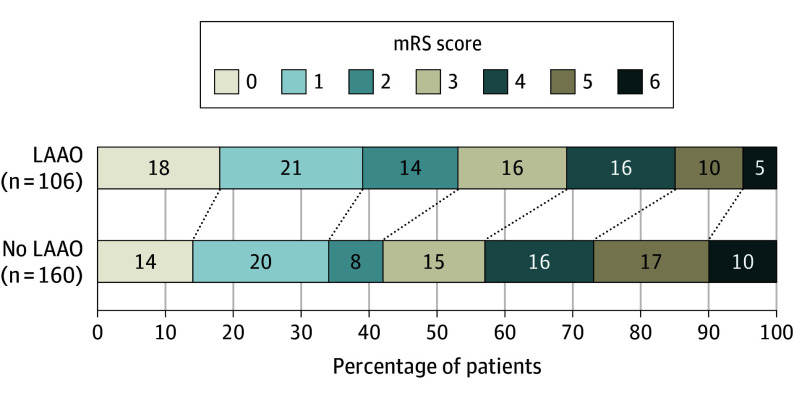
Modified Rankin Scale (mRS) Scores at Day 7 or Discharge After a First Ischemic Stroke LAAO indicates left atrial appendage occlusion.

A total of 266 of 273 participants (97%) had an mRS score at 7 days or discharge from their incident first ischemic stroke. As outlined in Figure 1, participants who were allocated to receive LAAO had reduced (common OR, 0.80; 95% CI, 0.65-0.99) disability at 7 days or discharge after their first ischemic stroke (median [IQR] mRS score, 2 [1-4]) compared with those participants with ischemic stroke in the no-LAAO group (median [IQR] mRS score, 3 [1-5]). Mortality at 7 days or discharge after an ischemic stroke was twice as high for the no-LAAO group compared with the LAAO group (10.0% vs 4.7%; OR, 0.44; 95% CI, 0.16-1.26). Of 273 participants, 51 (18.6%) died within 30 days from their ischemic stroke event. The risk for 30-day stroke mortality was lower in participants allocated to LAAO compared with no LAAO (16.5% vs 20.1%; HR, 0.55; 95% CI, 0.31-0.97).

**Figure 2.**
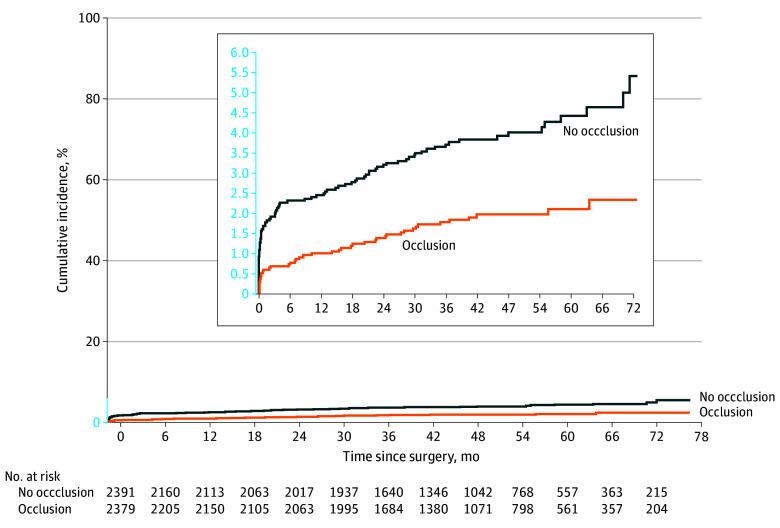
Cumulative Rates of First Cardioembolic Stroke During Follow-Up

Of the 273 participants experiencing a first ischemic stroke event after randomization, 14 in the LAAO group and 23 in the no-LAAO group had subsequent ischemic strokes. Combining first and recurrent ischemic strokes, we had 303 clinical and imaging records of the total 310 events (97.7%) available for adjudication. Those allocated to LAAO had fewer cortical infarcts (46.2% vs 61.3%; difference in proportions, −15.2%; 95% CI, −26.7% to −3.7%; *P* = .01) and more subcortical infarcts (32.5% vs 21.6%; difference in proportions, 10.9%; 95% CI, 0.6%-21.3%; *P* = .04) compared with those without LAAO. Most infarcts occurred in a single vascular territory, and there was no difference in the proportion of infarcts attributed to multiple vascular territories (22.0% vs 24.7%; difference in proportions, −2.7%; 95% CI, −12.5% to 7.1%; *P* = .59) between treatment assignments. A lower proportion of ischemic infarcts in the LAAO group had presumed cardioembolism as the suspected stroke etiology when compared with patients with ischemic infarcts in the no-LAAO group (42.9% vs 57.9%; difference in proportions, −15.1%; 95% CI, −26.5% to −3.7%; *P* = .01). Other suspected stroke mechanisms, including small vessel disease (10.9% vs 7.1%; difference in proportions, 3.8%; 95% CI, −2.9% to 10.6%; *P* = .25), large artery atherosclerosis (6.7% vs 5.5%; difference in proportions, 1.3%; 95% CI, −4.3% to 6.8%; *P* = .65), other etiology (5.0% vs 1.1%; difference in proportions, 4.0%; 95% CI, −0.3% to 8.2%; *P* = .06), or uncertain etiology (34.5% vs 28.4%; difference in proportions, 6.0%; 95% CI, −4.7% to 16.8%; *P* = .27), were comparable between the 2 groups (Table 2). Performing a time-to-event analysis for only the first ischemic stroke events, participants allocated to LAAO had a lower risk for presumed cardioembolic stroke compared with no LAAO (1.9% vs 3.9%; HR, 0.47; 95% CI, 0.33-0.67) (Figure 2). No differences for the other stroke mechanisms between the 2 groups were detected: small vessel disease (0.6% vs 0.5%; HR, 1.08; 95% CI, 0.49-2.37) (eFigure 2 in Supplement 3), large-artery atherosclerosis (0.3% vs 0.3%; HR, 0.75; 95% CI, 0.26-2.15) (eFigure 3 in Supplement 3), other etiology (0.3% vs 0.1%; HR, 3.0; 95% CI, 0.61-14.88) (eFigure 4 in Supplement 3), or uncertain etiology (1.7% vs 2.0%; HR, 0.85; 95% CI, 0.56-1.30) (eFigure 5 in Supplement 3).

**Table 2.** Localization, Vascular Territory, and Subtype of First and Recurrent Ischemic Strokes

Stroke characteristic	No./total No. (%)		Difference in proportion (95% CI)	*P* value
	LAAO	No LAAO		
No. of strokes	119	184	NA	NA
Localization				
Cortical	54/117 (46.2)	111/181 (61.3)	−15.2 (−26.7 to −3.7)	.01
Subcortical	38/117 (32.5)	39/181 (21.6)	10.9 (0.6 to 21.3)	.04
Uncertain	25/117 (21.4)	31/181 (17.1)	4.2 (−5.0 to 13.5)	.36
Vascular territory				
Multiple	26/118 (22.0)	45/182 (24.7)	−2.7 (−12.5 to 7.1)	.59
Single	76/118 (64.4)	107/182 (58.8)	5.6 (−5.6 to 16.8)	.33
Uncertain	16/118 (13.6)	30/182 (16.5)	−2.9 (−11.1 to 5.3)	.49
Subtype^a^				
Cardioembolism	51/119 (42.9)	106/183 (57.9)	−15.1 (−26.5 to −3.7)	.01
Small vessel disease	13/119 (10.9)	13/183 (7.1)	3.8 (−2.9 to 10.6)	.25
Large artery atherosclerosis	8/119 (6.7)	10/183 (5.5)	1.3 (−4.3 to 6.8)	.65
Other defined etiology	6/119 (5.0)	2/183 (1.1)	4.0 (−0.3 to 8.2)	.06
Uncertain	41/119 (34.5)	52/183 (28.4)	6.0 (−4.7 to 16.8)	.27

Abbreviations: LAAO, left atrial appendage occlusion; NA, not applicable.

^a^ Based on modified TOAST (Trial of Org 10172 in Acute Stroke Treatment) criteria.

The findings of the primary analysis in the ITT population were consistent with those of the sensitivity analysis in the per-protocol population. Incident ischemic strokes after LAAO were associated with less disability at 7 days or discharge (common OR, 0.77; 95% CI, 0.61-0.96), lower risk for 30-day mortality (14.4% vs 21.3%; HR, 0.45; 95% CI, 0.24-0.84), fewer cortical infarcts on neuroimaging (47.2% vs 61.2%; difference in proportion, −14.0%; 95% CI, −26.0% to −2.0%; *P* = .02), and less likely to be of presumed cardioembolic origin (44.9% vs 58.7%; difference in proportions, −13.9%; 95% CI, −25.8% to −1.9%; *P* = .02) when compared with ischemic strokes in participants not receiving LAAO (eTable in Supplement 3).

## Discussion

In the LAAOS III trial, we found that LAAO in patients with AF undergoing cardiac surgery decreases the risk for presumed cardioembolic stroke and was associated with less stroke-related disability and mortality. LAAO did not increase the risk for perioperative stroke. The ischemic strokes that occurred in the LAAO group were more likely to be from noncardioembolic sources when the subtype could be determined. Given the higher morbidity associated with cardioembolic strokes compared with other stroke subtypes,^1,2^ it may be possible that the reduction in stroke mortality with LAAO is directly linked to the lower risk of cardioembolism. These findings add meaningfully to the overall reduction in the occurrence of stroke and systemic embolism noted in the primary analysis.^9^

Our findings are in accordance with a retrospective propensity score–matched analysis of the National Readmission Database for the years 2016 to 2020 in the US, in which patients with history of AF and percutaneous LAAO admitted with an ischemic stroke were found to have less severe (OR, 0.69; 95% CI, 0.50-0.96) and fatal (OR, 0.48; 95% CI, 0.26-0.88) events compared with patients with history of AF and without LAAO admitted with an ischemic stroke.^13^

It should be highlighted that the LAAOS III trial evaluated surgical occlusion of the LAA in patients with history of AF undergoing cardiac surgery; therefore, the results of the present analysis cannot be extrapolated to percutaneous LAAO or patients without AF. The efficacy in other patient populations and with percutaneous procedures remains to be established. Trials are in progress to investigate these issues. The Fourth Left Atrial Appendage Occlusion Study (LAAOS-4)^14^ is currently evaluating the efficacy of percutaneous LAAO to prevent ischemic stroke or systemic embolism in participants with AF who remain at high risk of stroke despite treatment with oral anticoagulation. One of the secondary end points of the LAAOS-4 trial is to assess the impact of percutaneous LAAO in addition to best medical therapy on the occurrence of disabling ischemic strokes. The Left Atrial Appendage Exclusion for Prophylactic Stroke Reduction (LeAAPS)^15^ trial is testing left atrial appendage exclusion for the prevention of ischemic stroke or systemic embolism in patients undergoing cardiac surgery at increased risk of AF and ischemic stroke.^16^ The Left Atrial Appendage Closure by Surgery 2 (LAACS-2)^17^ trial is testing LAAO closure to prevent stroke in patients undergoing cardiac surgery irrespective of preoperative AF status and stroke risk.^18^

### Strengths and Limitations

Despite the strengths of the present work, which was the first, to our knowledge, to evaluate the impact of surgical LAAO on stroke mechanism and severity, some limitations need to be acknowledged. Adjudication was performed by stroke neurologists blinded to treatment allocation but was limited by the information provided in clinical and radiological reports. Direct access to source images and all participant medical records was not available. Imaging reports from multicenter trials vary in quality and often lack critical details, which can affect the consistency of the adjudication. Although the process to classify stroke etiologies in this study is practical and has been previously used in other randomized clinical trials,^10,11^ we acknowledge that an algorithmic approach may oversimplify the complex mechanisms underlying ischemic stroke by not accounting for features suggestive of small vessel disease or large artery involvement such as white matter hyperintensities or nonstenotic carotid plaque characteristics, respectively. We would not expect these factors to affect the association between assigned intervention outcomes in a randomized trial. Moreover, attributing small subcortical strokes to small vessel disease as the only pathophysiological mechanism may have contributed to an underestimation of atherosclerotic or cardiac embolism. Specifically in the context of cardiac surgery, embolic material is heterogeneous, and classifying perioperative strokes solely on imaging poses additional challenges. Our algorithm did not account for perioperative mechanisms like aortic clamping or extracorporeal circulation. Again, the specific mechanism of perioperative stroke apart from emboli generated in the LAA would not be expected to differ between randomized groups.

Surgical technique and time were similar in the groups apart from an additional 5-minute bypass time in the LAAO group. If anything, this would be expected to result in fewer complications in the control group. Moreover, in the LAAOS III trial, the dominant method of LAA occlusion was cut and sew, which increased the proportion of open cardiac chamber procedures in the treatment arm compared with the control, theoretically raising the intraoperative risk of cardioembolism from other mechanisms (eg, air, fat). In approximately 30% of the incident strokes, we were unable to determine the presumed etiology. Although this proportion is similar to the proportion of ischemic strokes of unknown etiology in other cohorts,^19^ we acknowledge the possibility of misclassification.

Acute treatment and secondary stroke prevention strategies after the occurrence of ischemic stroke were not systematically collected or analyzed. It should be noted that the LAAOS III trial was blinded, with investigators, patients, and treating physicians being unaware of the treatment allocation, which makes the possibility for systematic biases in therapeutic decisions unlikely. It needs to be acknowledged that acute stroke interventions with thrombolysis or thrombectomy may have been more frequent in the no-LAAO group due to cardioembolic stroke being more often associated with more severe neurological symptoms compared with the LAAO group. However, information on the severity of neurological symptoms at the time of stroke onset is also not available as the National Institutes of Health Stroke Scale was not collected in the LAAOS III trial. There were a relatively small number of events, limiting the statistical power and the ability to perform subgroup analyses. For this reason, we decided to adjudicate and analyze all ischemic strokes (first and subsequent events) when investigating the stroke mechanism in terms of subtyping, location, and vascular territory on neuroimaging. Furthermore, mRS scores were available only at 7 days or discharge, which may be substantially influenced by in-hospital management and poststroke care. As a result, the impact of LAAO in reducing long-term disability after an ischemic stroke remains uncertain. However, it should be noted that discharge mRS score is a significant predictor of 90-day disability for patients with ischemic stroke, and our findings suggest that LAAO was likely associated with a reduction in disability at 90 days.^20^

## Conclusions

In conclusion, in this secondary analysis of the LAAOS III trial, LAAO was associated with a reduction in mortality and disability from ischemic stroke and reduced the proportion of cardioembolic strokes in patients with history of AF undergoing cardiac surgery. Our findings support the recent guidelines from the American College of Cardiology/American Heart Association providing a strong recommendation for LAAO in patients with AF undergoing cardiac surgery,^21^ while providing a rationale for the inclusion of stroke severity as a secondary endpoint in ongoing clinical trials evaluating surgical or percutaneous LAAO for stroke prevention.

## Notes

Supplement 1.
Trial Protocol.

Supplement 2.
Statistical Analysis Plan.

Supplement 3. **eFigure 1.** Algorithm Used for the Adjudication of Stroke Subtype **eFigure 2.** Cumulative Rates of First Stroke Due to Small Vessel Disease During Follow-Up **e Figure 3.** Cumulative Rates of First Stroke Due to Large-Artery Atherosclerosis During Follow-Up **eFigure 4.** Cumulative Rates of First Stroke Due to Other Defined Etiology During Follow-Up **eFigure 5.** Cumulative Rates of First Stroke Due to an Uncertain Etiology During Follow-Up **eTable.** Localization, Vascular Territory, and Subtype of First and Recurrent Ischemic Strokes in the Per-Protocol Population

Supplement 4.
Data Sharing Statement.
