# Supplementary material for: Stroke Mechanism and Severity After Left Atrial Appendage Occlusion: Insights From the LAAOS III Randomized Clinical Trial
Source: JAMA Neurol. 2025 Nov 17;83(1):76–82. doi: 10.1001/jamaneurol.2025.4478 (PMC12624455; doi:10.1001/jamaneurol.2025.4478)
Supplement: Supplement 1. — Trial Protocol. [file jamaneurol-e254478-s001.pdf]

LAAOS III

Left Atrial Appendage Occlusion Study III

Protocol #: LAAOS III-2012

ClinicalTrials.gov ID: NCT01561651

EudraCT #: 2012-001478-27

FINAL PROTOCOL

Date: April 24, 2012 (version 1.0)

Study Coordinating Centre:	Population Health Research Institute<br>DBCVSRI, 237 Barton Street East<br>Hamilton, Ontario, L8L 2X2<br>Canada

This protocol is the confidential intellectual property of the Study Steering Committee and McMaster University and Hamilton Health Sciences (Population Health Research Institute). The use of any unpublished material presented in this document must be restricted to the recipient for the agreed purpose and must not be disclosed to unauthorized persons without the written consent of the LAAOS III Steering Committee.

NAMES AND ADDRESSES OF:

Co-Principal Investigator	Name:	Dr. Stuart Connolly
	Address:	Population Health Research Institute<br>237 Barton Street East, C3-123<br>Hamilton, ON L8L 2X2
	Tel:	905-527-4322 x 40317
	Fax:	905-297-2778
	Email:	Stuart.Connolly@phri.ca

Co-Principal Investigator	Name:	Dr. Richard Whitlock
	Address:	Population Health Research Institute<br>237 Barton Street East, C1-114<br>Hamilton, ON L8L 2X2
	Tel:	905-527-4322 x 40306
	Fax:	905-297-2778
	Email:	Richard.Whitlock@phri.ca

Project Manager	Name:	Ms. Susan Chrolavicius
	Address:	Population Health Research Institute<br>237 Barton Street East, C1-240<br>Hamilton, ON L8L 2X2
	Tel:	905-527-4322 x 40365
	Fax:	905-297-3779
	Email:	Susan.Chrolavicius@phri.ca

Research Coordinator	Name:	Ms. Jessica Vincent
	Address:	Population Health Research Institute<br>237 Barton Street East, C1-239<br>Hamilton, ON L8L 2X2
	Tel:	905-527-4322 x 40635
	Fax:	905-297-3779
	Email:	Jessica.Vincent@phri.ca

**LAAOS III Project Office Email:**	**LAAOSIII@phri.ca**

INVESTIGATOR AGREEMENT PAGE

I,____, Investigator, have examined this protocol

Left Atrial Appendage Occlusion Study III (LAAOS III)

and I have fully discussed the objectives of this trial and the contents of this protocol with the sponsor.

I agree to conduct the study according to this protocol and to comply with its requirements, subject to ethical and safety considerations.

I acknowledge that I am responsible for overall study conduct. I agree to personally conduct or supervise the described clinical study.

I agree to ensure that all associates, colleagues and employees assisting in the conduct of the study are informed about their obligations. Mechanisms are in place to ensure that site staff receives the appropriate information throughout the study.

Investigator Signature: ____

Date: ____

STUDY SYNOPSIS

Title	Left Atrial Appendage Occlusion Study III (LAAOS III)
Principal Investigators	Dr. Stuart Connolly and Dr. Richard Whitlock
Study Objective(s)	The primary objective is to examine the impact of left atrial appendage occlusion on the incidence of stroke or systemic arterial embolism in patients with atrial fibrillation undergoing cardiac surgery over the duration of follow-up.<br>The secondary objectives over the duration of follow-up (unless otherwise specified) are:<br>1) To determine total mortality<br>2) To determine operative safety outcomes (chest tube output in the first 24 hours post-surgery, post-operative re-exploration for bleeding in the first 48 hours post-surgery, and 30-day mortality)<br>3) To determine the incidence of re-hospitalization for heart failure<br>4) To determine the incidence of major bleeding<br>5) To determine the incidence of myocardial infarction
Study Design	An international multicentre RCT of surgical left atrial appendage occlusion or no occlusion in a total of 3,500 patients undergoing cardiac surgery requiring cardiopulmonary bypass.
Study Population<br><br>Main selection criteria:	 <br><u>Inclusion Criteria</u><br>1) Age ≥ 18 years of age<br>2) Undergoing a clinically indicated cardiac surgical procedure with the use of cardiopulmonary bypass<br>3) A documented history of atrial fibrillation or atrial flutter<br>4) Written informed consent<br><br><u>Exclusion Criteria</u><br>1) Patients undergoing any of the following procedures:<br>a. Off-pump cardiac surgery<br>b. Heart transplant<br>c. Complex congenital heart surgery<br>d. Sole indication for surgery is ventricular assist device insertion<br>e. Previous cardiac surgery (re-operation)<br>2) Patients who have had a previous placement of a percutaneous LAA closure device
Total number of subjects:	Total of 3,500 patients
Expected number of centres:	Approximately 60 centres worldwide

Study Intervention	The intervention is occlusion of the LAA which is compared to no LAA occlusion. Occlusion must be performed using either amputation-and-closure (cut and sew), stapler device, or closure from within the LAA. Intraoperative TEE is encouraged to determine successful closure of the appendage
Evaluation Criteria	The primary outcome is the first occurrence of stroke or systemic arterial embolism over the duration of follow-up.<br><br>The secondary outcomes over the duration of follow-up (unless otherwise specified) are:<br>1) Total mortality<br>2) Operative safety outcomes (chest tube output in the first post-operative 24 hours, rate of post-operative re-exploration for bleeding in the first 48 hours post-surgery and 30-day mortality)<br>3) Re-hospitalization for heart failure<br>4) Major bleed<br>5) Myocardial infarction
Statistical Considerations	The intention to treat principle, in which all participants will be included in their assigned treatment groups regardless of actual surgical procedure performed, will guide all analyses. A time to event analysis will be used to test the primary outcome variable. The primary outcome (stroke or systemic arterial embolism) will be presented using Kaplan-Meier survival curves and be compared between groups using a log rank test. The treatment effect as measured by the hazard ratio and 95% confidence interval and adjusted for other covariates will be derived by the Cox proportional hazards model. The secondary and other outcomes will be compared via a t-test, chi-square test, or non-parametric tests where appropriate.
Duration of Study Period (per subject)	Patients will be followed at hospital discharge, 30 days, one year and annually thereafter until the common study end date (to be determined at approximately 5 years after the first patient randomized). Interim telephone calls will be held at the 6-month intervals to maintain contact with the patients.

LIST OF ABBREVIATIONS

Abbreviation	Definition
ACTIVE	Atrial Fibrillation Clopidogrel Trial with Irbesartan for Prevention of Vascular Events
AF	Atrial fibrillation
AFFIRM	Atrial Fibrillation Follow-Up Investigation of Rhythm Management
CABG	Coronary artery bypass grafting
CCORT	Canadian Cardiovascular Outcomes Research Team
$CHADS_2$ Score	Congestive heart failure (1 point), Hypertension (1 point), Age >75 (1 point), Diabetes Mellitus (1 point), Prior Stroke or TIA (2 points)
CK-MB	Creatine kinase myocardial b fraction
CNS	Central nervous system
CORONARY	CABG Off OR ON Pump RevAsculaRization StudY
CPB	Cardiopulmonary bypass
CRF	Case report form
DSMB	Data safety monitoring board
ECG	Electrocardiogram
ED	Endothelial dysfunction
FRACTAL	Fibrillation Registry Assessing Costs, Therapies, Adverse events and Lifestyle study
GCP	Good Clinical Practice
ICU	Intensive care unit
INR	International normalized ratio
IRB	Institutional Review Board
IWRS	Interactive web randomization system
LA	Left atrium
LAA	Left atrial appendage
LAAOS	Left atrial appendage occlusion study
LV	Left ventricle
LVD	Left ventricular dysfunction
LVEF	Left ventricular ejection fraction
MI	Myocardial infarction
OAC	Oral anticoagulant
PCI	Percutaneous coronary intervention
PHRI	Population Health Research Institute
PROTECT AF	Randomized Prospective Trial of Percutaneous LAA Closure vs Warfarin for Stroke Prevention in AF
QVAFS	Questionnaire Verifying Stroke Free Status
RBC	Red blood cells
RCT	Randomized controlled trial
REB	Research Ethics Board
RR	Relative risk
SAE	Serious adverse event
TEE	Transesophogeal Echocardiogram
TIA	Transient ischemic attack
VKA	Vitamin K Antagonist

CONTENTS

List of Abbreviations 6
1 Introduction and Rationale 9
1.1 Problem being addressed 9
1.2 Embolic stroke in atrial fibrillation and the left atrial appendage 10
1.3 Oral anticoagulation 10
1.4 Limitations of oral anticoagulation therapy (which LAA occlusion may mitigate) 11
1.5 Current evidence regarding LAA occlusion 13
1.6 Background summary 14
2 Study Objectives 14
2.1 Primary objectives 14
2.2 Secondary objectives 14
3 Study Design 14
3.1 Type of study 14
3.2 Expected number of subjects 15
3.3 Method of intervention allocation 15
3.4 Methods for protecting against bias 15
3.5 Duration of the study period for each subject 15
4 Study Population 15
4.1 Inclusion Criteria 15
4.2 Exclusion Criteria 16
5 Study Procedures 16
5.1 Intervention 16
5.2 Schedule of visits and observations 16
5.2.1 Study flowchart 16
5.2.2 Visit schedule 17
5.2.3 Baseline Data Collection 17
5.2.4 Cardiac Surgery to Hospital Discharge 17
5.2.5 Follow-up Visits 18
5.3 Selection procedures (entry procedures) 18
5.4 Encouraging compliance 18
5.5 Antithrombotic management 18
5.6 Study outcomes 18
5.6.1 Primary Outcome 18
5.6.2 Secondary Outcomes 19
5.6.3 Definitions of Study Outcomes 19
5.7 Emergency Unblinding 20
6 Statistical Considerations 20
6.1 Analysis population 20
6.2 Statistical methods 20
6.3 Planned subgroup analyses 21
6.4 Data Safety Monitoring Board (DSMB) 21
6.5 Sample size calculation 21
7 Sub-Studies and Ancillary Studies 23
8 Study Organization 24
9 Ethical Standards 24
9.1 Ethical considerations 24
9.2 Informed consent 24

9.3 Ethics Review Committee ... 24
10 Study Monitoring ... 25
10.1 Responsibilities of the investigator(s) ... 25
10.2 Data collection on electronic case report forms (eCRFs) ... 25
11 Administrative Rules ... 25
11.1 Curriculum vitae ... 25
11.2 Confidentiality ... 25
11.3 Record retention in investigating centre(s) ... 25
12 Ownership of Data and Use of the Study Results ... 26
13 Publications ... 26
14 Protocol Amendments ... 26
15 Bibliographic References ... 27
16 Appendix ... 30

1 INTRODUCTION AND RATIONALE

1.1 Problem being addressed

Atrial fibrillation (AF) is an important cause of stroke, responsible for at least one sixth of all strokes in Canada. It is well established that most strokes in patients with AF are cardio-embolic, originating from the LAA (left atrial appendage). Three main approaches to stroke prevention in AF can be conceptualized: 1) Elimination of AF, itself, 2) Prevention of clot formation by medical therapy (either antiplatelet or anticoagulant) and 3) Physical elimination of the LAA, the site of clot formation. To date, elimination (or suppression) of AF has not been effective against stroke, probably because no AF therapy has been able to suppress all AF episodes. Antithrombotic medical therapy has been very effective, but is limited by potential for serious bleeding and by the general problem of continuity of treatment, namely non-prescription, non-compliance, sub-optimal anticoagulation control and treatment withdrawal. The third approach, occlusion or removal of the LAA, is a logical idea that has received considerable recent attention due to positive results from a small trial of device closures. Although the results of this small trial are encouraging, these results are not definitive. They do, however, provide the impetus for further research.

All three approaches to stroke prevention have very different mechanisms of action and it is likely that their effects are complementary and additive, especially those of antithrombotic medical therapy and LAA occlusion. Medical therapy is limited by under-prescription and interruptions (both scheduled and inadvertent). In all these circumstances, reduction of clot formation by removal of the LAA could provide continuous protection. LAA occlusion on the other hand cannot reduce thrombus formation at other sites (e.g., left atrium proper, left ventricle, aorta) which may coexist in AF. Reducing the risk of thrombus formation at these other sites is best managed with systemic antithrombotic therapy. Thus removal of the LAA and systemic antithrombotic therapy are likely to act additively or synergistically.

The LAA is a blind sac attached to the left atrium, which is often narrow and trabeculated. Echocardiographic studies have shown that the LAA is the major site of thrombus formation in patients with AF; with >90% of atrial thrombi occurring in this appendage. A recent small randomized trial of device closure of the LAA in AF patients reported non-inferiority of LAA occlusion to warfarin for a composite of stroke, bleeding and death. These data provide reasonable proof of concept for LAA occlusion, but have failed to convince most practitioners and regulatory authorities as the trial was small, the procedure is complex, serious adverse events were common, and the non-inferiority margin was unusually wide. A definitive randomized trial of LAA occlusion is needed.

Cardiac surgery provides an excellent opportunity to remove the atrial appendage at very low risk. During most cardiac surgery procedures, the LAA is exposed and readily accessible; and LAA removal only takes a few extra minutes. AF is a major cause of stroke and is common in patients requiring cardiac surgery. A large trial to test if opportunistic surgical removal of the LAA at the time of other routine cardiac surgery can reduce stroke in patients with AF is a high priority for 2 reasons: 1) a positive trial will immediately change clinical surgical practice making LAA occlusion a standard part of cardiac surgery which in turn would lead to a large reduction in the stroke burden of patients undergoing cardiac surgery; and 2) it will for the first time provide conclusive evidence that LAA occlusion reduces stroke, greatly stimulating the agenda of further research in this promising area.

1.2 Embolic stroke in atrial fibrillation and the left atrial appendage

AF associated stroke is associated with worse outcomes than those occurring in the absence of AF.[4, 5] Clinical and diagnostic imaging evidence indicates that at least 70% of all strokes in patients with AF are cardio-embolic from the left atrium.[6] An overview of echocardiographic and autopsy studies of atrial thrombus location concluded that at least 90% of left atrial thrombi are found in the LAA.[8] The left atrial appendage has pulsatile flow in sinus rhythm; this disappears in AF resulting in greatly reduced appendage emptying. This stasis together with increased atrial fibrosis typical of AF, and activation of blood coagulation underlie thrombus formation in AF (Virchow's triad). Removal or occlusion of the LAA removes a key component of this triad which may in turn reduce thrombus formation and embolic stroke in AF patients.[9] The atrial appendages however are also a main source of atrial natriuretic peptide which plays a role in salt and water homeostasis; although a small randomized study (n=77) suggested no ill effects of appendage removal.[10]

Currently no adequately powered randomized trial of LAA removal has been done. The PROTECT AF trial was reported last year. It evaluated the Watchman device which is designed to occlude the LAA by delivery of an occluding device over a trans-venous, trans-septal approach.[11] PROTECT AF investigators chose to compare device therapy to warfarin in an unblinded non-inferiority trial using a composite outcome that included bleeding, thrombotic and fatal outcomes. This trial claimed non-inferiority to warfarin but the due to the weak design (small size, unconventional primary outcome and wide non-inferiority margins) it has failed to lead to regulatory approval. An on-going study is enrolling patients but using the same design. This trial design has provided some proof of concept to the occlusion approach but will continue to be limited by the complexity of the non-inferiority design against effective active therapy (warfarin). Recent non-inferiority trials of new oral anticoagulants against warfarin have required enrolments of between 14,000 and 20,000 patients to demonstrate non-inferiority.

If a complex procedure is required to occlude the LAA, it may be most appropriate to do this to replace warfarin, but if the LAA occlusion can be performed at time of routine surgery with almost no risk, then considering that surgical and medical therapies are almost certain to be complementary, it makes most sense to evaluate surgical LAA occlusion as an adjunct to usual medical therapy. Not only does the proposed design of our study overcome the significant limitations and obstacles of an unblinded non-inferiority trial but it innovates in testing the value of combined surgical and medical therapy which has a strong rationale. LAA occlusion and antithrombotic therapy have completely different mechanisms; occlusion removes the anatomic location for most potential cardiac thrombi, while antithrombotic therapy reduces the tendency for thrombi formation. It is a strong hypothesis that the two approaches will additive or synergistic against stroke. Even the most effective antithrombotic therapy needs to be taken once or twice every day over years (even decades) to be fully beneficial; a challenge even to the most compliant patient. LAA occlusion once adequately performed will never re-form and thus will provide un-interrupted protection against thrombus formation, and potentially stroke, for life.

1.3 Oral anticoagulation

Oral anticoagulant (OAC) therapy reduces the risk of stroke in AF and is recommended for stroke prevention in patients with AF who have risk factors for stroke.[12] A Cochrane meta-analyses that included twenty-nine trials and 28,044 patients [13, 14] reported that warfarin reduced the relative risk of stroke by 64% (95% CI, 49% to 74%) compared to no treatment and by 37% (95% CI, 23% to 48%) compared to aspirin. Aspirin is also effective, reducing the relative risk of stroke in AF by

20%. Anticoagulation is now recommended for all higher risk patients with AF. However there are still many patients who only receive antiplatelet therapy. Administrative database surveys indicate that only about two-thirds of patients who might benefit from anticoagulants actually receive one and discontinuation rates of warfarin approach 50% by 3 years.

New oral anticoagulants are being introduced which also reduce stroke in AF; the direct thrombin inhibitor, dabigatran, and the Factor Xa inhibitors rivaroxaban and apixaban. These agents have been evaluated in large clinical trials and have been shown to be non-inferior, and in some cases superior, to warfarin for stroke reduction; with similar or less bleeding. Dabigatran 110 mg, apixaban and rivaroxaban all showed very similar rates of ischemic stroke relative to warfarin, whereas dabigatran 150 mg showed a significant 25% relative risk reduction compared to warfarin. Both Factor Xa inhibitors and both dabigatran doses showed a large reduction in hemorrhagic strokes compared to warfarin. Major bleeding rates on all these agents, however, exceeded 3% per year, and minor bleeding rates were over 10% per year. Thus hemorrhage remains a significant limitation of both old and new oral anticoagulants. One advantage of the new agents is that they do not require monitoring which makes them easier to take than warfarin; but this paradoxically limits the physician's ability to ensure patient compliance.

1.4 Limitations of oral anticoagulation therapy (which LAA occlusion may mitigate)

There are many limitations to OAC therapy: 1) increased risk of bleeding; 2) need for monitoring of coagulation (INR) for warfarin; 3) patient non-compliance, a problem with all chronic medications (see next section below); 4) physician reluctance to prescribe especially to elderly patients; and 5) frequent need for therapy discontinuations for surgery, procedures and diagnostic tests.

Increased bleeding, both major and minor is inherent in all antithrombotic therapy. For example, in the recent RE-LY Trial, the annual rates of major bleeding were 3.4%, 2.7% and 3.1% for dabigatran 110 mg BID, 150 mg BID and warfarin, respectively; and minor bleeding rates were 13%, 15% and 16% per year. Major bleeding is serious. In both ACTIVE and RE-LY trials, major bleeding increased the adjusted risk of death several fold compared to those without bleeding. One of the biggest problems with bleeding is that even minor bleeding may lead to discontinuation of antithrombotic therapy and exposure to stroke risk; a problem that would be mitigated by concomitant LAA occlusion.

The need for monitoring of warfarin therapy makes it very unattractive to patients and because warfarin is difficult to control, it is a major limitation of therapy. Keeping patients in the therapeutic range of the INR is achieved only about half to two thirds of the time even in clinical trials where patients and centres are selected.[15] In typical community practice, the time in therapeutic range falls to about 50% as demonstrated by a recent overview of studies.[16, 17] A low time in range is strongly associated with an increased risk of both stroke and bleeding.[18] Thus a concurrent therapy such as LAA occlusion that reduces stroke and is continuously effective is likely to be beneficial in patients receiving warfarin. LAA occlusion would theoretically provide protection to patients when their INR is non-therapeutic.

Patient non-compliance is a major limitation inherent to OAC therapy. In a major review of medication compliance for cardiovascular disease, Ho and colleagues estimated that 25–55% of patients do not take their chronic cardiac medications as prescribed.[19] Medication adherence for asymptomatic or chronic conditions is typically lower than that for acute or symptomatic conditions, and drops substantially after the initial months of therapy.[19-22] The reasons for this include patient-related factors (e.g., health illiteracy, forgetfulness, socio-economic barriers),

medication-related factors (e.g., cost, complexity of the regimen, side effects) and provider-related factors (e.g., a lack of coordinated care and follow-up).[22-25] Non-adherence is strongly skewed towards under- rather than over-dosing, and is associated with an increased risk of death, disability, hospitalization, and avoidable health care costs.[19, 26-29] A recent study of point of care testing in 53 Australian general practices is instructive. The study included patients who required OAC and only 43% of patients on anticoagulants reported consistent adherence to therapy during the study.[30] There is also substantial evidence that physicians under-estimate the degree of medication non-compliance even in patients who they 'know well'.[31] Compliance issues continue to be a problem with all medications and may be more of a problem with new anticoagulants than with warfarin, due to short half-lives and lack of need to regular monitoring. Clearly LAA occlusion could provide benefit to many patients on medical therapy who are sometimes non-compliant.

The under-use of anticoagulants is widely documented in virtually every country where this has been studied (Table 1).[32-38]. Many patients (up to half) are unsuitable for warfarin for a variety of reasons and some will remain unsuitable for the new anticoagulants. In the CCORT AF study, using prescription claims databases in Alberta, British Columbia and Ontario from 1997 to 2000, less than one-half of AF patients filled a prescription for warfarin within 90 days of discharge for an AF hospitalization.[36] After initiation of warfarin, discontinuation is very common. In one large administrative database registry from the United Kingdom, Gallagher et al reported warfarin discontinuation rates of 50% within a 4 year follow-up period (Figure 1). A very recent analysis of Ontario Drug Benefit claims data in 125,195 patients >65 years with atrial fibrillation who initiated warfarin therapy, found that almost one third (31.8%) discontinued warfarin within 1 year of initiation, and the median time to discontinuation was 2.9 years (Tara Gomes, University of Toronto, personal communication). The main limitation of warfarin is concern about bleeding and this often prevents its use in otherwise suitable patients.[39, 40] This suggests that even with the new anticoagulants, non-use and discontinuation of anticoagulants will be a problem; one that can be mitigated potentially by LAA occlusion.

Table 1. Use of Oral Anticoagulant Therapy to Prevent Stroke in AF: Results of Recent Surveys

Year	Study	Published Survey Population	Treated With Warfarin, % (Patient Status)
1999	ATRIA Study[51]	11 082 US patients large health maintenance organization without contraindications	60 (high-risk patients)
2005	NABOR Study[52]	945 US patients from teaching, community, and VA hospitals	55 (high-risk patients)
2006	Euro Heart Survey[53]	2706 outpatients in 35 European countries	64
2006	Hylek et al[54]	402 US patients, _65 years old, not on warfarin at admission to teaching hospital	51 (discharged on warfarin)
2006	Birman-Deych et al[55]	16 007 US Medicare patients	49
2007	Glazer et al[56]	437 newly detected AF patients at high risk of stroke	59%
2011	Mercaldi et al[57]	119 764 nonvavlular AF Medicare patients	58.5%

ATRIA indicates Anticoagulation and Risk Factors in Atrial Fibrillation; NABOR, National Anticoagulation Benchmark and Outcomes Report.

Figure 1

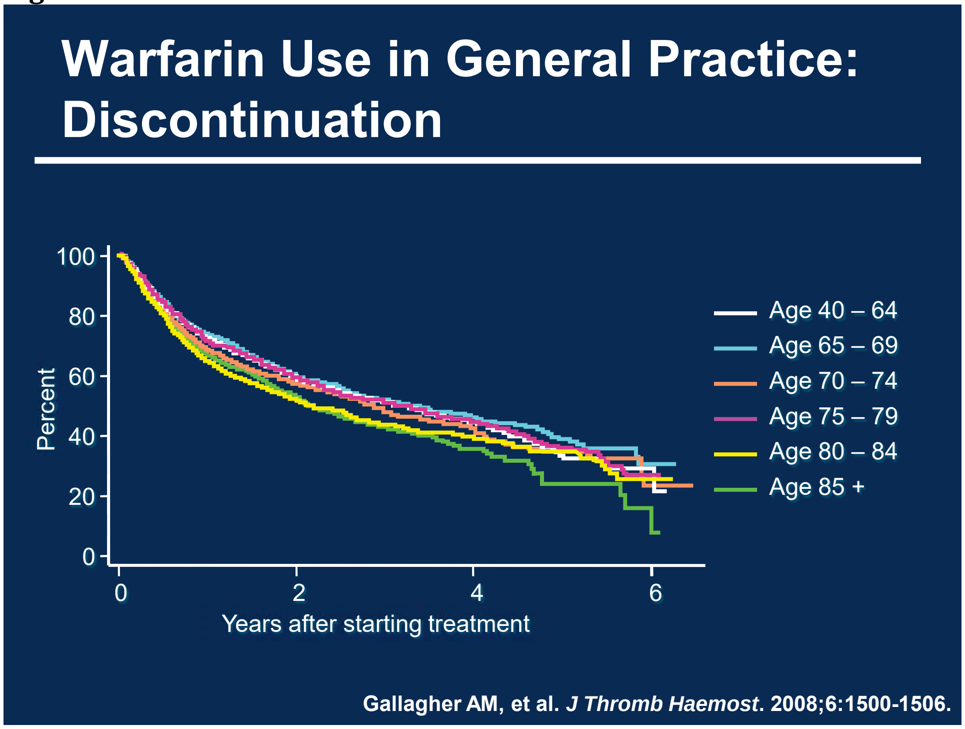

Interruption of anticoagulant therapy for surgery, procedures and diagnostic tests is very common in patients with AF. In the RE-LY Trial, dabigatran and warfarin patients were off of their anticoagulant study medication 13.6% of the time during the two years of follow up. Considering that these patients were being followed very closely by a dedicated study nurse and investigator who encouraged study medication compliance and re-initiation of therapy after a discontinuation, it is likely that rates of anticoagulation non-compliance are much greater in usual clinical practice; and LAA occlusion can potentially be very useful in this situation.

The fundamental limitations of OAC therapy and how LAA occlusion might mitigate these have been detailed. It is also important to recognize that LAA occlusion is not a panacea and that it might not be a suitable stand-alone therapy. AF is associated with a systemic hyper-coagulable state. Platelet function is enhanced with increased plasma levels of thromboglobulin and platelet factor 4. Systemic markers of activation of the coagulation cascade, such as thrombin-antithrombin II complex, D-dimers, fibrinogen, and prothrombin fragments 1 and 2, are also increased. Although most thrombi form in the left atrial appendage, some likely come from aortic plaque, the left ventricle and elsewhere. Thus a systemic antithrombotic therapy is likely a very good complement to a focused surgical intervention that targets only one source of embolism, albeit the most important one.

1.5 Current evidence regarding LAA occlusion

Prior to the publication of PROTECT AF, this literature was dominated by observational studies. It was upon these observational data that the America Heart Association based its recommendation to occlude the LAA in AF patients undergoing mitral valve surgery.[42, 43] In a retrospective study examining 205 patients post mitral valve surgery, the success rate of LAA closure when attempted approached 90%. Multivariate analysis demonstrated the absence of LAA ligation as an independent predictor of occurrence of an embolic event (OR 6.7, 95% CI 1.5-31.0). Results from

case series of Maze procedure patients are also often cited to support the amputation of the LAA.[44] The Maze procedure attempts to eliminate AF through a series of cuts in the right and left atria, suturing them closed, and excising both atrial appendages in a similar fashion. Cox et al. published a case series of 306 patients who underwent a "cut and sew" Maze procedure.[45] Rates of stroke were low but the majority of patients (n=162) were very low risk. Ultimately, these and several other small observational studies cannot provide the level of evidence needed to clearly answer this important question no substantially change clinical practice.

1.6 Background summary

In summary, it is hypothesized that LAA occlusion will reduce stroke and will benefit virtually all AF patients if it can be performed at very low-risk at the time of routine cardiac surgery. A positive result of an adequately powered and carefully executed clinical trial of surgical LAA occlusion versus no occlusion would also be the first unequivocal demonstration of the effectiveness of LAA occlusion. A positive trial would likely lead to almost universal adoption of this procedure at time of cardiac surgery, because it takes little time to perform and it is an easy procedure for any cardiac surgeon.

2 STUDY OBJECTIVES

2.1 Primary objectives

The primary objective is to examine the impact of LAA occlusion on the incidence of stroke or systemic arterial embolism in patients with atrial fibrillation undergoing cardiac surgery with the use of cardiopulmonary bypass.

2.2 Secondary objectives

The secondary objectives are over duration of follow-up (unless otherwise specified) are:

1) To determine total mortality

2) To determine post-operative safety outcomes (chest tube output in the first 24 hours post-surgery, post-operative re-exploration for bleeding within 48 hours post-surgery, and 30-day mortality).

3) To determine the incidence of re-hospitalization for heart failure.

4) To determine the incidence of major bleeding.

5) To determine the incidence of myocardial infarction.

3 STUDY DESIGN

3.1 Type of study

An international, multicenter, randomized blinded trial of surgical left atrial appendage occlusion in patients with atrial fibrillation/flutter who are undergoing cardiac surgery.

3.2 Expected number of subjects

The total sample size for the study is 1,750 patients per group, for a total of 3,500 patients. Patient will be recruited from approximately 60 centres worldwide.

3.3 Method of intervention allocation

Eligible and consenting patients will be randomized via the central interactive web randomization system (IWRS) at the Population Health Research Institute. Each patient will be assigned in a blinded fashion to one of 2 groups (left atrial appendage occlusion or no left atrial appendage occlusion) according to a computer generated randomization list. Patients will be considered randomized when the intervention allocation has been provided through the IWRS. The confidential allocation email will be sent to the participating surgeon's email to maintain blinding of all others associated with the study.

3.4 Methods for protecting against bias

The patient and all study personnel, except the surgeon performing the cardiac surgery, will be blinded to the assigned allocation. This includes investigators (if other than the cardiac surgeon), study coordinators, other study team members, PHRI Project Office staff, and any other medical personnel involved in the patient's care. Although the surgeon will know the allocation, he/she must agree to not be involved in the ongoing antithrombotic management of the patient or in any of the post-operative data collection. Surgeons typically have little say in the on-going antithrombotic therapy of patients on whom they operate for reasons unrelated to the AF.

Blinding to intervention will be achieved as follows: the operative report and the dictated surgical report will only note that the patient has been enrolled in the LAAOS III trial and has been randomized to either left atrial appendage occlusion or no left atrial appendage occlusion. Data collection related to the intervention allocation and the details of the surgery performed will be maintained in a separate unblinded confidential database.

3.5 Duration of the study period for each subject

Although the study intervention occurs intra-operatively, all patients will be followed from the time of randomization until the final follow-up visit. Following randomization and baseline data collection, visits will occur at hospital discharge, 30 days, one year and annually thereafter until the common study end date (to be determined at approximately 5 years after the first patient randomized). Interim telephone calls will be held at the 6-month intervals to maintain contact with the patients.

4 STUDY POPULATION

4.1 Inclusion Criteria

1) Age ≥ 18 years of age
2) Undergoing a clinically indicated cardiac surgical procedure with the use of cardiopulmonary bypass
3) A documented history of atrial fibrillation or atrial flutter
4) Written informed consent

4.2 Exclusion Criteria

1) Patients undergoing any of the following procedures:
    a. Off-pump cardiac surgery
    b. Heart transplant
    c. Complex congenital heart surgery
    d. Sole indication for surgery is ventricular assist device insertion
    e. Previous cardiac surgery (re-operation)
2) Patients who have had a previous placement of a percutaneous LAA closure device

5 STUDY PROCEDURES

5.1 Intervention

The intervention under investigation is surgical occlusion of the left atrial appendage which is compared to no left atrial appendage occlusion. The trial will permit the following techniques of LAA occlusion: 1) amputation of the LAA and closure 2) stapler closure of the LAA; and 3) closure of the LAA from within the left atrium. The preferred technique is amputation and closure as demonstrated by the video found at http://www.youtube.com/watch?v=aPoeoAhIjGw.

Intraoperative TEE is encouraged to determine successful closure of the appendage. Successful occlusion is defined as TEE Doppler assessment demonstrating an absence of flow across the suture line and a stump of <1 cm. If the closure is not successful by this definition, additional maneuvers should be performed to rectify (e.g. additional sutures, additional staple line) as long as the surgeon feels that it is safe to do so. In patients with pre-operative appendage thrombus, the LAA must be opened to surgically remove the thrombus prior to the occlusion. . A MAZE procedure can be performed; however, if randomized to non-occlusion, the LAA must not be occluded.

5.2 Schedule of visits and observations

5.2.1 Study flowchart

Eligibility Assessment
↓
Written Informed Consent
↓
Randomization
↓
Baseline Data Collection
↓
Cardiac Surgery with LAA Occlusion vs. No LAA Occlusion
↓
Hospital Discharge
↓
30-Day Visit
↓
Annual Visits until Final Follow-up Visit
Telephone Calls at 6 month Intervals
(Study End Date to be determined at approximately 5 years after first patient randomized)

5.2.2 Visit schedule

Table 2: Schedule of visits.

	In-hospital Phase			Follow-up Phase			
	Baseline (Pre-op)	Cardiac Surgery	Hospital Discharge (Post-op)	30-Day Clinic Visit	6-Month, 1.5, 2.5, 3.5-Year Telephone Visits[1]	1, 2, 3, 4, 5-Year Clinic Visits[1]	Final Clinic Follow-up Visit
Eligibility assessment	✓						
Informed consent	✓						
Patient demography	✓						
Medical/surgical history	✓						
Concomitant medications	✓		✓	✓	✓	✓	✓
INR (if applicable)	✓			✓		✓	✓
Vital signs	✓						
Randomization	✓						
ECG	✓[2]						
Details of surgical procedure		✓					
TEE (recommended)		✓					
Outcome events		✓	✓	✓	✓	✓	✓
Untoward medical events		✓	✓	✓	✓	✓	✓
QVSFS[3]						✓	✓

[1] As required until common end date
[2] Pre-operative ECG can be within 30 days prior to surgery (most recent ECG should be recorded)
[3] Questionnaire Verifying Stroke Free Status

5.2.3 Baseline Data Collection

Key baseline characteristics such as demographics, medical history, cardiovascular anatomy, co-morbidities, concomitant medications as well as patient's eligibility criteria, will be collected on the Case Report Forms (CRFs) for all randomized patients. ECGs, serum creatinine, and other routine blood work should be obtained within 30 days prior to surgery.

5.2.4 Cardiac Surgery to Hospital Discharge

Research personnel will provide the operative details (except the blinded study allocation) including the type of cardiac surgery performed and other important information on the Operative CRF. Details of the ICU stay, including the dates and times of ICU admission and discharge, chest tube drainage, transfusions, and other clinical events will be recorded on the ICU CRFs. Patients will be assessed at hospital discharge for clinical events, concomitant and discharge medications as well as total hospital stay. Any primary or secondary outcomes occurring during the initial hospitalization will be noted on the hospital discharge CRFs and recorded on corresponding special CRFs.

The unblinded surgeon will be required to provide details regarding intervention compliance in a separate confidential database to maintain the study blind. Details of technique used (i.e. amputation and closure, stapler device, or closure from within the LAA), and whether or not the occlusion was successful (as defined in Section 5.6.3) will be required for those patients who have undergone the LAA occlusion.

5.2.5 Follow-up Visits

Follow-up will occur at 30 days after randomization, at one year and annually thereafter until the final follow-up visit (the common study end date is expected to be determined at approximately 5 years after the first patient is randomized). Patients will be contacted at 6 month intervals by telephone to avoid patients lost to follow-up. Event CRFs should be completed as soon as the investigating site becomes aware of the event. Supporting documentation for each event is required and should be forwarded to the LAAOS III Project Office as soon as it is available to ensure timely adjudication of events.

The validated Questionnaire for Verifying Stroke Free Status (QVSFS) will be administered at the yearly visits until the Final Follow-up Visit.

5.3 Selection procedures (entry procedures)

After eligibility is confirmed, patients will receive complete information about the study both orally and in writing and will be approached to provide written informed consent. Patients should be randomized just prior to surgery if possible. During the randomization process, the intervention allocation will be provided to the surgeon in a confidential manner.

5.4 Encouraging compliance

Intervention non-compliance is not expected to be a major issue in this trial. The intervention is simple and occurs at a single time point and is controlled by the surgeon, outcomes are objective, and data forms are concise. Patients are generally followed by their surgeons/cardiologists for several years after their procedure. Surgeons are permitted to use their occlusion method of choice for patients randomized to this arm and therefore compliance with the allocated intervention is expected to be high. However, we do foresee circumstances where the surgeon may elect not to occlude the appendage (e.g. unrecognized adhesions or other anatomical considerations). Pilot work suggests that the frequency of such an occurrence will be minimal (less than 1% of cases).

The 6 month interim telephone calls will assist in avoiding patients lost to follow-up by enhancing compliance to the visit schedule.

5.5 Antithrombotic management

Because the surgeon is aware of the occlusion intervention, he/she will leave the management of antithrombotic therapy for AF to the cardiologist, primary care physician or an anticoagulation clinic, ensuring the usual pattern of care for the management of the anticoagulation.

5.6 Study outcomes

5.6.1 Primary Outcome

The primary outcome is the first occurrence of stroke or systemic arterial embolism over the duration of follow-up.

5.6.2 Secondary Outcomes

The secondary outcomes over the duration of follow-up (unless otherwise specified) are:

1) Total mortality

2) Operative safety outcomes (chest tube output in the first post-operative 24 hours, rate of post-operative re-exploration for bleeding in the first 48 hours post-surgery and 30-day mortality)

3) Re-hospitalization for heart failure

4) Major bleed

5) Myocardial infarction

5.6.3 Definitions of Study Outcomes

Stroke
Diagnosis of stroke will require new focal neurological symptoms with rapid onset, lasting at least 24 hours. All strokes will be classified as definite ischemic, definite hemorrhagic or type uncertain.

Systemic Arterial Embolism
Systemic arterial embolism will be judged to occur where there is a clinical history consistent with an acute loss of blood flow to a peripheral artery (or arteries), which is supported by objective evidence of embolism.

Major bleed
Defined as per ISTH: 1) Fatal bleeding, and/or 2) Symptomatic bleeding in a critical area or organ, such as intracranial, intraspinal, intraocular, retroperitoneal, intra-articular or pericardial, or intramuscular with compartment syndrome, and/or 3) Bleeding causing a fall in hemoglobin level of 2.0 g/dL or more, or leading to transfusion of two or more units of whole blood or red cells.

Hospitalization with Heart Failure
Re-hospitalization with an overnight stay or prolongation of an existing hospitalization due to heart failure which requires both clinical (i.e. any of the following signs: elevated jugular venous pressure, respiratory râles, crepitations, or presence of S3) and radiographic evidence (e.g. vascular redistribution, interstitial pulmonary edema, or frank alveolar pulmonary edema).

Efficacy of Occlusion Technique
Successful occlusion is defined as TEE Doppler assessment demonstrating an absence of flow across the suture line and a stump of <1 cm.

Sub-Classification of Death
All deaths will be classified as either cardiovascular or non-cardiovascular. Cardiovascular death is defined as any death with a cardiovascular cause and includes those deaths occurring within 30 days of a cardiovascular procedure (e.g. cardiac surgery, percutaneous transluminal coronary angioplasty), cardiac arrest, myocardial infarction, pulmonary embolus, stroke, hemorrhage, or deaths due to an unknown cause. Non-cardiovascular death is defined as deaths due to a clearly documented non-cardiovascular cause (e.g. trauma, infection, malignancy). The research personnel will forward the Event Adjudication Committee all relevant clinical notes, laboratory tests, diagnostic imaging reports, and autopsy information from any participant who dies.

Myocardial Infarction
Perioperative MI (<48 hours post-operatively) is defined as the presence of new Q-waves or a new left bundle branch block on electrocardiogram, combined with a biomarker (CK-MB or troponin) elevation of at least 5 times the upper reference limit. Late MI (>48 hours) is defined as ischemic symptoms, ECG changes consistent with myocardial infarction (new significant Q waves in two contiguous leads) or evolving ST-segment or T-wave changes in two contiguous leads signifying ischemia or new left bundle branch block (LBBB) or ST segment elevation and elevated cardiac markers (troponins or CK-MB) in the necrosis range. Myocardial injury occurring after a percutaneous coronary intervention (PCI) are included in the late perioperative MI group but are defined as elevation of cardiac markers at least 3 times upper limit of normal (ULN) within 24 hours of percutaneous coronary intervention (PCI) or characteristic evolution of new ECG changes.

Transfusion Requirements
Autologous blood, homologous processed red blood cells, whole blood, plasma, platelets, cryoprecipitate will be recorded for 24 hours after surgery.

24-Hour Chest Tube Output
Total chest tube output in the first 24 hours or until the tubes are removed, whichever comes earlier.

5.7 Emergency Unblinding

Legitimate but rare situations such as an unexpected serious adverse event may require unblinding. We recommend that all unblinding decisions be made jointly with the Project Office. If the local study investigator believes emergency unblinding is essential for the patient's management then it can be undertaken either through:

a) the unblinded surgeon; or
b) the PHRI toll-free unblinding help-line

The principal investigator should approve the request for unblinding wherever possible. Further details on unblinding will be provided in the Manual of Operations.

6 STATISTICAL CONSIDERATIONS

6.1 Analysis population

All randomized patients will be included in the analysis according to the allocated intervention at randomization). At least 1,750 patients will be equally allocated to the LAA occlusion arm or no LAA occlusion arm.

6.2 Statistical methods

The intention-to-treat principle, in which all participants will be included in their assigned treatment groups regardless of actual surgical procedure performed, will guide all analyses. A time-to-event analysis will be used to test the primary outcome variable. The primary outcome (stroke or systemic arterial embolism) will be presented using Kaplan-Meier survival curves and the treatment effect as measured by the hazard ratio and 95% confidence interval will be derived by the Cox proportional hazards model. A p-value of <0.05 for the proportional hazards model will be considered as significant. The proportional hazards assumption will also be tested by graphical

means. This analysis will also be performed on the secondary outcomes of 'death and primary outcome'. All other secondary outcomes will be compared via a t-test, chi-square test, or non-parametric tests where appropriate. The primary outcome will be analyzed at a mean follow-up of 4 years.

6.3 Planned subgroup analyses

Additional Cox models will be used to evaluate interactions between treatment and subgroups of interest: antithrombotic used, amputation and closure technique versus other, successful occlusion by trial definition, $CHADS_2$ score, LA dimension, and Maze procedure. The primary analysis will be repeated secondarily as a per protocol analysis.

6.4 Data Safety Monitoring Board (DSMB)

The independent Data Safety Monitoring Board (DSMB) will ensure patient safety, receive and review interim analyses of efficacy data, provide feedback to the Steering Committee, and ensure the study follows the highest standards of ethics. Over the median follow-up of 4 years, we expect 280 primary outcome events. Two formal interim analyses will be undertaken when 50% (140 events) and 75% (210 events) of the expected events have occurred. Conservative statistical guidelines for data monitoring have been developed and will follow the modified Haybittle-Peto rule. For efficacy, reductions in events of ≥ 4 SD in the first interim analysis and ≥ 3 SD in the second will be used. To be considered significant these predefined boundaries will have to be exceeded in two consecutive analyses performed three or more months apart. Given the extremeness of the monitoring boundaries and the paucity of interim analyses, no adjustment will be made to the final p-value at the trial end. The DSMB in making a recommendation for early stopping will also consider the consistency of the secondary endpoints and any relevant external data. For safety, increase in the rates of the primary outcome of ≥ 3 SD (first look) and ≥ 2 SD (second look) will be used as a trigger for discussion of early stopping and reporting. A decision to continue or stop the trial would be based on a number of factors in addition to the statistical significance of the main results, including consistency of the pattern of the data over time and an assessment of net benefit-risk ratios. At any time during the study, if safety concerns arise the DSMB chairperson will assemble a meeting of the full committee. The DSMB will make their recommendations to the steering committee after considering all the available data and any external data from relevant studies.

6.5 Sample size calculation

This study will enroll 3,500 patients with an average follow-up of 4 years which will allow us to detect a 30% relative risk reduction (RRR) in the primary outcome with an expected control event rate of 2.5% per year. This trial would have 83% power, accounting for a 2%/year loss of patients due to competing death. This sample size is contingent on reasonable assumptions about the patient risk and the types of antithrombotic therapy that patients will receive during follow up. If the event rates are lower than expected, follow-up can be extended with this study design. Table 3 presents required sample size by effect size and power.

Table 3: Sample Size for Primary Outcome

Reduction in Hazard Ratio	Power		
	80%	85%	90%
30%	3170	3630	4240
35%	2260	2580	3020

Assumes proportional hazards model with control arm outcome rates of 2.5% per year, 2 year enrollment and mean 4 year follow up period, 2% per year mortality/lost rate

The enrolment requirement of this trial depends primarily on two parameters: the expected event rate in the control arm and the treatment effect expected from LAA occlusion. We can estimate the event rate in the control arm of the study from the event rates on various antithrombotic treatments in recent trials (Table 4) if we have a good estimate of the $CHADS_2$ score. We have performed a registry of 1886 patients in which we observed that the mean $CHADS_2$ score of patients with AF coming to cardiac surgery was 2.3. (**Assumption 1)**.

Table 4: Annual rate of stroke or systemic embolism in current antithrombotic trials

Agent, Trial, (n of arm)	Stroke or systemic embolism (annual rate)
Aspirin, ACTIVE A (3782)	3.7%
Aspirin, AVERROES (2791)	3.5%
Aspirin and Plavix, ACTIVE A (3772)	2.8%
Apixaban, ARISTOTLE (9120)	1.3%
Apixaban, AVERROES (2808)	1.6%
Warfarin, ACTIVE W (3371)	1.5%
Warfarin, RELY (6022)	1.7%
Warfarin , ARISTOTLE (9081)	1.6%
Dabigatran 150 mg, RELY (6076)	1.1%
Dabigatran 110 mg, RELY (6015)	1.5%

Table 5 shows the expected treatment effects of LAA occlusion in different sub-groups of patients expected to be enrolled into the study. As can be appreciated from Table 5, to properly estimate the control event rate we also need to estimate the rate of use of different antithrombotic medications during follow up. Numerous surveys indicate that oral anticoagulants are used in only about 50% to 60% of high-risk patients with AF due to difficulties with control of the INR, bleeding risk, patient reluctance and physician behavior. The use of oral anticoagulants will tend to increase over the next few years as the new anticoagulants are introduced; however, there will still remain a substantial number of patients who either take aspirin or no therapy due to refusal to take an anticoagulant, difficulty with INR management, high cost of new anticoagulants, development of renal failure which increases the risk of anticoagulation. Therefore we estimate that the number of patient-years of follow on aspirin or no antithrombotic therapy will be 35% ±5%. **Assumption 2.** We have very good estimates of the rate of stroke or systemic embolism for these patients from ACTIVE A and AVERROES (3.7% per year on aspirin and 5.1% on no antithrombotic therapy)

Table 5: Expected event rates for primary outcome and relative risk reduction with LAA occlusion on top of usual care

Therapy Component of usual care	% patients years on therapy component	Control rate of primary outcome per year	Expected relative risk reduction with LAA occlusion	Treatment rate of primary outcome per year
No antithrombotic	5	5.1	63%	1.9
Aspirin	30	3.7	63%	1.4
Warfarin	45	1.7	25%	1.3
Novel anticoagulant	20	1.5	25%	1.1
Overall Usual Care	100	2.5	36%	1.6

Because of cost issues and familiarity, we estimate that warfarin and other Vitamin K antagonists will remain the most common oral anticoagulants used (45% of patient-years of follow up). There will be gradually increasing use of dabigatran and the Factor 10a inhibitors over the next 5 years. It is estimated that 20% of patient years of follow-up will be on dabigatran, rivaroxaban, or apixaban. We estimate, based on the recent large trials, that the primary event rate in control patients taking warfarin will be 1.7% per year and in those taking one of the new anticoagulants it will be 1.5% per year. Thus the overall annual event rate in the control arm without LAA occlusion is estimated at 2.5% per year.

This study is powered to have 83% power to detect a 30% relative risk reduction. A 30% treatment effect is reasonable because the PROTECT AF trial of device closure suggests that the effect of LAA occlusion is similar to that of warfarin, although the mechanism is obviously different and the effect of LAA occlusion will be additive to that of medical therapy. Table 5 shows that the largest effect will likely occur in those receiving no therapy or aspirin. The most recent data comparing an oral anticoagulant to aspirin in AF patients comes from AVERROES, where the reduction in ischemic stroke with apixaban compared to aspirin was 63% (HR = 0.37 (95% CI 0.25–0.55) p <0.001. For patients prescribed an oral anticoagulant, the treatment effect of LAA occlusion will be more modest but not trivial. The benefit of the surgical removal of the LAA will occur during warfarin therapy when patients are out of target therapeutic range (30-50% of time) and during therapy with any oral anticoagulants when there is non-compliance (which is very common); and interruptions for procedures and surgery, which are also common. In RE-LY, 25% of patients had at least one interruption of therapy for procedures. Overall in RE-LY patients were off study medication 14% of time. Based on these considerations a 25% relative reduction in stroke or systemic embolism with LAA occlusion in patients prescribed with oral anticoagulants is reasonable. The overall treatment effect of LAA occlusion on top of usual care is the blended total of these rates which is a 36% relative risk reduction. For the purpose of this trial, we plan to have sufficient statistical power to detect a reduction of 30%.

7 SUB-STUDIES AND ANCILLARY STUDIES

The economic analysis of LAAOS III will include the assessment of LAA occlusion costs, stroke, and systemic arterial embolism over the long-term follow-up. Our hypothesis is that left atrial appendage occlusion will be a dominant strategy i.e. clinically effective and cost-saving. Therefore, the economic analysis will focus on the cost of each surgical procedure in the participating countries (which can vary depending on the choice of occlusion method; suture versus device) and the cost of stroke in each group. The details of the economic analysis protocol are documented in the Appendix.

8 STUDY ORGANIZATION

The trial will be conducted internationally and coordinated at the Population Health Research Institute (PHRI) at McMaster University, Hamilton, Canada. The Steering Committee will be responsible for the design, execution, analysis, and reporting of the study, and will assign appropriate responsibilities to the other study committees. This committee will convene regularly (at least every 3 months) by telephone conference or meetings to address policy issues and to monitor study progress, execution and management. The Steering Committee will include the Principal Investigators and National Leaders from each participating country. The Operations Committee (a subset of the Steering Committee) will hold the primary responsibility for publication of the study results on behalf of the LAAOS III investigators. The Events Adjudication Committee will review all reported outcome events and are blinded to the intervention allocation when reviewing all events.

9 ETHICAL STANDARDS

9.1 Ethical considerations

The study will be conducted in accordance with Good Clinical Practice (GCP), all applicable subject privacy requirements, and the guiding principles of the declaration of Helsinki, including, but not limited to:

- Institutional Review Board (IRB)/Independent Ethics Committee (IEC) review and approval of study protocol and any subsequent amendments.
- Subject informed consent.

Written informed consent must be obtained from each subject prior to participation in the study.

9.2 Informed consent

It is the responsibility of the Investigator to obtain informed consent in compliance with national requirements from each subject prior to entering the trial or, where relevant, prior to evaluating the subject's suitability for the study.

The informed consent document used by the Investigator for obtaining subject's informed consent must be reviewed and approved by the PHRI prior to Ethics Review Committee or similar body (IRB, REB) submission.

9.3 Ethics Review Committee

The Investigator must submit this protocol to an Ethics Review Committee or a similar body (IRB, REB) and is required to forward a copy of the written approval/advice signed by the Chairman to the PHRI. On the approval/advice sheet, the trial name and protocol version, the study documents (protocol and informed consent material) and the date of the review should be clearly stated.

10 Study Monitoring

10.1 Responsibilities of the investigator(s)

The Investigator(s) undertake(s) to perform the study in accordance with Good Clinical Practice. The Investigator is required to ensure compliance with respect to the visit schedule and procedures required by the protocol. The Investigator agrees to provide all information requested in the Case Report Forms in an accurate and timely manner according to instructions provided.

10.2 Data collection on electronic case report forms (eCRFs)

It is the responsibility of the Investigator to prepare and maintain adequate and accurate eCRFs which have been provided by the study to record all observations and other data pertinent to the clinical investigation. All eCRFs should be completed in their entirety and in a timely fashion.

11 Administrative Rules

11.1 Curriculum vitae

An updated copy of the curriculum vitae for each Investigator and co-Investigator will be provided to the PHRI prior to the beginning of the study.

11.2 Confidentiality

All goods, materials, information (oral or written) and unpublished documentation provided to the Investigators, inclusive of this protocol, the patient Case Report Forms are the exclusive property of the Project Office. They may not be given or disclosed by the Investigator or by any person within their authority either in part or in totality to any unauthorized person without the prior written formal consent of the PHRI.

It is specified that the submission of this protocol and other necessary documentation to the Ethics Review Committee or a like body is expressly permitted, the Ethics Committee members having the same obligation of confidentiality.

The Investigator shall consider as confidential and shall take all necessary measures to ensure that there is no breach of confidentiality in respect of all information accumulated, acquired or deduced in the course of the trial, other than that information to be disclosed by law.

11.3 Record retention in investigating centre(s)

The Investigator must maintain all study records, patient files and other source data for the maximum period of time permitted by the hospital, institution or private practice and must be consistent with national regulatory requirements and ICH guidelines. It is recommended that the Investigator retain the study documents at least fifteen (15) years after the completion or discontinuation of the Clinical Trial. However, applicable regulatory requirements should be taken into account in the event that a longer period is required.

12 Ownership of Data and Use of the Study Results

The PHRI Project Office and the Steering Committee of the study have the ownership of all data and results collected during this study. In consequence, the PHRI Project Office reserves the right to use the data of the present study, either in the form of CRFs (or copies of these), or in the form of a report, with or without comments and with or without analysis, in order to submit them to the Health Authorities of any country. The Steering Committee has full rights to publication based on data from this study, without restriction.

13 Publications

All analyses for publication will be provided by the PHRI Project Office. The main responsibility for presentations and/or publications belongs to the Operations Committee. Publication of the main findings of this study will be made jointly in the name of all wholehearted collaborators. Other papers will be authored based on the contributions of the individuals to the overall study. All the trial participants (Investigators and committee members) make a prior delegation of responsibility for primary presentation and/or primary publication of the results to the Operations Committee. No other publication is allowed before the primary publication. Any presentation or publication by any trialist must mention the trial and has to be approved by the Steering Committee. Moreover, it is mandatory to make reference to the primary publication.

14 Protocol Amendments

It is specified that the appendices attached to this protocol and referred to in the main text of this protocol, form an integral part of the protocol. The Investigator should not implement any deviation from, or changes of the Clinical Trial Protocol without agreement by the Sponsor and prior review and documented approval/favorable opinion from the IRB/IEC of an amendment, except where necessary to eliminate an immediate hazard(s) to Clinical Trial Patients, or when the change(s) involves only logistical or administrative aspects of the trial. Any changes agreed upon will be recorded in writing, the written amendment will be signed by the Investigator and by the Sponsor and the signed amendment will be filed with this Clinical Trial Protocol.

Any amendment to the Clinical Trial Protocol requires written approval/favorable opinion by the Ethics Committee (IRB/IEC) prior to its implementation, unless there are overriding safety reasons. In some instances, an amendment may require a change to the Informed Consent Form. The Investigator must receive an IRB/IEC approval/favorable opinion concerning the revised Informed Consent Form prior to implementation of the change.

15 BIBLIOGRAPHIC REFERENCES

1. Albers, G.W., et al., *Antithrombotic therapy in atrial fibrillation.* Chest, 2001. **119**(1 Suppl): p. 194S-206S.
2. Goldstein, L.B., et al., *Primary prevention of ischemic stroke: A statement for healthcare professionals from the Stroke Council of the American Heart Association.* Stroke, 2001. **32**(1): p. 280-99.
3. Lamassa, M., et al., *Characteristics, outcome, and care of stroke associated with atrial fibrillation in Europe: data from a multicenter multinational hospital-based registry (The European Community Stroke Project).* Stroke, 2001. **32**(2): p. 392-8.
4. Wolf, P.A., R.D. Abbott, and W.B. Kannel, *Atrial fibrillation as an independent risk factor for stroke: the Framingham Study.* Stroke, 1991. **22**(8): p. 983-8.
5. Gage, B.F., et al., *Selecting patients with atrial fibrillation for anticoagulation: stroke risk stratification in patients taking aspirin.* Circulation, 2004. **110**(16): p. 2287-92.
6. *Stroke Prevention in Atrial Fibrillation Study. Final results.* Circulation, 1991. **84**(2): p. 527-39.
7. Al-Saady, N.M., O.A. Obel, and A.J. Camm, *Left atrial appendage: structure, function, and role in thromboembolism.* Heart, 1999. **82**(5): p. 547-54.
8. Blackshear, J.L. and J.A. Odell, *Appendage obliteration to reduce stroke in cardiac surgical patients with atrial fibrillation.* Ann Thorac Surg, 1996. **61**(2): p. 755-9.
9. Leung, D.Y., et al., *Prognostic implications of left atrial spontaneous echo contrast in nonvalvular atrial fibrillation.* J Am Coll Cardiol, 1994. **24**(3): p. 755-62.
10. Healey, J.S., et al., *Left Atrial Appendage Occlusion Study (LAAOS): results of a randomized controlled pilot study of left atrial appendage occlusion during coronary bypass surgery in patients at risk for stroke.* Am Heart J, 2005. **150**(2): p. 288-93.
11. Holmes, D.R., et al., *Percutaneous closure of the left atrial appendage versus warfarin therapy for prevention of stroke in patients with atrial fibrillation: a randomised non-inferiority trial.* Lancet, 2009. **374**(9689): p. 534-542.
12. Fuster, V., et al., *ACC/AHA/ESC 2006 guidelines for the management of patients with atrial fibrillation-executive summary: a report of the American College of Cardiology/American Heart Association Task Force on Practice Guidelines and the European Society of Cardiology Committee for Practice Guidelines (Writing Committee to Revise the 2001 Guidelines for the Management of Patients with Atrial Fibrillation).* Eur Heart J, 2006. **27**(16): p. 1979-2030.
13. Hart, R.G., L.A. Pearce, and M.I. Aguilar, *Meta-analysis: antithrombotic therapy to prevent stroke in patients who have nonvalvular atrial fibrillation.* Ann Intern Med, 2007. **146**(12): p. 857-67.
14. Hart, R.G., et al., *Antithrombotic therapy to prevent stroke in patients with atrial fibrillation: a meta-analysis.* Ann Intern Med, 1999. **131**(7): p. 492-501.
15. Gottlieb, L.K. and S. Salem-Schatz, *Anticoagulation in atrial fibrillation. Does efficacy in clinical trials translate into effectiveness in practice?* Arch Intern Med, 1994. **154**(17): p. 1945-53.
16. Samsa, G.P., et al., *Which approach to anticoagulation management is best? Illustration of an interactive mathematical model to support informed decision making.* J Thromb Thrombolysis, 2002. **14**(2): p. 103-11.
17. Lafata, J.E., et al., *The cost-effectiveness of different management strategies for patients on chronic warfarin therapy.* J Gen Intern Med, 2000. **15**(1): p. 31-7.
18. Reynolds, M.W., et al., *Warfarin anticoagulation and outcomes in patients with atrial fibrillation: a systematic review and metaanalysis.* Chest, 2004. **126**(6): p. 1938-45.
19. Ho, P.M., C.L. Bryson, and J.S. Rumsfeld, *Medication adherence: its importance in cardiovascular outcomes.* Circulation, 2009. **119**(23): p. 3028-35.
20. Jackevicius, C.A., M. Mamdani, and J.V. Tu, *Adherence with statin therapy in elderly patients with and without acute coronary syndromes.* JAMA : the journal of the American Medical Association, 2002. **288**(4): p. 462-7.
21. Haynes, R.B., H.P. McDonald, and A.X. Garg, *Helping patients follow prescribed treatment: clinical applications.* JAMA : the journal of the American Medical Association, 2002. **288**(22): p. 2880-3.
22. Baroletti, S. and H. Dell'Orfano, *Medication adherence in cardiovascular disease.* Circulation, 2010. **121**(12): p. 1455-8.

23. Cutler, D.M. and W. Everett, *Thinking outside the pillbox--medication adherence as a priority for health care reform.* The New England journal of medicine, 2010. **362**(17): p. 1553-5.
24. Oates, D.J. and M.K. Paasche-Orlow, *Health literacy: communication strategies to improve patient comprehension of cardiovascular health.* Circulation, 2009. **119**(7): p. 1049-51.
25. Gurwitz, J.H., et al., *Incidence and preventability of adverse drug events among older persons in the ambulatory setting.* JAMA : the journal of the American Medical Association, 2003. **289**(9): p. 1107-16.
26. Sokol, M.C., et al., *Impact of medication adherence on hospitalization risk and healthcare cost.* Medical care, 2005. **43**(6): p. 521-30.
27. Rasmussen, J.N., A. Chong, and D.A. Alter, *Relationship between adherence to evidence-based pharmacotherapy and long-term mortality after acute myocardial infarction.* JAMA : the journal of the American Medical Association, 2007. **297**(2): p. 177-86.
28. Parker, C.S., et al., *Adherence to warfarin assessed by electronic pill caps, clinician assessment, and patient reports: results from the IN-RANGE study.* Journal of general internal medicine, 2007. **22**(9): p. 1254-9.
29. Ho, P.M., et al., *Medication nonadherence is associated with a broad range of adverse outcomes in patients with coronary artery disease.* American heart journal, 2008. **155**(4): p. 772-9.
30. Gialamas, A., et al., *Does point-of-care testing lead to the same or better adherence to medication? A randomised controlled trial: the PoCT in General Practice Trial.* The Medical journal of Australia, 2009. **191**(9): p. 487-91.
31. Stephenson, B.J., et al., *The rational clinical examination. Is this patient taking the treatment as prescribed?* JAMA : the journal of the American Medical Association, 1993. **269**(21): p. 2779-81.
32. *Thromboembolic prophylaxis in 3575 hospitalized patients with atrial fibrillation. The Clinical Quality Improvement Network (CQIN) Investigators.* Can J Cardiol, 1998. **14**(5): p. 695-702.
33. Bungard, T.J., et al., *Adequacy of anticoagulation in patients with atrial fibrillation coming to a hospital.* Pharmacotherapy, 2000. **20**(9): p. 1060-5.
34. Caro, J.J., et al., *Anticoagulant prophylaxis against stroke in atrial fibrillation: effectiveness in actual practice.* CMAJ, 1999. **161**(5): p. 493-7.
35. Fang, M.C., et al., *National trends in antiarrhythmic and antithrombotic medication use in atrial fibrillation.* Arch Intern Med, 2004. **164**(1): p. 55-60.
36. Humphries, K.H., et al., *Population rates of hospitalization for atrial fibrillation/flutter in Canada.* Can J Cardiol, 2004. **20**(9): p. 869-76.
37. Reynolds, M.R., et al., *Patterns and predictors of warfarin use in patients with new-onset atrial fibrillation from the FRACTAL Registry.* Am J Cardiol, 2006. **97**(4): p. 538-43.
38. Smith, N.L., et al., *Temporal trends in the use of anticoagulants among older adults with atrial fibrillation.* Arch Intern Med, 1999. **159**(14): p. 1574-8.
39. Bungard, T.J., et al., *Why do patients with atrial fibrillation not receive warfarin?* Arch Intern Med, 2000. **160**(1): p. 41-6.
40. Choudhry, N.K., et al., *Warfarin prescribing in atrial fibrillation: the impact of physician, patient, and hospital characteristics.* Am J Med, 2006. **119**(7): p. 607-15.
41. Maisel, W.H., *Left atrial appendage occlusion--closure or just the beginning?* N Engl J Med, 2009. **360**(25): p. 2601-3.
42. Fuster, V., et al., *ACC/AHA/ESC 2006 Guidelines for the Management of Patients with Atrial Fibrillation: a report of the American College of Cardiology/American Heart Association Task Force on Practice Guidelines and the European Society of Cardiology Committee for Practice Guidelines (Writing Committee to Revise the 2001 Guidelines for the Management of Patients With Atrial Fibrillation): developed in collaboration with the European Heart Rhythm Association and the Heart Rhythm Society.* Circulation, 2006. **114**(7): p. e257-354.
43. Bonow, R.O., et al., *ACC/AHA 2006 guidelines for the management of patients with valvular heart disease: a report of the American College of Cardiology/American Heart Association Task Force on Practice Guidelines (writing committee to revise the 1998 Guidelines for the Management of Patients With Valvular Heart Disease): developed in collaboration with the Society of Cardiovascular Anesthesiologists: endorsed by the Society for Cardiovascular Angiography and Interventions and the Society of Thoracic Surgeons.* Circulation, 2006. **114**(5): p. e84-231.

44. Fountain, R.B., et al., *The PROTECT AF (WATCHMAN Left Atrial Appendage System for Embolic PROTECTion in Patients with Atrial Fibrillation) trial.* Am Heart J, 2006. **151**(5): p. 956-61.
45. Cox, J.L., N. Ad, and T. Palazzo, *Impact of the maze procedure on the stroke rate in patients with atrial fibrillation.* J Thorac Cardiovasc Surg, 1999. **118**(5): p. 833-40.
46. Tang, A.S., et al., *Cardiac-resynchronization therapy for mild-to-moderate heart failure.* The New England journal of medicine, 2010. **363**(25): p. 2385-95.
47. Jones, W.J., L.S. Williams, and J.F. Meschia, *Validating the Questionnaire for Verifying Stroke-Free Status (QVSFS) by neurological history and examination.* Stroke, 2001. **32**(10): p. 2232-6.
48. Ezekowitz, M.D., et al., *Rationale and design of RE-LY: randomized evaluation of long-term anticoagulant therapy, warfarin, compared with dabigatran.* Am Heart J, 2009. **157**(5): p. 805-10, 810 e1-2.
49. Connolly, S., et al., *Rationale and design of ACTIVE: the atrial fibrillation clopidogrel trial with irbesartan for prevention of vascular events.* Am Heart J, 2006. **151**(6): p. 1187-93.
50. Cochran, W.G., *Sampling techniques*. 3d ed. Wiley publication in applied statistics1977, New York: Wiley. 428 p.
51. Go, A.S., et al., *Warfarin use among ambulatory patients with nonvalvular atrial fibrillation: the anticoagulation and risk factors in atrial fibrillation (ATRIA) study.* Annals of internal medicine, 1999. **131**(12): p. 927-34.
52. Waldo, A.L., et al., *Hospitalized patients with atrial fibrillation and a high risk of stroke are not being provided with adequate anticoagulation.* Journal of the American College of Cardiology, 2005. **46**(9): p. 1729-36.
53. Nieuwlaat, R., et al., *Antithrombotic treatment in real-life atrial fibrillation patients: a report from the Euro Heart Survey on Atrial Fibrillation.* European heart journal, 2006. **27**(24): p. 3018-26.
54. Hylek, E.M., et al., *Translating the results of randomized trials into clinical practice: the challenge of warfarin candidacy among hospitalized elderly patients with atrial fibrillation.* Stroke; a journal of cerebral circulation, 2006. **37**(4): p. 1075-80.
55. Birman-Deych, E., et al., *Use and effectiveness of warfarin in Medicare beneficiaries with atrial fibrillation.* Stroke; a journal of cerebral circulation, 2006. **37**(4): p. 1070-4.
56. Glazer, N.L., et al., *Newly detected atrial fibrillation and compliance with antithrombotic guidelines.* Archives of internal medicine, 2007. **167**(3): p. 246-52.
57. Mercaldi, C.J., et al., *Cost efficiency of anticoagulation with warfarin to prevent stroke in medicare beneficiaries with nonvalvular atrial fibrillation.* Stroke; a journal of cerebral circulation, 2011. **42**(1): p. 112-8.

16 APPENDIX

1.0 Introduction

Strokes due to atrial fibrillation (AF) are a significant problem, responsible for one-sixth of all strokes in Canada. Current preventative measures may take the form elimination of AF itself, which is frequently ineffective at preventing all AF episodes or pharmacological antiplatelet / anticoagulant therapy, which is often limited by side effects inherent to the medication or issues with patient compliance. A third option which has garnered much attention recently is the occlusion or removal of the left atrial appendage (LAA) in order to prevent atrial thrombi formation. To date there have been no sufficiently powered randomized trials that have investigated this option, however small trials have shown promising results. The Left Atrial Appendage Occlusion Study III (LAAOS III) will be a large trial that explores the risks and benefits of LAA occlusion/removal in conjunction with usual medical care. Because of the implications this trial may have in addressing stroke prevention in patients with AF, an economic analysis will be important to assess the cost implications of this trial.

2.0 Design

The primary outcome is the first occurrence of stroke or systemic arterial embolism. The secondary outcomes are: total mortality, operative safety outcomes (chest tube output in in the first post-operative 24 hours; rate of post-operative re-exploration for bleeding; and 30-day mortality), readmission for heart failure, major bleed, and myocardial infarction. The primary clinical hypothesis is that patients who have had their LAA occluded/removed will benefit over those on usual care alone.

Because the method of LAA occlusion could have a significant impact on the cost implications of this intervention, analyses of different scenarios exploring these differences will be conducted. Our analysis will consider the following three situations:
1) The cost implications based on actual resource use in the LAAOS III trial (base case).
2) The cost implications if all surgical occlusions of the LAA were achieved with amputation or closure of the LAA using inexpensive surgical sutures.
3) The cost implications of using stapler closure or medical device like the ATRICURE clip to achieve occlusion of the LAA.

Although the cost of occlusion technique could vary, it is likely that LAA removal / occlusion together with usual care will be cost-saving over the study period.

2.1 Study horizon
The follow up period of the study will be 5 years, with an anticipated median follow-up period of 4 years.

2.2 Study perspective
The viewpoint of a third party payer will be used in this trial. In-hospital data pertaining to the occlusion of the LAA, perioperative transesophageal echocardiogram (TEE) use, strokes and systemic arterial embolisms and oral-anticoagulant use at discharge will be collected during the trial.

2.3 Discounting
Costs will be reported in Canadian dollars (2012). Because follow-up for each patient is longer than a year, a discounting rate of 3% will be used.

3.0 Unit costs

Large multinational trials involving many countries add a new level of complexity for health economists as the sample size is fragmented and distributed between countries with different health care systems. Thus any economic analysis of a multinational trial is invariably limited by the intrinsic design of the study. The inevitable problem of variation of resources consumed[i] and unit costs from one country to the other (inter-country variations) and also within a single country (intra-country variations) could be described as "system effect". This "system effect" limits the applicability of the analysis to any of the participating countries. The approach we used in HOPE[ii], CURE[iii,iv,v,vi] and ACTIVE-A[vii] was to aggregate results of resources consumption (events) from all patients in all countries and multiply them with the unit costs from specific countries of interest to calculate the total costs of the intervention and control arms. As the sample size in each country does not allow a country specific analysis, this approach is based on the assumption that there is no difference in resource utilization between these countries. This approach provides some answers but with 2 serious limitations: they are restricted to the countries of interest and these results are based on a fragment of the study population. This approach has been challenged recently and a new consensus on the best way to handle this problem within the constraints of a large multi-center international trial is starting to emerge in the literature[viii] and we will adhere to its general principles.

This study will ultimately recruit 3,500 patients from at least 9 countries (Canada, China, Germany, the United States, Italy, Spain, Australia, the Netherlands, and the Philippines). The issue of inter-country variation is enormous as patients are recruited from countries with different health care system such as those in this study. Given the current design of this grant, as we cannot increase the sample size to account for the variations in resource utilization between countries, we are proposing a systematic approach i.e. collecting relevant resources associated with clinical events of interest from the CRFs and collect all unit costs from <u>all</u> participating countries. Although this approach represents a significant endeavor, it is the simplest way to deal with the methodological problem we are facing and we have demonstrated the feasibility of this approach in the recently published economic analysis of the ONTARGET[ix] trial.

4.0 Development of unit costs

This analysis will not focus on capturing all costs and resources consumed during this trial. Instead we will limit our focus to the cost of surgical occlusion of the LAA, and the outcomes of stroke and systemic arterial embolism since these are the only anticipated differences in outcomes and procedures between the two groups. Perioperative TEE use will also be recorded. The cost of reoperations due to bleeding will also be included, as bleeding is a possible adverse event of this procedure. The cost of surgical occlusion of the LAA will be determined based on the technique chosen by the operating surgeon. Since all patients, regardless of their randomization, are undergoing a clinically indicated cardiac surgical procedure, only the cost of the LAA closure method will be taken into account. The additional time (5 minutes) required by the surgeon to surgically occlude the LAA is anticipated to be insignificant.

During the follow up period, this economic analysis will consider hospitalization due to systemic arterial embolism and the yearly cost of stroke. We feel that using an annual cost of stroke is a

better reflection of the actual economic implications of stroke rather than a cost that reflects just the cost of the resources consumed during the index hospitalization and have use this approach in our economic analysis of the ACTIVE-A trial as well[7]. Oral anti-coagulant use during the trial period will also be captured in our analysis.

4.1 Canada

Supplies costs for the occlusion of the LAA will be obtained from HHS. As no DRG system exists in Canada, we rely on a detailed case-costing system developed at the Hamilton Health Sciences for hospitalization events[12 x xi xii xiii xiv]. This allows us to determine with precision, for cardiac diseases, the unit cost per location (a day in CCU or ICU, step-down unit, and regular ward), pharmacy costs, radiology tests, nuclear medicine investigations, and other interventions. From this system we will have detailed costs for systemic arterial embolism, including periprocedural costs (holding area, angiography suite). Professional fees (Ontario Fee Schedule) are added. Ontario Drug Benefit program prices will be used to establish drug costs.

4.2 Other countries

The economic team will prepare a list of unit costs needed for the analysis. These costs include the cost of the same key variables mentioned above. The national coordinator (or a delegate) for each country will have the task of providing these costs. This can be accomplished relatively easily when a working collaboration with local hospital administrators is possible. Many investigators already have a set of unit costs available. We believe that we can develop unit costs in all countries as we are working with experienced investigators who have participated in similar trials before. We have recently completed a similar analysis in a large multinational study (44 countries) ONTARGET and see no difficulties in obtaining the costs applicable to this study. Missing cost data will be estimated by using regression analysis similar to the technique used by Reed and Schulman but using local cost data rather than DRG to perform the regression. The total cost per patient will be translated into a single currency (CAD or USD) by using PPP (power purchasing parity) ratios.

5.0 Health care utilization

Resources consumed in the occlusion of the LAA will be recorded. This will consist of the method of the occlusion and quantity of the occlusion device used. Oral anti-coagulant use will also be recorded. All resources consumed in the treatment of strokes or systemic arterial embolism during this study will be included in the analysis. Other health care resources unrelated to the study i.e. cancer, orthopedic surgery, etc. would be ignored unless a significant difference between randomization groups is detected. Resources utilization is divided into 5 categories: oral anti-coagulants, stroke, systemic arterial embolism, and occlusion devices. Each category (average cost per patient) will be individually analyzed and a total average cost per patient will be provided.

5.1 Oral anti-coagulants

All oral anti-coagulants consumed by patient out of hospital will be recorded and a unit cost will be attributed. We will use the generic cost when available; otherwise the brand name cost will be used.

6.0 Analysis plan

Unit costs will be applied to patient-level utilization data to arrive at a cost per patient, and the average cost within each treatment group will be calculated. Since the cost data will not be normally distributed, a bootstrap analysis will be used to calculate standard errors and 95% confidence intervals for the difference in average costs. The bias corrected and accelerated (BCa) method will be used to obtain confidence intervals for average costs. With patient-level data for

both clinical outcomes and costs available, a stochastic cost-effectiveness analysis will be performed. Comparisons between the two groups will be conducted using t-tests based on these estimates of standard error. We expect a very complete data set with the LAAOS III study. Nevertheless, some data could be missing. Patients with missing data will not be excluded from the analysis. Missing data will be replaced by the mean cost for the missing item (mean imputation).

7.0 Economic team

Dr. Andre Lamy, health economic scientist will be the project leader. He will be assisted by a health economic analyst (Wesley Tong). Statisticians from PHRI will also assist in the analysis. These statisticians have also helped in the economic analysis of HOPE, CURE, ONTARGET, ACTIVE-A, TIMACS, CORONARY and ORIGIN.

8.0 References

[i] Van de Werf F, Topol E, Lee KL, et al. Variations in patient management and outcomes for acute myocardial infarction in the United States and other countries: Results from the Gusto trial. Journal of the American Medical Association, 1995;273:1586-1591.

[ii] Lamy A, Yusuf S, Pogue J, Gafni A; Heart Outcomes Prevention Evaluation Investigators. Cost implications of the use of ramipril in high-risk patients based on the Heart Outcomes Prevention Evaluation (HOPE) study. Circulation. 2003 Feb 25;107(7):960-5.

[iii] Lamy A, Jonsson B, Weintraub WS, Zhao F, Chrolavicius S, Bakhai A, Culler S, Gafni A, Lindgren P, Mahoney E, Yusuf S; The CURE Economic Group (on behalf of the CURE Investigators). The cost-effectiveness of the use of clopidogrel in acute coronary syndromes in five countries based upon the CURE study. Eur J Cardiovasc Prev Rehabil. 2004 Dec;11(6):460-5.

[iv] Mahoney EM, Mehta S, Yuan Y, Jackson J, Chen R, Gabriel S, Lamy A, Culler S, Caro J, Yusuf S, Weintraub WS; CURE Study Investigators. Long-term cost-effectiveness of early and sustained clopidogrel therapy for up to 1 year in patients undergoing percutaneous coronary intervention after presenting with acute coronary syndromes without ST-segment elevation. Am Heart J. 2006; 151:219-227.

[v] Kolm P, Yong Y, Veledar E, Mehta SR, O'Brien JA, Weinstraub WS. Cost-effectiveness of clopidogrel in acute coronary syndromes in Canada: A long-term analysis based on the CURE trial. Can J Cardiol. 2007; 13:1037-1042.

[vi] Weintraub WS, Mahoney EM, Lamy A, Culler S, Yuan Y, Caro J, Gabriel S, Yusuf S; CURE Study Investigators. Long-term cost-effectiveness of clopidogrel given for up to one year in patients with acute coronary syndromes without ST-segment elevation. J Am Coll Cardiol. 2005; 45:838-845.

[vii] Lamy A, Tong W, Gao P, Chrolavicius S, Gafni A, Yusuf S, Connolly SJ. The Cost of Clopidogrel Use in Atrial Fibrillation in the ACTIVE-A Trial. Can J Cardiol. 2011; In Press. Epub ahead of print.

[viii] Reed SD, Anstrom KJ, Bakhai A, Briggs AH, Califf RM, Cohen DJ, Drummond MF, Glick HA, Gnanasakthy A, Hlatky MA, O'Brien BJ, Torti FM Jr, Tsiatis AA, Willan AR, Mark DB, Schulman KA. Conducting economic evaluations alongside multinational clinical trials: toward a research consensus. Am Heart J. 2005 Mar;149(3):434-43.

[ix] Lamy A, Wang X, Gao P, Tong W, Gafni A, Dans A, Avezum A, Ferreira R, Young J, Yusuf S, Teo K; ONTARGET Investigators. The cost implications of the use of telmisartan or ramipril in patients at high risk for vascular events: the ONTARGET study. J Med Econ. 2011; 14: 792-7.

[x] Lamy A, Yusuf S, Pogue J, Gafni A. Cost implications of the use of Ramipril in high-risk patients based on the Heart Outcomes Prevention Evaluation (HOPE Study) Circulation 2003 ;107 (7) .960-965

[xi] André Lamy, X. Wang, R. Kent, K, Smith, A. Gafni. Economic Evaluation of the MEDENOX trial: A Canadian Perspective. Canadian Respiratory Journal, Vol. 9(3), 169-77, May/June 2002

[xii] Andre Lamy, Susan Chrolavicius, Amiram Gafni, Salim Yusuf, Feng Zhao, Bengt Jonsson, Peter Lindgren, Ameet Bakhai, Marcus Flather, Keith Fox, Steve Culler, Elizabeth Mahoney, William Weintraub. The Cost-Effectiveness of the Use of Clopidogrel in Acute Coronary Syndromes based upon the CURE Study. American Heart Association Meeting November 2002, Chicago Ill.

[xiii] Smith KM, Lamy A, Arthur HM, Gafni A, Kent R. Outcomes and costs of coronary artery bypass grafting: comparison between octogenarians and septuagenarians at a tertiary care centre. CMAJ 2001;165:759-764.

[xiv] Ontario Case Cost Project. Ontario Case Cost Project (OCCP): Ontario Guide to Case Costing, Version 1.1, Ottawa, Ontario Case Cost Project; 1995.

LAAOS

Left Atrial Appendage Occlusion Study III

Protocol #: LAAOS III-2012
ClinicalTrials.gov ID: NCT01561651
EudraCT #: 2012-001478-27

WORKING PROTOCOL

Date: April 24, 2012 (version 1.0)
Revised Date (Amendment 1.0): June 14, 2012 (version 2.0)
Revised Date (Amendment 2.0): January 18, 2013 (version 3.0)
Revised Date (Amendment 3.0): November 18, 2013 (version 4.0)
Revised Date (Amendment 4.0): April 22, 2015 (version 5.0)

Study Coordinating Centre:	Population Health Research Institute<br>DBCVSRI, 237 Barton Street East<br>Hamilton, Ontario, L8L 2X2<br>Canada

Protocol Approval Signature

Dr. Richard Whitlock<br>Co-Principal Investigator<br>Population Health Research Institute	Name	2015-04-22<br>Date (yyyy-mm-dd)

This protocol is the confidential intellectual property of the Study Steering Committee and McMaster University and Hamilton Health Sciences (Population Health Research Institute). The use of any unpublished material presented in this document must be restricted to the recipient for the agreed purpose and must not be disclosed to unauthorized persons without the written consent of the LAAOS III Steering Committee.

NAMES AND ADDRESSES OF:

Co-Principal Investigator	Name:	Dr. Stuart Connolly
	Address:	Population Health Research Institute<br>237 Barton Street East, C3-123<br>Hamilton, ON L8L 2X2
	Tel:	905-527-4322 x 40317
	Fax:	905-297-2778
	Email:	Stuart.Connolly@phri.ca

Co-Principal Investigator	Name:	Dr. Richard Whitlock
	Address:	Population Health Research Institute<br>237 Barton Street East, C1-114<br>Hamilton, ON L8L 2X2
	Tel:	905-527-4322 x 40306
	Fax:	905-297-2778
	Email:	Richard.Whitlock@phri.ca

Project Manager	Name:	Ms. Shirley Pettit
	Address:	Population Health Research Institute<br>237 Barton Street East, C1-235<br>Hamilton, ON L8L 2X2
	Tel:	905-527-4322 x 40526
	Fax:	905-297-3779
	Email:	Shirley.Pettit@phri.ca

Research Coordinator	Name:	Ms. Kate Brady
	Address:	Population Health Research Institute<br>237 Barton Street East, C1-242<br>Hamilton, ON L8L 2X2
	Tel:	905-527-4322 x 41198
	Fax:	905-297-3779
	Email:	Kate.Brady@phri.ca

**LAAOS III Project Office Email:**	**LAAOSIII@phri.ca**

INVESTIGATOR AGREEMENT PAGE

I,____, Investigator, have examined this protocol

Left Atrial Appendage Occlusion Study III (LAAOS III)

and I have fully discussed the objectives of this trial and the contents of this protocol with the sponsor.

I agree to conduct the study according to this protocol and to comply with its requirements, subject to ethical and safety considerations.

I acknowledge that I am responsible for overall study conduct. I agree to personally conduct or supervise the described clinical study.

I agree to ensure that all associates, colleagues and employees assisting in the conduct of the study are informed about their obligations. Mechanisms are in place to ensure that site staff receives the appropriate information throughout the study.

Investigator Signature: ____

Date: ____

STUDY SYNOPSIS

Title	Left Atrial Appendage Occlusion Study III (LAAOS III)
Principal Investigators	Dr. Stuart Connolly and Dr. Richard Whitlock
Study Objective(s)	The primary objective is to examine the impact of LAA occlusion on the incidence of ischemic stroke* or transient ischemic attack with positive neuroimaging or systemic arterial embolism over the duration of follow-up in patients with atrial fibrillation undergoing cardiac surgery with the use of cardiopulmonary bypass.<br><br>The secondary objectives over duration of follow-up (unless otherwise specified) are:<br><br>1) To determine the incidence of all cause stroke or transient ischemic attack with positive neuroimaging or systemic arterial embolism.<br>2) To determine the incidence of ischemic stroke* or transient ischemic attack with positive neuroimaging or systemic arterial embolism or death.<br>3) To determine the incidence of ischemic stroke* or transient ischemic attack with positive neuroimaging or systemic arterial embolism > 30 days after surgery.<br>4) To determine the incidence of all cause death.<br><br>Safety objectives:<br><br>1) To determine the incidence of hospitalization for heart failure.<br>2) To determine post-operative safety outcomes<br>a. Chest tube output in the first post-operative 24 hours<br>b. Re-operation for bleeding within 48 hours post-surgery<br>c. 30-day mortality<br>3) To determine the incidence of major bleeding.<br>4) To determine the incidence of myocardial infarction.<br>* Ischemic stroke is defined as any stroke that is not documented as primary hemorrhagic.
Study Design	An international multicentre RCT of surgical left atrial appendage occlusion or no occlusion in a total of 4,700 patients undergoing cardiac surgery requiring cardiopulmonary bypass.
Study Population<br><br>Main selection criteria:	 <br><u>Inclusion Criteria</u><br>1) Age ≥ 18 years of age<br>2) Undergoing a clinically indicated cardiac surgical procedure with the use of cardiopulmonary bypass<br>3) A documented history of atrial fibrillation or atrial flutter<br>4) CHA_2DS_2-VASc score ≥2

Total number of subjects:<br>Expected number of centres:	5) Written informed consent<br><br><u>Exclusion Criteria</u><br>1) Patients undergoing any of the following procedures:<br>a. Off-pump cardiac surgery<br>b. Heart transplant<br>c. Complex congenital heart surgery<br>d. Sole indication for surgery is ventricular assist device insertion<br>e. Previous cardiac surgery (re-operation)<br>f. Mechanical valve implantation<br>2) Patients who have had a previous placement of a percutaneous LAA closure device<br><br>Total of 4,700 patients<br>Approximately 80-100 centres worldwide
Study Intervention	The intervention is occlusion of the LAA which is compared to no LAA occlusion. Occlusion must be performed using either amputation-and-closure (cut and sew), stapler device, an atrial appendage closure device approved by the applicable governing regulatory authority, or a double layer linear closure from within the atrium (mini thoracotomy cases if successful closure will be confirmed by TEE). Other LAA interventions proven to be efficacious may be proposed for use and approved by the Operations Committee on a case-by-case basis with such approval clearly documented. The use of simple purse string closure of the LAA is strictly prohibited. Intraoperative TEE is encouraged to determine successful closure of the appendage.
Evaluation Criteria	The primary outcome is the first occurrence of ischemic stroke* or transient ischemic attack with positive neuroimaging or systemic arterial embolism over the duration of follow-up.<br><br>The secondary outcomes over the duration of follow-up (unless otherwise specified) are:<br><br>1) All cause stroke or transient ischemic attack with positive neuroimaging or systemic arterial embolism<br>2) Composite of ischemic stroke* or transient ischemic attack with positive neuroimaging or systemic arterial embolism or death<br>3) Ischemic stroke* or transient ischemic attack with positive neuroimaging or systemic arterial embolism occurring > 30 days after surgery<br>4) All cause death

	Safety Outcomes:<br><br>1) Hospitalization for heart failure<br>2) Operative safety outcomes<br>a. Chest tube output in the first post-operative 24 hours<br>b. Re-operation for bleeding within the first 48 hours post-surgery<br>c. 30-day mortality<br>3) Major bleed<br>4) Myocardial infarction<br><br>* Ischemic stroke is defined as any stroke that is not documented as primary hemorrhagic.<br>† All components of composite outcomes will also be reported individually.
Statistical Considerations	The intention to treat principle, in which all participants will be included in their assigned treatment groups regardless of actual surgical procedure performed, will guide all analyses. A time to event analysis will be used to test the primary outcome variable. The primary outcome (stroke or systemic arterial embolism) will be presented using Kaplan-Meier survival curves and be compared between groups using a log rank test. The treatment effect as measured by the hazard ratio and 95% confidence interval and adjusted for other covariates will be derived by the Cox proportional hazards model. The secondary and other outcomes will be compared via a t-test, chi-square test, or non-parametric tests where appropriate.
Duration of Study Period (per subject)	Patients will be followed at hospital discharge, 30 days, one year and annually thereafter until the common study end date (to be determined at approximately 5 years after the first patient randomized). Interim telephone calls will be held at the 6-month intervals to maintain contact with the patients.

LIST OF ABBREVIATIONS

Abbreviation	Definition
ACTIVE	Atrial Fibrillation Clopidogrel Trial with Irbesartan for Prevention of Vascular Events
AF	Atrial fibrillation
AFFIRM	Atrial Fibrillation Follow-Up Investigation of Rhythm Management
CABG	Coronary artery bypass grafting
CCORT	Canadian Cardiovascular Outcomes Research Team
$CHADS_2$ Score	Congestive heart failure (1 point), Hypertension (1 point), Age >75 (2 points), Diabetes Mellitus (1 point), Stroke or TIA (2 points)
CHA_2DS_2-VASc Score	Congestive heart failure/LV dysfunction (1 point), Hypertension (1 point), Age ≥ 75 (2 points), Diabetes Mellitus (1 point), Stroke/TIA/ thromboembolism (2 points), Vascular disease (prior MI, PAD or aortic plaque) (1 point), Age 65-74 (1 point), Sex (1 point for female)
CK-MB	Creatine kinase myocardial b fraction
CNS	Central nervous system
CORONARY	CABG Off OR ON Pump RevAsculaRization StudY
CPB	Cardiopulmonary bypass
CRF	Case report form
DRG	Diagnosis-Related Group
DSMB	Data safety monitoring board
ECG	Electrocardiogram
ED	Endothelial dysfunction
FRACTAL	Fibrillation Registry Assessing Costs, Therapies, Adverse events and Lifestyle study
GCP	Good Clinical Practice
ICU	Intensive care unit
INR	International normalized ratio
IRB	Institutional Review Board
IWRS	Interactive web randomization system
LA	Left atrium
LAA	Left atrial appendage
LAAOS	Left atrial appendage occlusion study
LV	Left ventricle
LVD	Left ventricular dysfunction
LVEF	Left ventricular ejection fraction
MI	Myocardial infarction
OAC	Oral anticoagulant
PCI	Percutaneous coronary intervention
PHRI	Population Health Research Institute
PROTECT AF	Randomized Prospective Trial of Percutaneous LAA Closure vs Warfarin for Stroke Prevention in AF
QVAFS	Questionnaire Verifying Stroke Free Status
RBC	Red blood cells
RCT	Randomized controlled trial
REB	Research Ethics Board
RR	Relative risk
TEE	Transesophogeal Echocardiogram
TIA	Transient ischemic attack
VKA	Vitamin K Antagonist

CONTENTS

List of Abbreviations ... 7
1 Introduction and Rationale ... 10
1.1 Problem being addressed ... 10
1.2 Embolic stroke in atrial fibrillation and the left atrial appendage ... 11
1.3 Oral anticoagulation ... 11
1.4 Limitations of oral anticoagulation therapy (which LAA occlusion may mitigate) ... 12
1.5 Current evidence regarding LAA occlusion ... 14
1.6 Background summary ... 15
2 Study Objectives ... 15
2.1 Primary objectives ... 15
2.2 Secondary objectives ... 15
2.3 Safety objectives ... 15
3 Study Design ... 16
3.1 Type of study ... 16
3.2 Expected number of patients ... 16
3.3 Method of intervention allocation ... 16
3.4 Methods for protecting against bias ... 16
3.5 Duration of the study period for each subject ... 16
4 Study Population ... 17
4.1 Inclusion Criteria ... 17
4.2 Exclusion Criteria ... 17
5 Study Procedures ... 17
5.1 Intervention ... 17
5.2 Schedule of visits and observations ... 18
5.2.1 Study flowchart ... 18
5.2.2 Visit schedule ... 18
5.2.3 Baseline Data Collection ... 19
5.2.4 Cardiac Surgery to Hospital Discharge ... 19
5.2.5 Follow-up Visits ... 19
5.3 Selection procedures (entry procedures) ... 19
5.4 Encouraging compliance ... 19
5.5 Antithrombotic management ... 20
5.6 Study outcomes ... 20
5.6.1 Primary Outcome ... 20
5.6.2 Secondary Outcomes ... 20
5.6.3 Safety Outcomes ... 20
5.6.4 Definitions of Study Outcomes ... 21
5.7 Emergency Unblinding ... 22
6 Statistical Considerations ... 22
6.1 Analysis population ... 22
6.2 Statistical methods ... 22
6.3 Planned subgroup analyses ... 23
6.4 Data Safety Monitoring Board (DSMB) ... 23
6.5 Sample size calculation ... 23
7 Sub-Studies and Ancillary Studies ... 25
8 Study Organization ... 26
9 Ethical Standards ... 26

9.1 Ethical considerations 26
9.2 Informed consent 26
9.3 Ethics Review Committee 26
10 Study Monitoring 26
10.1 Responsibilities of the investigator(s) 26
10.2 Data collection on electronic case report forms (eCRFs) 27
11 Administrative Rules 27
11.1 Curriculum vitae 27
11.2 Confidentiality 27
11.3 Record retention in investigating centre(s) 27
12 Ownership of Data and Use of the Study Results 27
13 Publications 28
14 Protocol Amendments 28
15 Bibliographic References 29
16 Appendix 32

1 INTRODUCTION AND RATIONALE

1.1 Problem being addressed

Atrial fibrillation (AF) is an important cause of stroke, responsible for at least one sixth of all strokes in Canada. It is well established that most strokes in patients with AF are cardio-embolic, originating from the left atrial appendage (LAA). Three main approaches to stroke prevention in AF can be conceptualized: 1) Elimination of AF, itself, 2) Prevention of clot formation by medical therapy (either antiplatelet or anticoagulant) and 3) Physical elimination of the LAA, the site of clot formation. To date, elimination (or suppression) of AF has not been effective against stroke, probably because no AF therapy has been able to suppress all AF episodes. Antithrombotic medical therapy has been very effective, but is limited by potential for serious bleeding and by the general problem of continuity of treatment, namely non-prescription, non-compliance, sub-optimal anticoagulation control and treatment withdrawal. The third approach, occlusion or removal of the LAA, is a logical idea that has received considerable recent attention due to positive results from a small trial of device closures. Although the results of this small trial are encouraging, these results are not definitive. They do, however, provide the impetus for further research.

All three approaches to stroke prevention have very different mechanisms of action and it is likely that their effects are complementary and additive, especially those of antithrombotic medical therapy and LAA occlusion. Medical therapy is limited by under-prescription and interruptions (both scheduled and inadvertent). In all these circumstances, reduction of clot formation by removal of the LAA could provide continuous protection. LAA occlusion on the other hand cannot reduce thrombus formation at other sites (e.g., left atrium proper, left ventricle, aorta) which may coexist in AF. Reducing the risk of thrombus formation at these other sites is best managed with systemic antithrombotic therapy. Thus, removal of the LAA and systemic antithrombotic therapy are likely to act additively or synergistically.

The LAA is a blind sac attached to the left atrium, which is often narrow and trabeculated. Echocardiographic studies have shown that the LAA is the major site of thrombus formation in patients with AF; with >90% of atrial thrombi occurring in this appendage. A recent small randomized trial of device closure of the LAA in AF patients reported non-inferiority of LAA occlusion to warfarin for a composite of stroke, bleeding and death. These data provide reasonable proof of concept for LAA occlusion, but have failed to convince most practitioners and regulatory authorities as the trial was small, the procedure is complex, serious adverse events were common, and the non-inferiority margin was unusually wide. A definitive randomized trial of LAA occlusion is needed.

Cardiac surgery provides an excellent opportunity to remove the atrial appendage at very low risk. During most cardiac surgery procedures, the LAA is exposed and readily accessible; and LAA removal only takes a few extra minutes. AF is a major cause of stroke and is common in patients requiring cardiac surgery. A large trial to test if opportunistic surgical removal of the LAA at the time of other routine cardiac surgery can reduce stroke in patients with AF is a high priority for 2 reasons: 1) a positive trial will immediately change clinical surgical practice making LAA occlusion a standard part of cardiac surgery which in turn would lead to a large reduction in the stroke burden of patients undergoing cardiac surgery; and 2) it will for the first time provide conclusive evidence that LAA occlusion reduces stroke, greatly stimulating the agenda of further research in this promising area.

1.2 Embolic stroke in atrial fibrillation and the left atrial appendage

AF associated stroke is associated with worse outcomes than those occurring in the absence of AF.[4, 5] Clinical and diagnostic imaging evidence indicates that at least 70% of all strokes in patients with AF are cardio-embolic from the left atrium.[6] An overview of echocardiographic and autopsy studies of atrial thrombus location concluded that at least 90% of left atrial thrombi are found in the LAA.[8] The left atrial appendage has pulsatile flow in sinus rhythm; this disappears in AF resulting in greatly reduced appendage emptying. This stasis, together with increased atrial fibrosis typical of AF, and activation of blood coagulation underlie thrombus formation in AF (Virchow's triad). Removal or occlusion of the LAA removes a key component of this triad which may in turn reduce thrombus formation and embolic stroke in AF patients.[9] The atrial appendages however are also a main source of atrial natriuretic peptide which plays a role in salt and water homeostasis; although a small randomized study (n=77) suggested no ill effects of appendage removal.[10]

Currently no adequately powered randomized trial of LAA removal has been done. The PROTECT AF trial was reported last year. It evaluated the Watchman device which is designed to occlude the LAA by delivery of an occluding device over a trans-venous, trans-septal approach.[11] PROTECT AF investigators chose to compare device therapy to warfarin in an unblinded non-inferiority trial using a composite outcome that included bleeding, thrombotic and fatal outcomes. This trial claimed non-inferiority to warfarin but the due to the weak design (small size, unconventional primary outcome and wide non-inferiority margins) it has failed to lead to regulatory approval. An on-going study is enrolling patients but using the same design. This trial design has provided some proof of concept to the occlusion approach but will continue to be limited by the complexity of the non-inferiority design against effective active therapy (warfarin). Recent non-inferiority trials of new oral anticoagulants against warfarin have required enrolments of between 14,000 and 20,000 patients to demonstrate non-inferiority.

If a complex procedure is required to occlude the LAA, it may be most appropriate to do this to replace warfarin, but if the LAA occlusion can be performed at time of routine surgery with almost no risk, then considering that surgical and medical therapies are almost certain to be complementary, it makes most sense to evaluate surgical LAA occlusion as an adjunct to usual medical therapy. Not only does the proposed design of our study overcome the significant limitations and obstacles of an unblinded non-inferiority trial but it innovates in testing the value of combined surgical and medical therapy which has a strong rationale. LAA occlusion and antithrombotic therapy have completely different mechanisms; occlusion removes the anatomic location for most potential cardiac thrombi, while antithrombotic therapy reduces the tendency for thrombi formation. It is a strong hypothesis that the two approaches will be additive or synergistic against stroke. Even the most effective antithrombotic therapy needs to be taken once or twice every day over years (even decades) to be fully beneficial; a challenge even to the most compliant patient. LAA occlusion once adequately performed will never re-form and thus will provide un-interrupted protection against thrombus formation, and potentially stroke, for life.

1.3 Oral anticoagulation

Oral anticoagulant (OAC) therapy reduces the risk of stroke in AF and is recommended for stroke prevention in patients with AF who have risk factors for stroke.[12] A Cochrane meta-analyses that included twenty-nine trials and 28,044 patients [13, 14] reported that warfarin reduced the relative risk of stroke by 64% (95% CI, 49% to 74%) compared to no treatment and by 37% (95% CI, 23% to 48%) compared to aspirin. Aspirin is also effective, reducing the relative risk of stroke in AF by

20%. Anticoagulation is now recommended for all higher risk patients with AF. However there are still many patients who only receive antiplatelet therapy. Administrative database surveys indicate that only about two-thirds of patients who might benefit from anticoagulants actually receive one and discontinuation rates of warfarin approach 50% by 3 years.

New oral anticoagulants are being introduced which also reduce stroke in AF; the direct thrombin inhibitor, dabigatran, and the Factor Xa inhibitors rivaroxaban and apixaban. These agents have been evaluated in large clinical trials and have been shown to be non-inferior, and in some cases superior, to warfarin for stroke reduction; with similar or less bleeding. Dabigatran 110 mg, apixaban and rivaroxaban all showed very similar rates of ischemic stroke relative to warfarin, whereas dabigatran 150 mg showed a significant 25% relative risk reduction compared to warfarin. Both Factor Xa inhibitors and both dabigatran doses showed a large reduction in hemorrhagic strokes compared to warfarin. Major bleeding rates on all these agents, however, exceeded 3% per year, and minor bleeding rates were over 10% per year. Thus hemorrhage remains a significant limitation of both old and new oral anticoagulants. One advantage of the new agents is that they do not require monitoring which makes them easier to take than warfarin; but this paradoxically limits the physician's ability to ensure patient compliance.

1.4 Limitations of oral anticoagulation therapy (which LAA occlusion may mitigate)

There are many limitations to OAC therapy: 1) increased risk of bleeding; 2) need for monitoring of coagulation (INR) for warfarin; 3) patient non-compliance, a problem with all chronic medications (see next section below); 4) physician reluctance to prescribe especially to elderly patients; and 5) frequent need for therapy discontinuations for surgery, procedures and diagnostic tests.

Increased bleeding, both major and minor is inherent in all antithrombotic therapy. For example, in the recent RE-LY Trial, the annual rates of major bleeding were 2.7%, 3.1%, and 3.4% for dabigatran 110 mg BID, 150 mg BID and warfarin, respectively; and minor bleeding rates were 13%, 15% and 16% per year. Major bleeding is serious. In both ACTIVE and RE-LY trials, major bleeding increased the adjusted risk of death several fold compared to those without bleeding. One of the biggest problems with bleeding is that even minor bleeding may lead to discontinuation of antithrombotic therapy and exposure to stroke risk; a problem that would be mitigated by concomitant LAA occlusion.

The need for monitoring of warfarin therapy makes it very unattractive to patients and because warfarin is difficult to control, it is a major limitation of therapy. Keeping patients in the therapeutic range of the INR is achieved only about half to two thirds of the time even in clinical trials where patients and centres are selected.[15] In typical community practice, the time in therapeutic range falls to about 50% as demonstrated by a recent overview of studies.[16, 17] A low time in range is strongly associated with an increased risk of both stroke and bleeding.[18] Thus a concurrent therapy such as LAA occlusion that reduces stroke and is continuously effective is likely to be beneficial in patients receiving warfarin. LAA occlusion would theoretically provide protection to patients when their INR is non-therapeutic.

Patient non-compliance is a major limitation inherent to OAC therapy. In a major review of medication compliance for cardiovascular disease, Ho and colleagues estimated that 25–55% of patients do not take their chronic cardiac medications as prescribed.[19] Medication adherence for asymptomatic or chronic conditions is typically lower than that for acute or symptomatic conditions, and drops substantially after the initial months of therapy.[19-22] The reasons for this include patient-related factors (e.g., health illiteracy, forgetfulness, socio-economic barriers),

medication-related factors (e.g., cost, complexity of the regimen, side effects) and provider-related factors (e.g., a lack of coordinated care and follow-up).[22-25] Non-adherence is strongly skewed towards under- rather than over-dosing, and is associated with an increased risk of death, disability, hospitalization, and avoidable health care costs.[19, 26-29] A recent study of point of care testing in 53 Australian general practices is instructive. The study included patients who required OAC and only 43% of patients on anticoagulants reported consistent adherence to therapy during the study.[30] There is also substantial evidence that physicians under-estimate the degree of medication non-compliance even in patients who they 'know well'.[31] Compliance issues continue to be a problem with all medications and may be more of a problem with new anticoagulants than with warfarin, due to short half-lives and lack of need to regular monitoring. Clearly LAA occlusion could provide benefit to many patients on medical therapy who are sometimes non-compliant.

The under-use of anticoagulants is widely documented in virtually every country where this has been studied (Table 1).[32-38]. Many patients (up to half) are unsuitable for warfarin for a variety of reasons and some will remain unsuitable for the new anticoagulants. In the CCORT AF study, using prescription claims databases in Alberta, British Columbia and Ontario from 1997 to 2000, less than one-half of AF patients filled a prescription for warfarin within 90 days of discharge for an AF hospitalization.[36] After initiation of warfarin, discontinuation is very common. In one large administrative database registry from the United Kingdom, Gallagher et al reported warfarin discontinuation rates of 50% within a 4 year follow-up period (Figure 1). A very recent analysis of Ontario Drug Benefit claims data in 125,195 patients >65 years with atrial fibrillation who initiated warfarin therapy, found that almost one third (31.8%) discontinued warfarin within 1 year of initiation, and the median time to discontinuation was 2.9 years (Tara Gomes, University of Toronto, personal communication). The main limitation of warfarin is concern about bleeding and this often prevents its use in otherwise suitable patients.[39, 40] This suggests that even with the new anticoagulants, non-use and discontinuation of anticoagulants will be a problem; one that can be mitigated potentially by LAA occlusion.

Table 1. Use of Oral Anticoagulant Therapy to Prevent Stroke in AF: Results of Recent Surveys

Year	Study	Published Survey Population	Treated With Warfarin, % (Patient Status)
1999	ATRIA Study[51]	11 082 US patients large health maintenance organization without contraindications	60 (high-risk patients)
2005	NABOR Study[52]	945 US patients from teaching, community, and VA hospitals	55 (high-risk patients)
2006	Euro Heart Survey[53]	2706 outpatients in 35 European countries	64
2006	Hylek et al[54]	402 US patients, _65 years old, not on warfarin at admission to teaching hospital	51 (discharged on warfarin)
2006	Birman-Deych et al[55]	16 007 US Medicare patients	49
2007	Glazer et al[56]	437 newly detected AF patients at high risk of stroke	59%
2011	Mercaldi et al[57]	119 764 nonvavlular AF Medicare patients	58.5%

ATRIA indicates Anticoagulation and Risk Factors in Atrial Fibrillation; NABOR, National Anticoagulation Benchmark and Outcomes Report.

Figure 1

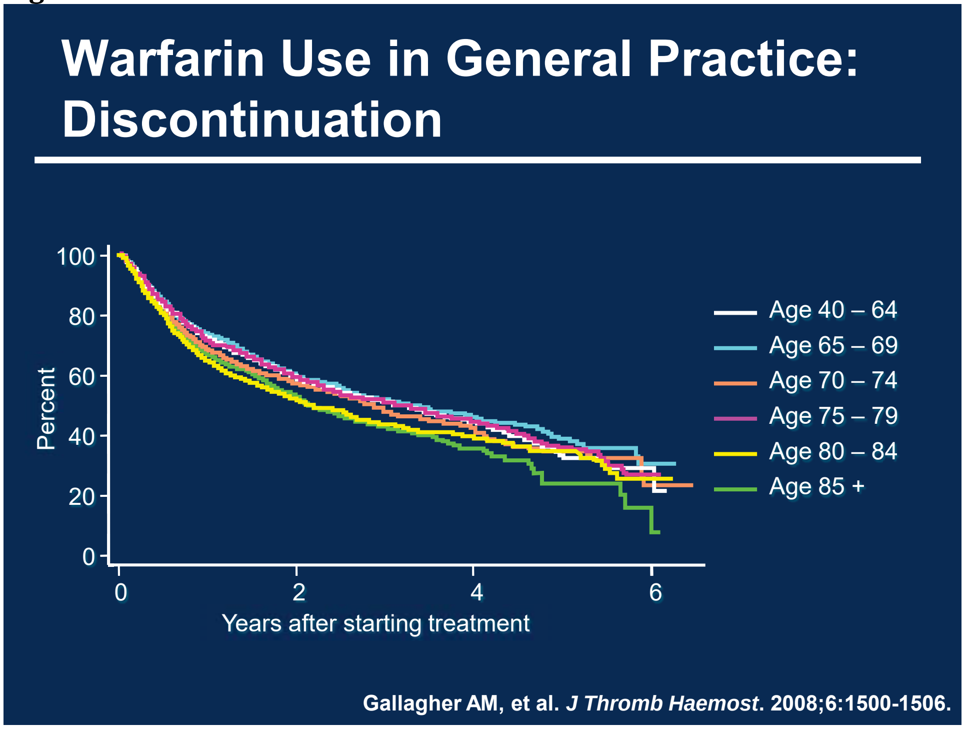

Interruption of anticoagulant therapy for surgery, procedures and diagnostic tests is very common in patients with AF. In the RE-LY Trial, dabigatran and warfarin patients were off of their anticoagulant study medication 13.6% of the time during the two years of follow up. Considering that these patients were being followed very closely by a dedicated study nurse and investigator who encouraged study medication compliance and re-initiation of therapy after a discontinuation, it is likely that rates of anticoagulation non-compliance are much greater in usual clinical practice; and LAA occlusion can potentially be very useful in this situation.

The fundamental limitations of OAC therapy and how LAA occlusion might mitigate these have been detailed. It is also important to recognize that LAA occlusion is not a panacea and that it might not be a suitable stand-alone therapy. AF is associated with a systemic hyper-coagulable state. Platelet function is enhanced with increased plasma levels of thromboglobulin and platelet factor 4. Systemic markers of activation of the coagulation cascade, such as thrombin-antithrombin II complex, D-dimers, fibrinogen, and prothrombin fragments 1 and 2, are also increased. Although most thrombi form in the left atrial appendage, some likely come from aortic plaque, the left ventricle and elsewhere. Thus a systemic antithrombotic therapy is likely a very good complement to a focused surgical intervention that targets only one source of embolism, albeit the most important one.

1.5 Current evidence regarding LAA occlusion

Prior to the publication of PROTECT AF, this literature was dominated by observational studies. It was upon these observational data that the America Heart Association based its recommendation to occlude the LAA in AF patients undergoing mitral valve surgery.[42, 43] In a retrospective study examining 205 patients post mitral valve surgery, the success rate of LAA closure when attempted approached 90%. Multivariate analysis demonstrated the absence of LAA ligation as an independent predictor of occurrence of an embolic event (OR 6.7, 95% CI 1.5-31.0). Results from

case series of ablation procedure patients are also often cited to support the amputation of the LAA.[44] The Maze procedure attempts to eliminate AF through a series of cuts in the right and left atria, suturing them closed, and excising both atrial appendages in a similar fashion. Cox et al. published a case series of 306 patients who underwent a "cut and sew" Maze procedure.[45] Rates of stroke were low but the majority of patients (n=162) were very low risk. Ultimately, these and several other small observational studies cannot provide the level of evidence needed to clearly answer this important question or substantially change clinical practice.

1.6 Background summary

In summary, it is hypothesized that LAA occlusion will reduce stroke and will benefit virtually all AF patients if it can be performed at very low-risk at the time of routine cardiac surgery. A positive result of an adequately powered and carefully executed clinical trial of surgical LAA occlusion versus no occlusion would also be the first unequivocal demonstration of the effectiveness of LAA occlusion. A positive trial would likely lead to almost universal adoption of this procedure at time of cardiac surgery, because it takes little time to perform and it is an easy procedure for any cardiac surgeon.

2 STUDY OBJECTIVES

2.1 Primary objectives

The primary objective is to examine the impact of LAA occlusion on the incidence of ischemic stroke* or transient ischemic attack with positive neuroimaging or systemic arterial embolism over the duration of follow-up in patients with atrial fibrillation undergoing cardiac surgery with the use of cardiopulmonary bypass.

2.2 Secondary objectives

The secondary objectives over duration of follow-up (unless otherwise specified) are:

1) To determine the incidence of all cause stroke or transient ischemic attack with positive neuroimaging or systemic arterial embolism.

2) To determine the incidence of ischemic stroke* or transient ischemic attack with positive neuroimaging or systemic arterial embolism or death.

3) To determine the incidence of ischemic stroke* or transient ischemic attack with positive neuroimaging or systemic arterial embolism > 30 days after surgery.

4) To determine the incidence of all cause death.

2.3 Safety objectives

1) To determine the incidence of hospitalization for heart failure.

2) To determine post-operative safety outcomes
   a. Chest tube output in the first post-operative 24 hours
   b. Re-operation for bleeding within 48 hours post-surgery

c. 30-day mortality

3) To determine the incidence of major bleeding.

4) To determine the incidence of myocardial infarction.

* Ischemic stroke is defined as any stroke that is not documented as primary hemorrhagic.

3 STUDY DESIGN

3.1 Type of study

An international, multi-centre, randomized blinded trial of surgical left atrial appendage occlusion in patients with atrial fibrillation/flutter who are undergoing cardiac surgery.

3.2 Expected number of patients

The total sample size for the study is 2,350 patients per group, for a total of 4,700 patients. Patients will be recruited from approximately 80-100 centres worldwide.

3.3 Method of intervention allocation

Eligible and consenting patients will be randomized via the central interactive web randomization system (IWRS) at the Population Health Research Institute. Each patient will be assigned in a blinded fashion to one of 2 groups (left atrial appendage occlusion or no left atrial appendage occlusion) according to a computer generated randomization list. Patients will be considered randomized when the intervention allocation has been provided through the IWRS. The confidential allocation email will be sent to the participating surgeon's email to maintain blinding of all others associated with the study.

3.4 Methods for protecting against bias

The patient and all study personnel, except the surgeon performing the cardiac surgery, will be blinded to the assigned allocation. This includes investigators (if other than the cardiac surgeon), study coordinators, other study team members, PHRI Project Office staff, and any other medical personnel involved in the patient's care. Although the surgeon will know the allocation, he/she must agree to not be involved in the ongoing antithrombotic management of the patient or in any of the post-operative data collection. Surgeons typically have little say in the on-going antithrombotic therapy of patients on whom they operate for reasons unrelated to the AF.

Blinding to intervention will be achieved as follows: the operative report and the dictated surgical report will only note that the patient has been enrolled in the LAAOS III trial and has been randomized to either left atrial appendage occlusion or no left atrial appendage occlusion. Data collection related to the intervention allocation and the details of the surgery performed will be maintained in a separate unblinded confidential database.

3.5 Duration of the study period for each subject

Although the study intervention occurs intra-operatively, all patients will be followed from the time of randomization until the final follow-up visit. Following randomization and baseline data

collection, visits will occur at hospital discharge, 30 days, one year and annually thereafter until the common study end date (to be determined at approximately 5 years after the first patient randomized). Interim telephone calls will be held at the 6-month intervals to maintain contact with the patients.

4 STUDY POPULATION

4.1 Inclusion Criteria

1) Age ≥ 18 years of age
2) Undergoing a clinically indicated cardiac surgical procedure with the use of cardiopulmonary bypass
3) A documented history of atrial fibrillation or atrial flutter
4) CHA_2DS_2-VASc score ≥ 2
5) Written informed consent

4.2 Exclusion Criteria

1) Patients undergoing any of the following procedures:
    a. Off-pump cardiac surgery
    b. Heart transplant
    c. Complex congenital heart surgery
    d. Sole indication for surgery is ventricular assist device insertion
    e. Previous cardiac surgery requiring opening of the pericardium
    f. Mechanical valve implantation
2) Patients who have had a previous placement of a percutaneous LAA closure device

5 STUDY PROCEDURES

5.1 Intervention

The intervention under investigation is surgical occlusion of the left atrial appendage which is compared to no left atrial appendage occlusion. The trial will permit the following techniques of LAA occlusion: 1) amputation of the LAA and closure (cut and sew); 2) stapler closure of the LAA; 3) use of an atrial appendage closure device that is approved by the applicable governing regulatory authority; or 4) double layer linear closure from within the atrium is acceptable for mini thoracotomy procedures if successful closure will be confirmed by TEE. Other LAA interventions proven to be efficacious may be proposed for use and approved by the Operations Committee on a case-by-case basis with such approval clearly documented. The use of simple purse string closure of the LAA is strictly prohibited. The preferred technique is amputation and closure as demonstrated by the video found at http://www.youtube.com/watch?v=aPoeoAhIjGw.']

Intraoperative TEE is encouraged to determine successful closure of the appendage. Successful occlusion is defined as TEE Doppler assessment demonstrating an absence of flow across the suture line and a stump of <1 cm. If the closure is not successful by this definition, additional maneuvers should be performed to rectify (e.g. additional sutures, additional staple line) as long as the surgeon feels that it is safe to do so. In patients with pre-operative appendage thrombus, the LAA must be opened to surgically remove the thrombus prior to the occlusion. An atrial ablation procedure can be performed; however, if randomized to non-occlusion, the LAA must not be occluded.

5.2 Schedule of visits and observations

5.2.1 Study flowchart

Eligibility Assessment
↓
Written Informed Consent
↓
Randomization
↓
Baseline Data Collection
↓
Cardiac Surgery with LAA Occlusion vs. No LAA Occlusion
↓
Hospital Discharge
↓
30-Day Visit
↓
Annual Visits until Final Follow-up Visit
Telephone Calls at 6 month Intervals
(Study End Date to be determined at approximately 5 years after first patient randomized)

5.2.2 Visit schedule

Table 2: Schedule of visits.

	In-hospital Phase			Follow-up Phase			
	Baseline (Pre-op)	Cardiac Surgery	Hospital Discharge (Post-op)	30-Day Clinic Visit†	6-Month, 1.5, 2.5, 3.5, 4.5-Year Telephone Visits[1]	1, 2, 3, 4, 5-Year Clinic Visits[1]†	Final Clinic Follow-up Visit
Eligibility assessment	✓						
Informed consent	✓						
Patient demography	✓						
Medical/surgical history	✓						
Concomitant medications	✓		✓	✓	✓	✓	✓
INR (if applicable)	✓		✓	✓	✓	✓	✓
Vital signs	✓						
Randomization	✓						
Heart rhythm	✓[2]		✓				
Details of surgical procedure		✓					
TEE (recommended)		✓					
Outcome events		✓	✓	✓	✓	✓	✓
Untoward medical events		✓	✓	✓	✓	✓	✓
QVSFS[3]					✓	✓	✓

[1] As required until common end date
[2] Pre-operative rhythm can be within 30 days prior to surgery (results from most recent ECG should be used)
[3] Questionnaire Verifying Stroke Free Status† Clinic visits may also occur by telephone

5.2.3 Baseline Data Collection

Key baseline characteristics such as demographics, medical history, cardiovascular anatomy, co-morbidities, concomitant medications as well as patient's eligibility criteria, will be collected on the Case Report Forms (CRFs) for all randomized patients. ECGs, serum creatinine, and other routine blood work should be obtained within 30 days prior to surgery.

5.2.4 Cardiac Surgery to Hospital Discharge

Research personnel will provide the operative details (except the blinded study allocation) including the type of cardiac surgery performed and other important information on the Operative CRF. Details of the ICU stay, including the dates of ICU admission and discharge, chest tube drainage, transfusions, and other clinical events will be recorded on the ICU CRFs. Patients will be assessed at hospital discharge for clinical events, concomitant and discharge medications as well as total hospital stay. Any primary or secondary outcomes occurring during the initial hospitalization will be noted on the hospital discharge CRFs and recorded on corresponding special CRFs.

The unblinded surgeon will be required to provide details regarding intervention compliance in a separate confidential database to maintain the study blind. Details of technique used (i.e. amputation and closure, stapler device, or atrial appendage closure device), and whether or not the occlusion was successful (as defined in Section 5.6.3) will be required for those patients who have undergone the LAA occlusion.

5.2.5 Follow-up Visits

Follow-up will occur at 30 days after randomization, at one year and annually thereafter until the final follow-up visit (the common study end date is expected to be determined at approximately 5 years after the first patient is randomized).Follow-up visits may occur as office visits or by telephone. Patients will be contacted at 6 month intervals by telephone to avoid patients lost to follow-up. Event CRFs should be completed as soon as the investigating site becomes aware of the event. Supporting documentation for each event is required and should be forwarded to the LAAOS III Project Office as soon as it is available to ensure timely adjudication of events.

The validated Questionnaire for Verifying Stroke Free Status (QVSFS) will be administered at the 6-month follow-up and at all visits thereafter until the Final Follow-up Visit.

5.3 Selection procedures (entry procedures)

After eligibility is confirmed, patients will receive complete information about the study both orally and in writing and will be approached to provide written informed consent. Patients should be randomized just prior to surgery if possible. During the randomization process, the intervention allocation will be provided to the surgeon in a confidential manner.

5.4 Encouraging compliance

Intervention non-compliance is not expected to be a major issue in this trial. The intervention is simple and occurs at a single time point and is controlled by the surgeon, outcomes are objective, and data forms are concise. Patients are generally followed by their surgeons/cardiologists for several years after their procedure. Surgeons are permitted to use their approved occlusion method of choice for patients randomized to this arm and therefore compliance with the allocated intervention is expected to be high. However, we do foresee circumstances where the surgeon may

elect not to occlude the appendage (e.g. unrecognized adhesions or other anatomical considerations). Pilot work suggests that the frequency of such an occurrence will be minimal (less than 1% of cases).

The 6 month interim telephone calls will assist in avoiding patients lost to follow-up by enhancing compliance to the visit schedule.

5.5 Antithrombotic management

Because the surgeon is aware of the occlusion intervention, he/she will leave the management of antithrombotic therapy for AF to the cardiologist, primary care physician or an anticoagulation clinic, ensuring the usual pattern of care for the management of the anticoagulation.

5.6 Study outcomes

5.6.1 Primary Outcome

The primary outcome is the first occurrence of ischemic stroke* or transient ischemic attack with positive neuroimaging or systemic arterial embolism over the duration of follow-up.

5.6.2 Secondary Outcomes

The secondary outcomes over the duration of follow-up (unless otherwise specified) are:

1) All cause stroke or transient ischemic attack with positive neuroimaging or systemic arterial embolism

2) Composite of ischemic stroke* or transient ischemic attack with positive neuroimaging or systemic arterial embolism or death

3) Ischemic stroke* or transient ischemic attack with positive neuroimaging or systemic arterial embolism occurring > 30 days after surgery

4) All cause death

5.6.3 Safety Outcomes

1) Hospitalization for heart failure

2) Operative safety outcomes
    a. Chest tube output in the first post-operative 24 hours
    b. Re-operation for bleeding within the first 48 hours post-surgery
    c. 30-day mortality

3) Major bleed

4) Myocardial infarction

* Ischemic stroke is defined as any stroke that is not documented as primary hemorrhagic.
† All components of composite outcomes will also be reported individually.

5.6.4 Definitions of Study Outcomes

Stroke
Diagnosis of stroke will require new focal neurological symptoms with rapid onset, lasting at least 24 hours. All strokes will be classified as definite ischemic, definite hemorrhagic or type uncertain. Transient ischemic attacks (TIAs) with positive neuroimaging will be upgraded to stroke during blinded outcome adjudication.

Transient Ischemic Attack (TIA)
An episode of a new focal neurologic deficit with rapid onset with signs or symptoms lasting <24 hours. TIAs with positive neuroimaging should be classified as a stroke, regardless of duration of symptoms.

Systemic Arterial Embolism
Systemic arterial embolism will be judged to occur where there is a clinical history consistent with an acute loss of blood flow to a peripheral artery (or arteries), which is supported by objective evidence of embolism.

Major bleed
Major bleeding **within the first 48 hours after surgery** is defined as per BARC Type 4: 1) Perioperative intracranial bleeding within 48 hours; and/or 2) Reoperation after closure of sternotomy for the purpose of controlling bleeding; and/or 3) Transfusion of ≥ 5 units whole blood or packed red blood cells within a 48 hour period (note: cell saver products are not counted); and/or 4) Chest tube output ≥ 2L within a 24 hour period.

Major bleeding **after 48 hours after surgery** is defined as per modified ISTH: 1) Fatal bleeding, and/or 2) Symptomatic bleeding in a critical area or organ, such as intracranial, intraspinal, intraocular, retroperitoneal, intra-articular or pericardial, or intramuscular with compartment syndrome, and/or 3) Bleeding causing a fall in hemoglobin level of 3.0 g/dL* or more, or leading to transfusion of two or more units of whole blood or red cells.
* corrected for transfusion (1 unit PRBC or 1 unit whole blood = 1 g/dL hemoglobin)

Hospitalization with Heart Failure
Re-hospitalization with an overnight stay or prolongation of an existing hospitalization due to heart failure which requires both clinical (i.e. any of the following signs: elevated jugular venous pressure, respiratory râles, crepitations, or presence of S3) and radiographic evidence (e.g. vascular redistribution, interstitial pulmonary edema, or frank alveolar pulmonary edema).

Efficacy of Occlusion Technique
Successful occlusion is defined as TEE Doppler assessment demonstrating an absence of flow across the suture line and a stump of <1 cm.

Sub-Classification of Death
All deaths will be classified as either cardiovascular or non-cardiovascular. Cardiovascular death is defined as any death with a cardiovascular cause and includes those deaths occurring within 30 days of a cardiovascular procedure (e.g. cardiac surgery, percutaneous transluminal coronary angioplasty), cardiac arrest, myocardial infarction, pulmonary embolus, stroke, hemorrhage, or deaths due to an unknown cause. Non-cardiovascular death is defined as deaths due to a clearly documented non-cardiovascular cause (e.g. trauma, infection, malignancy).

Myocardial Infarction
Perioperative MI (≤48 hours post-operatively) is defined as the presence of new Q-waves or a new left bundle branch block on electrocardiogram, combined with a biomarker (CK-MB or troponin) elevation of at least 5 times the upper reference limit. **Late MI** (>48 hours) is defined as ischemic symptoms, ECG changes consistent with myocardial infarction (new significant Q waves in two contiguous leads) or evolving ST-segment or T-wave changes in two contiguous leads signifying ischemia or new left bundle branch block (LBBB) or ST segment elevation and elevated cardiac markers (troponins or CK-MB) in the necrosis range. Myocardial injury occurring after a percutaneous coronary intervention (PCI) are included in the late perioperative MI group but are defined as elevation of cardiac markers at least 3 times upper limit of normal (ULN) within 24 hours of percutaneous coronary intervention (PCI) or characteristic evolution of new ECG changes.

Transfusion Requirements
Autologous blood, homologous processed red blood cells, whole blood, plasma, platelets, cryoprecipitate will be recorded for 24 hours after surgery.

24-Hour Chest Tube Output
Total chest tube output in the first 24 hours or until the tubes are removed, whichever comes earlier.

5.7 Emergency Unblinding

Legitimate but rare situations such as an unexpected serious adverse event may require unblinding. We recommend that all unblinding decisions be made jointly with the Project Office. If the local study investigator believes emergency unblinding is essential for the patient's management then it can be undertaken either through:

a) the unblinded surgeon; or
b) the PHRI toll-free unblinding help-line

The principal investigator should approve the request for unblinding wherever possible. Further details on unblinding will be provided in the Manual of Operations.

6 STATISTICAL CONSIDERATIONS

6.1 Analysis population

All randomized patients will be included in the analysis according to the allocated intervention at randomization). At least 2,350 patients will be equally allocated to the LAA occlusion arm or no LAA occlusion arm.

6.2 Statistical methods

The intention-to-treat principle, in which all participants will be included in their assigned treatment groups regardless of actual surgical procedure performed, will guide all analyses. A time-to-event analysis will be used to test the primary outcome variable. The primary outcome (stroke or systemic arterial embolism) will be presented using Kaplan-Meier survival curves and be compared between groups using a log rank test. The treatment effect as measured by the hazard ratio and 95% confidence interval will be derived by the Cox proportional hazards model. A p-value of <0.05 for the proportional hazards model will be considered as significant. The proportional

hazards assumption will also be tested by graphical means. This analysis will also be performed on the secondary outcomes of 'death and primary outcome'. All other secondary outcomes will be compared via a t-test, chi-square test, or non-parametric tests where appropriate. The primary outcome will be analyzed at a mean follow-up of 4 years.

6.3 Planned subgroup analyses

Additional Cox models will be used to evaluate interactions between treatment and subgroups of interest: antithrombotic used, amputation and closure technique versus other, successful occlusion by trial definition, $CHADS_2$ score, CHA_2DS_2-VASc score, LA dimension, and atrial ablation procedure. The primary analysis will be repeated secondarily as a per protocol analysis.

6.4 Data Safety Monitoring Board (DSMB)

The independent Data Safety Monitoring Board (DSMB) will ensure patient safety, receive and review interim analyses of efficacy data, provide feedback to the Steering Committee, and ensure the study follows the highest standards of ethics. Over the median follow-up of 4 years, we expect 385 primary outcome events. Two formal interim analyses will be undertaken when 50% (193 events) and 75% (289 events) of the expected events have occurred. Conservative statistical guidelines for data monitoring have been developed and will follow the modified Haybittle-Peto rule. For efficacy, reductions in events of ≥ 4 SD in the first interim analysis and ≥ 3 SD in the second will be used. To be considered significant these predefined boundaries will have to be exceeded in two consecutive analyses performed three or more months apart. Given the extremeness of the monitoring boundaries and the paucity of interim analyses, no adjustment will be made to the final p-value at the trial end. The DSMB in making a recommendation for early stopping will also consider the consistency of the secondary endpoints and any relevant external data. For safety, increase in the rates of the primary outcome of ≥ 3 SD (first look) and ≥ 2 SD (second look) will be used as a trigger for discussion of early stopping and reporting. A decision to continue or stop the trial would be based on a number of factors in addition to the statistical significance of the main results, including consistency of the pattern of the data over time and an assessment of net benefit-risk ratios. At any time during the study, if safety concerns arise the DSMB chairperson will assemble a meeting of the full committee. The DSMB will make their recommendations to the steering committee after considering all the available data and any external data from relevant studies.

6.5 Sample size calculation

This study will enroll 4,700 patients with a median follow-up of 4 years which will allow us to detect a 25% relative risk reduction (RRR) in the primary outcome with an expected control event rate of 2.5% per year. This trial would have 80% power, accounting for a 2%/year loss of patients due to competing death. This sample size is contingent on reasonable assumptions about the patient risk and the types of antithrombotic therapy that patients will receive during follow up. If the event rates are lower than expected, follow-up can be extended with this study design. Table 3 presents required sample size by effect size and power.

Table 3: Sample Size for Primary Outcome

Reduction in Hazard Ratio	Power		
	80%	85%	90%
25%	4700	5380	6300
30%	3170	3630	4240
35%	2260	2580	3020

Assumes proportional hazards model with control arm outcome rates of 2.5% per year, 2 year enrollment and total 5 year follow up period, 2% per year mortality/lost rate

The enrolment requirement of this trial depends primarily on two parameters: the expected event rate in the control arm and the treatment effect expected from LAA occlusion. We can estimate the event rate in the control arm of the study from the event rates on various antithrombotic treatments in recent trials (Table 4) if we have a good estimate of the $CHADS_2$ score. We have performed a registry of 1886 patients in which we observed that the mean $CHADS_2$ score of patients with AF coming to cardiac surgery was 2.3. (**Assumption 1)**.

Table 4: Annual rate of stroke or systemic embolism in current antithrombotic trials

Agent, Trial, (n of arm)	Stroke or systemic embolism (annual rate)
Aspirin, ACTIVE A (3782)	3.7%
Aspirin, AVERROES (2791)	3.5%
Aspirin and Plavix, ACTIVE A (3772)	2.8%
Apixaban, ARISTOTLE (9120)	1.3%
Apixaban, AVERROES (2808)	1.6%
Warfarin, ACTIVE W (3371)	1.5%
Warfarin, RELY (6022)	1.7%
Warfarin , ARISTOTLE (9081)	1.6%
Dabigatran 150 mg, RELY (6076)	1.1%
Dabigatran 110 mg, RELY (6015)	1.5%

Table 5 shows the expected treatment effects of LAA occlusion in different sub-groups of patients expected to be enrolled into the study. As can be appreciated from Table 5, to properly estimate the control event rate we also need to estimate the rate of use of different antithrombotic medications during follow up. Numerous surveys indicate that oral anticoagulants are used in only about 50% to 60% of high-risk patients with AF due to difficulties with control of the INR, bleeding risk, patient reluctance and physician behavior. The use of oral anticoagulants will tend to increase over the next few years as the new anticoagulants are introduced; however, there will still remain a substantial number of patients who either take aspirin or no therapy due to refusal to take an anticoagulant, difficulty with INR management, high cost of new anticoagulants, development of renal failure which increases the risk of anticoagulation. Therefore we estimate that the number of patient-years of follow on aspirin or no antithrombotic therapy will be 35% ±5%. **Assumption 2.** We have very good estimates of the rate of stroke or systemic embolism for these patients from ACTIVE A and AVERROES (3.7% per year on aspirin and 5.1% on no antithrombotic therapy)

Table 5: Expected event rates for primary outcome and relative risk reduction with LAA occlusion on top of usual care

Therapy Component of usual care	% patients years on therapy component	Control rate of primary outcome per year	Expected relative risk reduction with LAA occlusion	Treatment rate of primary outcome per year
No antithrombotic	5	5.1	63%	1.9
Aspirin	30	3.7	63%	1.4
Warfarin	45	1.7	25%	1.3
Novel anticoagulant	20	1.5	25%	1.1
Overall Usual Care	100	2.5	36%	1.6

Because of cost issues and familiarity, we estimate that warfarin and other Vitamin K antagonists will remain the most common oral anticoagulants used (45% of patient-years of follow up). There will be gradually increasing use of dabigatran and the Factor 10a inhibitors over the next 5 years. It is estimated that 20% of patient years of follow-up will be on dabigatran, rivaroxaban, or apixaban. We estimate, based on the recent large trials, that the primary event rate in control patients taking warfarin will be 1.7% per year and in those taking one of the new anticoagulants it will be 1.5% per year. Thus the overall annual event rate in the control arm without LAA occlusion is estimated at 2.5% per year.

This study is powered to have 80% power to detect a 25% relative risk reduction. A 25% treatment effect is reasonable because the PROTECT AF trial of device closure suggests that the effect of LAA occlusion is similar to that of warfarin, although the mechanism is obviously different and the effect of LAA occlusion will be additive to that of medical therapy. Table 5 shows that the largest effect will likely occur in those receiving no therapy or aspirin. The most recent data comparing an oral anticoagulant to aspirin in AF patients comes from AVERROES, where the reduction in ischemic stroke with apixaban compared to aspirin was 63% (HR = 0.37 (95% CI 0.25–0.55) p <0.001. For patients prescribed an oral anticoagulant, the treatment effect of LAA occlusion will be more modest but not trivial. The benefit of the surgical removal of the LAA will occur during warfarin therapy when patients are out of target therapeutic range (30-50% of time) and during therapy with any oral anticoagulants when there is non-compliance (which is very common); and interruptions for procedures and surgery, which are also common. In RE-LY, 25% of patients had at least one interruption of therapy for procedures. Overall in RE-LY patients were off study medication 14% of time. Based on these considerations a 25% relative reduction in stroke or systemic embolism with LAA occlusion in patients prescribed with oral anticoagulants is reasonable. The overall treatment effect of LAA occlusion on top of usual care is the blended total of these rates which is a 36% relative risk reduction. For the purpose of this trial, we plan to have sufficient statistical power to detect a reduction of 25%.

7 SUB-STUDIES AND ANCILLARY STUDIES

The economic analysis of LAAOS III will include the assessment of LAA occlusion costs, stroke, and systemic arterial embolism over the long-term follow-up. Our hypothesis is that left atrial appendage occlusion will be a dominant strategy i.e. clinically effective and cost-saving. Therefore, the economic analysis will focus on the cost of each surgical procedure in the participating countries (which can vary depending on the choice of occlusion method) and the cost of stroke in each group. The details of the economic analysis protocol are documented in the Appendix.

8 STUDY ORGANIZATION

The trial will be conducted internationally and coordinated at the Population Health Research Institute (PHRI) at McMaster University, Hamilton, Canada. The Steering Committee will be responsible for the design, execution, analysis, and reporting of the study, and will assign appropriate responsibilities to the other study committees. This committee will convene regularly (at least every 3 months) by telephone conference or meetings to address policy issues and to monitor study progress, execution and management. The Steering Committee will include the Principal Investigators and National Leaders from each participating country. The Operations Committee (a subset of the Steering Committee) will hold the primary responsibility for publication of the study results on behalf of the LAAOS III investigators. The Events Adjudication Committee will review all reported outcome events and are blinded to the intervention allocation when reviewing all events.

9 ETHICAL STANDARDS

9.1 Ethical considerations

The study will be conducted in accordance with Good Clinical Practice (GCP), all applicable subject privacy requirements, and the guiding principles of the declaration of Helsinki, including, but not limited to:

- Institutional Review Board (IRB)/Independent Ethics Committee (IEC) review and approval of study protocol and any subsequent amendments.
- Subject informed consent.

Written informed consent must be obtained from each subject prior to participation in the study.

9.2 Informed consent

It is the responsibility of the Investigator to obtain informed consent in compliance with national requirements from each subject prior to entering the trial or, where relevant, prior to evaluating the subject's suitability for the study.

The informed consent document used by the Investigator for obtaining subject's informed consent must be reviewed and approved by the PHRI prior to Ethics Review Committee or similar body (IRB, REB) submission.

9.3 Ethics Review Committee

The Investigator must submit this protocol to an Ethics Review Committee or a similar body (IRB, REB) and is required to forward a copy of the written approval/advice signed by the Chairman to the PHRI. On the approval/advice sheet, the trial name and protocol version, the study documents (protocol and informed consent material) and the date of the review should be clearly stated.

10 STUDY MONITORING

10.1 Responsibilities of the investigator(s)

The Investigator(s) undertake(s) to perform the study in accordance with Good Clinical Practice. The Investigator is required to ensure compliance with respect to the visit schedule and procedures

required by the protocol. The Investigator agrees to provide all information requested in the Case Report Forms in an accurate and timely manner according to instructions provided.

10.2 Data collection on electronic case report forms (eCRFs)

It is the responsibility of the Investigator to prepare and maintain adequate and accurate eCRFs which have been provided by the study to record all observations and other data pertinent to the clinical investigation. All eCRFs should be completed in their entirety and in a timely fashion.

11 ADMINISTRATIVE RULES

11.1 Curriculum vitae

An updated copy of the curriculum vitae for each Investigator and co-Investigator will be provided to the PHRI prior to the beginning of the study.

11.2 Confidentiality

All goods, materials, information (oral or written) and unpublished documentation provided to the Investigators, inclusive of this protocol, the patient Case Report Forms are the exclusive property of the Project Office. They may not be given or disclosed by the Investigator or by any person within their authority either in part or in totality to any unauthorized person without the prior written formal consent of the PHRI.

It is specified that the submission of this protocol and other necessary documentation to the Ethics Review Committee or a like body is expressly permitted, the Ethics Committee members having the same obligation of confidentiality.

The Investigator shall consider as confidential and shall take all necessary measures to ensure that there is no breach of confidentiality in respect of all information accumulated, acquired or deduced in the course of the trial, other than that information to be disclosed by law.

11.3 Record retention in investigating centre(s)

The Investigator must maintain all study records, patient files and other source data for the maximum period of time permitted by the hospital, institution or private practice and must be consistent with national regulatory requirements and ICH guidelines. It is recommended that the Investigator retain the study documents at least fifteen (15) years after the completion or discontinuation of the Clinical Trial. However, applicable regulatory requirements should be taken into account in the event that a longer period is required.

12 OWNERSHIP OF DATA AND USE OF THE STUDY RESULTS

The PHRI Project Office and the Steering Committee of the study have the ownership of all data and results collected during this study. In consequence, the PHRI Project Office reserves the right to use the data of the present study, either in the form of CRFs (or copies of these), or in the form of a report, with or without comments and with or without analysis, in order to submit them to the Health Authorities of any country. The Steering Committee has full rights to publication based on data from this study, without restriction.

13 PUBLICATIONS

All analyses for publication will be provided by the PHRI Project Office. The main responsibility for presentations and/or publications belongs to the Operations Committee. Publication of the main findings of this study will be made jointly in the name of all wholehearted collaborators. Other papers will be authored based on the contributions of the individuals to the overall study. All the trial participants (Investigators and committee members) make a prior delegation of responsibility for primary presentation and/or primary publication of the results to the Operations Committee. No other publication is allowed before the primary publication. Any presentation or publication by any trialist must mention the trial and has to be approved by the Steering Committee. Moreover, it is mandatory to make reference to the primary publication.

14 PROTOCOL AMENDMENTS

It is specified that the appendices attached to this protocol and referred to in the main text of this protocol, form an integral part of the protocol. The Investigator should not implement any deviation from, or changes of the Clinical Trial Protocol without agreement by the Sponsor and prior review and documented approval/favorable opinion from the IRB/IEC of an amendment, except where necessary to eliminate an immediate hazard(s) to Clinical Trial Patients, or when the change(s) involves only logistical or administrative aspects of the trial. Any changes agreed upon will be recorded in writing, the written amendment will be signed by the Investigator and by the Sponsor and the signed amendment will be filed with this Clinical Trial Protocol.

Any amendment to the Clinical Trial Protocol requires written approval/favorable opinion by the Ethics Committee (IRB/IEC) prior to its implementation, unless there are overriding safety reasons. In some instances, an amendment may require a change to the Informed Consent Form. The Investigator must receive an IRB/IEC approval/favorable opinion concerning the revised Informed Consent Form prior to implementation of the change.

15 Bibliographic References

1. Albers, G.W., et al., *Antithrombotic therapy in atrial fibrillation.* Chest, 2001. **119**(1 Suppl): p. 194S-206S.
2. Goldstein, L.B., et al., *Primary prevention of ischemic stroke: A statement for healthcare professionals from the Stroke Council of the American Heart Association.* Stroke, 2001. **32**(1): p. 280-99.
3. Lamassa, M., et al., *Characteristics, outcome, and care of stroke associated with atrial fibrillation in Europe: data from a multicenter multinational hospital-based registry (The European Community Stroke Project).* Stroke, 2001. **32**(2): p. 392-8.
4. Wolf, P.A., R.D. Abbott, and W.B. Kannel, *Atrial fibrillation as an independent risk factor for stroke: the Framingham Study.* Stroke, 1991. **22**(8): p. 983-8.
5. Gage, B.F., et al., *Selecting patients with atrial fibrillation for anticoagulation: stroke risk stratification in patients taking aspirin.* Circulation, 2004. **110**(16): p. 2287-92.
6. *Stroke Prevention in Atrial Fibrillation Study. Final results.* Circulation, 1991. **84**(2): p. 527-39.
7. Al-Saady, N.M., O.A. Obel, and A.J. Camm, *Left atrial appendage: structure, function, and role in thromboembolism.* Heart, 1999. **82**(5): p. 547-54.
8. Blackshear, J.L. and J.A. Odell, *Appendage obliteration to reduce stroke in cardiac surgical patients with atrial fibrillation.* Ann Thorac Surg, 1996. **61**(2): p. 755-9.
9. Leung, D.Y., et al., *Prognostic implications of left atrial spontaneous echo contrast in nonvalvular atrial fibrillation.* J Am Coll Cardiol, 1994. **24**(3): p. 755-62.
10. Healey, J.S., et al., *Left Atrial Appendage Occlusion Study (LAAOS): results of a randomized controlled pilot study of left atrial appendage occlusion during coronary bypass surgery in patients at risk for stroke.* Am Heart J, 2005. **150**(2): p. 288-93.
11. Holmes, D.R., et al., *Percutaneous closure of the left atrial appendage versus warfarin therapy for prevention of stroke in patients with atrial fibrillation: a randomised non-inferiority trial.* Lancet, 2009. **374**(9689): p. 534-542.
12. Fuster, V., et al., *ACC/AHA/ESC 2006 guidelines for the management of patients with atrial fibrillation-executive summary: a report of the American College of Cardiology/American Heart Association Task Force on Practice Guidelines and the European Society of Cardiology Committee for Practice Guidelines (Writing Committee to Revise the 2001 Guidelines for the Management of Patients with Atrial Fibrillation).* Eur Heart J, 2006. **27**(16): p. 1979-2030.
13. Hart, R.G., L.A. Pearce, and M.I. Aguilar, *Meta-analysis: antithrombotic therapy to prevent stroke in patients who have nonvalvular atrial fibrillation.* Ann Intern Med, 2007. **146**(12): p. 857-67.
14. Hart, R.G., et al., *Antithrombotic therapy to prevent stroke in patients with atrial fibrillation: a meta-analysis.* Ann Intern Med, 1999. **131**(7): p. 492-501.
15. Gottlieb, L.K. and S. Salem-Schatz, *Anticoagulation in atrial fibrillation. Does efficacy in clinical trials translate into effectiveness in practice?* Arch Intern Med, 1994. **154**(17): p. 1945-53.
16. Samsa, G.P., et al., *Which approach to anticoagulation management is best? Illustration of an interactive mathematical model to support informed decision making.* J Thromb Thrombolysis, 2002. **14**(2): p. 103-11.
17. Lafata, J.E., et al., *The cost-effectiveness of different management strategies for patients on chronic warfarin therapy.* J Gen Intern Med, 2000. **15**(1): p. 31-7.
18. Reynolds, M.W., et al., *Warfarin anticoagulation and outcomes in patients with atrial fibrillation: a systematic review and metaanalysis.* Chest, 2004. **126**(6): p. 1938-45.
19. Ho, P.M., C.L. Bryson, and J.S. Rumsfeld, *Medication adherence: its importance in cardiovascular outcomes.* Circulation, 2009. **119**(23): p. 3028-35.
20. Jackevicius, C.A., M. Mamdani, and J.V. Tu, *Adherence with statin therapy in elderly patients with and without acute coronary syndromes.* JAMA : the journal of the American Medical Association, 2002. **288**(4): p. 462-7.
21. Haynes, R.B., H.P. McDonald, and A.X. Garg, *Helping patients follow prescribed treatment: clinical applications.* JAMA : the journal of the American Medical Association, 2002. **288**(22): p. 2880-3.
22. Baroletti, S. and H. Dell'Orfano, *Medication adherence in cardiovascular disease.* Circulation, 2010. **121**(12): p. 1455-8.

23. Cutler, D.M. and W. Everett, *Thinking outside the pillbox--medication adherence as a priority for health care reform.* The New England journal of medicine, 2010. **362**(17): p. 1553-5.
24. Oates, D.J. and M.K. Paasche-Orlow, *Health literacy: communication strategies to improve patient comprehension of cardiovascular health.* Circulation, 2009. **119**(7): p. 1049-51.
25. Gurwitz, J.H., et al., *Incidence and preventability of adverse drug events among older persons in the ambulatory setting.* JAMA : the journal of the American Medical Association, 2003. **289**(9): p. 1107-16.
26. Sokol, M.C., et al., *Impact of medication adherence on hospitalization risk and healthcare cost.* Medical care, 2005. **43**(6): p. 521-30.
27. Rasmussen, J.N., A. Chong, and D.A. Alter, *Relationship between adherence to evidence-based pharmacotherapy and long-term mortality after acute myocardial infarction.* JAMA : the journal of the American Medical Association, 2007. **297**(2): p. 177-86.
28. Parker, C.S., et al., *Adherence to warfarin assessed by electronic pill caps, clinician assessment, and patient reports: results from the IN-RANGE study.* Journal of general internal medicine, 2007. **22**(9): p. 1254-9.
29. Ho, P.M., et al., *Medication nonadherence is associated with a broad range of adverse outcomes in patients with coronary artery disease.* American heart journal, 2008. **155**(4): p. 772-9.
30. Gialamas, A., et al., *Does point-of-care testing lead to the same or better adherence to medication? A randomised controlled trial: the PoCT in General Practice Trial.* The Medical journal of Australia, 2009. **191**(9): p. 487-91.
31. Stephenson, B.J., et al., *The rational clinical examination. Is this patient taking the treatment as prescribed?* JAMA : the journal of the American Medical Association, 1993. **269**(21): p. 2779-81.
32. *Thromboembolic prophylaxis in 3575 hospitalized patients with atrial fibrillation. The Clinical Quality Improvement Network (CQIN) Investigators.* Can J Cardiol, 1998. **14**(5): p. 695-702.
33. Bungard, T.J., et al., *Adequacy of anticoagulation in patients with atrial fibrillation coming to a hospital.* Pharmacotherapy, 2000. **20**(9): p. 1060-5.
34. Caro, J.J., et al., *Anticoagulant prophylaxis against stroke in atrial fibrillation: effectiveness in actual practice.* CMAJ, 1999. **161**(5): p. 493-7.
35. Fang, M.C., et al., *National trends in antiarrhythmic and antithrombotic medication use in atrial fibrillation.* Arch Intern Med, 2004. **164**(1): p. 55-60.
36. Humphries, K.H., et al., *Population rates of hospitalization for atrial fibrillation/flutter in Canada.* Can J Cardiol, 2004. **20**(9): p. 869-76.
37. Reynolds, M.R., et al., *Patterns and predictors of warfarin use in patients with new-onset atrial fibrillation from the FRACTAL Registry.* Am J Cardiol, 2006. **97**(4): p. 538-43.
38. Smith, N.L., et al., *Temporal trends in the use of anticoagulants among older adults with atrial fibrillation.* Arch Intern Med, 1999. **159**(14): p. 1574-8.
39. Bungard, T.J., et al., *Why do patients with atrial fibrillation not receive warfarin?* Arch Intern Med, 2000. **160**(1): p. 41-6.
40. Choudhry, N.K., et al., *Warfarin prescribing in atrial fibrillation: the impact of physician, patient, and hospital characteristics.* Am J Med, 2006. **119**(7): p. 607-15.
41. Maisel, W.H., *Left atrial appendage occlusion--closure or just the beginning?* N Engl J Med, 2009. **360**(25): p. 2601-3.
42. Fuster, V., et al., *ACC/AHA/ESC 2006 Guidelines for the Management of Patients with Atrial Fibrillation: a report of the American College of Cardiology/American Heart Association Task Force on Practice Guidelines and the European Society of Cardiology Committee for Practice Guidelines (Writing Committee to Revise the 2001 Guidelines for the Management of Patients With Atrial Fibrillation): developed in collaboration with the European Heart Rhythm Association and the Heart Rhythm Society.* Circulation, 2006. **114**(7): p. e257-354.
43. Bonow, R.O., et al., *ACC/AHA 2006 guidelines for the management of patients with valvular heart disease: a report of the American College of Cardiology/American Heart Association Task Force on Practice Guidelines (writing committee to revise the 1998 Guidelines for the Management of Patients With Valvular Heart Disease): developed in collaboration with the Society of Cardiovascular Anesthesiologists: endorsed by the Society for Cardiovascular Angiography and Interventions and the Society of Thoracic Surgeons.* Circulation, 2006. **114**(5): p. e84-231.

44. Fountain, R.B., et al., *The PROTECT AF (WATCHMAN Left Atrial Appendage System for Embolic PROTECTion in Patients with Atrial Fibrillation) trial.* Am Heart J, 2006. **151**(5): p. 956-61.
45. Cox, J.L., N. Ad, and T. Palazzo, *Impact of the maze procedure on the stroke rate in patients with atrial fibrillation.* J Thorac Cardiovasc Surg, 1999. **118**(5): p. 833-40.
46. Tang, A.S., et al., *Cardiac-resynchronization therapy for mild-to-moderate heart failure.* The New England journal of medicine, 2010. **363**(25): p. 2385-95.
47. Jones, W.J., L.S. Williams, and J.F. Meschia, *Validating the Questionnaire for Verifying Stroke-Free Status (QVSFS) by neurological history and examination.* Stroke, 2001. **32**(10): p. 2232-6.
48. Ezekowitz, M.D., et al., *Rationale and design of RE-LY: randomized evaluation of long-term anticoagulant therapy, warfarin, compared with dabigatran.* Am Heart J, 2009. **157**(5): p. 805-10, 810 e1-2.
49. Connolly, S., et al., *Rationale and design of ACTIVE: the atrial fibrillation clopidogrel trial with irbesartan for prevention of vascular events.* Am Heart J, 2006. **151**(6): p. 1187-93.
50. Cochran, W.G., *Sampling techniques*. 3d ed. Wiley publication in applied statistics1977, New York: Wiley. 428 p.
51. Go, A.S., et al., *Warfarin use among ambulatory patients with nonvalvular atrial fibrillation: the anticoagulation and risk factors in atrial fibrillation (ATRIA) study.* Annals of internal medicine, 1999. **131**(12): p. 927-34.
52. Waldo, A.L., et al., *Hospitalized patients with atrial fibrillation and a high risk of stroke are not being provided with adequate anticoagulation.* Journal of the American College of Cardiology, 2005. **46**(9): p. 1729-36.
53. Nieuwlaat, R., et al., *Antithrombotic treatment in real-life atrial fibrillation patients: a report from the Euro Heart Survey on Atrial Fibrillation.* European heart journal, 2006. **27**(24): p. 3018-26.
54. Hylek, E.M., et al., *Translating the results of randomized trials into clinical practice: the challenge of warfarin candidacy among hospitalized elderly patients with atrial fibrillation.* Stroke; a journal of cerebral circulation, 2006. **37**(4): p. 1075-80.
55. Birman-Deych, E., et al., *Use and effectiveness of warfarin in Medicare beneficiaries with atrial fibrillation.* Stroke; a journal of cerebral circulation, 2006. **37**(4): p. 1070-4.
56. Glazer, N.L., et al., *Newly detected atrial fibrillation and compliance with antithrombotic guidelines.* Archives of internal medicine, 2007. **167**(3): p. 246-52.
57. Mercaldi, C.J., et al., *Cost efficiency of anticoagulation with warfarin to prevent stroke in medicare beneficiaries with nonvalvular atrial fibrillation.* Stroke; a journal of cerebral circulation, 2011. **42**(1): p. 112-8.

16 APPENDIX

1.0 Introduction

Strokes due to atrial fibrillation (AF) are a significant problem, responsible for one-sixth of all strokes in Canada. Current preventative measures may take the form elimination of AF itself, which is frequently ineffective at preventing all AF episodes or pharmacological antiplatelet / anticoagulant therapy, which is often limited by side effects inherent to the medication or issues with patient compliance. A third option which has garnered much attention recently is the occlusion or removal of the left atrial appendage (LAA) in order to prevent atrial thrombi formation. To date there have been no sufficiently powered randomized trials that have investigated this option, however small trials have shown promising results. The Left Atrial Appendage Occlusion Study III (LAAOS III) will be a large trial that explores the risks and benefits of LAA occlusion/removal in conjunction with usual medical care. Because of the implications this trial may have in addressing stroke prevention in patients with AF, an economic analysis will be important to assess the cost implications of this trial.

2.0 Design

The primary outcome is the first occurrence of stroke or systemic arterial embolism. The secondary outcomes are: total mortality, operative safety outcomes (chest tube output in in the first post-operative 24 hours; rate of post-operative re-exploration for bleeding; and 30-day mortality), re-hospitalization for heart failure, major bleed, and myocardial infarction. The primary clinical hypothesis is that patients who have had their LAA occluded/removed will benefit over those on usual care alone.

Because the method of LAA occlusion could have a significant impact on the cost implications of this intervention, analyses of different scenarios exploring these differences will be conducted. Our analysis will consider the following three situations:

1) The cost implications based on actual resource use in the LAAOS III trial (base case).
2) The cost implications of all surgical occlusions of the LAA were achieved with amputation of the LAA using inexpensive surgical sutures.
3) The cost implications of using stapler closure to achieve occlusion of the LAA.

Although the cost of occlusion technique could vary, it is likely that LAA removal / occlusion together with usual care will be cost-saving over the study period.

<u>2.1 Study horizon</u>

The follow up period of the study will be 5 years, with an anticipated median follow-up period of 4 years.

<u>2.2 Study perspective</u>

The viewpoint of a third party payer will be used in this trial. In-hospital data pertaining to the occlusion of the LAA, perioperative transesophageal echocardiogram (TEE) use, strokes and systemic arterial embolisms and oral-anticoagulant use at discharge will be collected during the trial.

2.3 Discounting

Costs will be reported in Canadian dollars (2012). Because follow-up for each patient is longer than a year, a discounting rate of 3% will be used.

3.0 Unit costs

Large multinational trials involving many countries add a new level of complexity for health economists as the sample size is fragmented and distributed between countries with different health care systems. Thus any economic analysis of a multinational trial is invariably limited by the intrinsic design of the study. The inevitable problem of variation of resources consumed[i] and unit costs from one country to the other (inter-country variations) and also within a single country (intra-country variations) could be described as "system effect". This "system effect" limits the applicability of the analysis to any of the participating countries. The approach we used in HOPE[ii], CURE[iii][iv][v][vi] and ACTIVE-A[vii] was to aggregate results of resources consumption (events) from all patients in all countries and multiply them with the unit costs from specific countries of interest to calculate the total costs of the intervention and control arms. As the sample size in each country does not allow a country specific analysis, this approach is based on the assumption that there is no difference in resource utilization between these countries. This approach provides some answers but with 2 serious limitations: they are restricted to the countries of interest and these results are based on a fragment of the study population. This approach has been challenged recently and a new consensus on the best way to handle this problem within the constraints of a large multi-center international trial is starting to emerge in the literature[viii] and we will adhere to its general principles.

This study will ultimately recruit 4,700 patients from at least 9 countries (Canada, China, Germany, the United States, Italy, Spain, Australia, the Netherlands, and the Philippines). The issue of inter-country variation is enormous as patients are recruited from countries with different health care system such as those in this study. Given the current design of this grant, as we cannot increase the sample size to account for the variations in resource utilization between countries, we are proposing a systematic approach i.e. collecting relevant resources associated with clinical events of interest from the CRFs and collect all unit costs from <u>all</u> participating countries. Although this approach represents a significant endeavor, it is the simplest way to deal with the methodological problem we are facing and we have demonstrated the feasibility of this approach in the recently published economic analysis of the ONTARGET[ix] trial.

4.0 Development of unit costs

This analysis will not focus on capturing all costs and resources consumed during this trial. Instead we will limit our focus to the cost of surgical occlusion of the LAA, and the outcomes of stroke and systemic arterial embolism since these are the only anticipated differences in outcomes and procedures between the two groups. Perioperative TEE use will also be recorded. The cost of reoperations due to bleeding will also be included, as bleeding is a possible adverse event of this procedure. The cost of surgical occlusion of the LAA will be determined based on the technique chosen by the operating surgeon. Since all patients, regardless of their randomization, are undergoing a clinically indicated cardiac surgical procedure, only the cost of the LAA closure method will be taken into account. The additional time (5 minutes) required by the surgeon to surgically occlude the LAA is anticipated to be insignificant.

During the follow up period, this economic analysis will consider hospitalization due to systemic arterial embolism and the yearly cost of stroke. We feel that using an annual cost of stroke is a better reflection of the actual economic implications of stroke rather than a cost that reflects just the cost of the resources consumed during the index hospitalization and have use this approach in our economic analysis of the ACTIVE-A trial as well[7]. Oral anti-coagulant use during the trial period will also be captured in our analysis.

4.1 Canada

Supplies costs for the occlusion of the LAA will be obtained from Hamilton Health Sciences (HHS). As no Diagnosis-Related Group (DRG) system exists in Canada, we rely on a detailed case-costing system developed at HHS for hospitalization events[12 x xi xii xiii xiv]. This allows us to determine with precision, for cardiac diseases, the unit cost per location (a day in CCU or ICU, step-down unit, and regular ward), pharmacy costs, radiology tests, nuclear medicine investigations, and other interventions. From this system we will have detailed costs for systemic arterial embolism, including periprocedural costs (holding area, angiography suite). Professional fees (Ontario Fee Schedule) are added. Ontario Drug Benefit program prices will be used to establish drug costs.

4.2 Other countries

The economic team will prepare a list of unit costs needed for the analysis. These costs include the cost of the same key variables mentioned above. The national coordinator (or a delegate) for each country will have the task of providing these costs. This can be accomplished relatively easily when a working collaboration with local hospital administrators is possible. Many investigators already have a set of unit costs available. We believe that we can develop unit costs in all countries as we are working with experienced investigators who have participated in similar trials before. We have recently completed a similar analysis in a large multinational study (44 countries) ONTARGET and see no difficulties in obtaining the costs applicable to this study. Missing cost data will be estimated by using regression analysis similar to the technique used by Reed and Schulman but using local cost data rather than DRG to perform the regression. The total cost per patient will be translated into a single currency (CAD or USD) by using PPP (power purchasing parity) ratios.

5.0 Health care utilization

Resources consumed in the occlusion of the LAA will be recorded. This will consist of the method of the occlusion and quantity of the occlusion device used. Oral anti-coagulant use will also be recorded. All resources consumed in the treatment of strokes or systemic arterial embolism during this study will be included in the analysis. Other health care resources unrelated to the study i.e. cancer, orthopedic surgery, etc. would be ignored unless a significant difference between randomization groups is detected. Resources utilization is divided into 5 categories: oral anti-coagulants, stroke, systemic arterial embolism, and occlusion devices. Each category (average cost per patient) will be individually analyzed and a total average cost per patient will be provided.

5.1 Oral anti-coagulants

All oral anti-coagulants consumed by patient out of hospital will be recorded and a unit cost will be attributed. We will use the generic cost when available; otherwise the brand name cost will be used.

6.0 Analysis plan

Unit costs will be applied to patient-level utilization data to arrive at a cost per patient, and the average cost within each treatment group will be calculated. Since the cost data will not be normally distributed, a bootstrap analysis will be used to calculate standard errors and 95% confidence intervals for the difference in average costs. The bias corrected and accelerated (BCa) method will be used to obtain confidence intervals for average costs. With patient-level data for both clinical outcomes and costs available, a stochastic cost-effectiveness analysis will be performed. Comparisons between the two groups will be conducted using t-tests based on these estimates of standard error. We expect a very complete data set with the LAAOS III study. Nevertheless, some data could be missing. Patients with missing data will not be excluded from the analysis. Missing data will be replaced by the mean cost for the missing item (mean imputation).

7.0 Economic team

Dr. Andre Lamy, health economic scientist will be the project leader. He will be assisted by a health economic analyst (Wesley Tong). Statisticians from PHRI will also assist in the analysis. These statisticians have also helped in the economic analysis of HOPE, CURE, ONTARGET, ACTIVE-A, TIMACS, CORONARY and ORIGIN.

8.0 References

[i] Van de Werf F, Topol E, Lee KL, et al. Variations in patient management and outcomes for acute myocardial infarction in the United States and other countries: Results from the Gusto trial. Journal of the American Medical Association, 1995;273:1586-1591.
[ii] Lamy A, Yusuf S, Pogue J, Gafni A; Heart Outcomes Prevention Evaluation Investigators. Cost implications of the use of ramipril in high-risk patients based on the Heart Outcomes Prevention Evaluation (HOPE) study. Circulation. 2003 Feb 25;107(7):960-5.
[iii] Lamy A, Jonsson B, Weintraub WS, Zhao F, Chrolavicius S, Bakhai A, Culler S, Gafni A, Lindgren P, Mahoney E, Yusuf S; The CURE Economic Group (on behalf of the CURE Investigators). The cost-effectiveness of the use of clopidogrel in acute coronary syndromes in five countries based upon the CURE study. Eur J Cardiovasc Prev Rehabil. 2004 Dec;11(6):460-5.
[iv] Mahoney EM, Mehta S, Yuan Y, Jackson J, Chen R, Gabriel S, Lamy A, Culler S, Caro J, Yusuf S, Weintraub WS; CURE Study Investigators. Long-term cost-effectiveness of early and sustained clopidogrel therapy for up to 1 year in patients undergoing percutaneous coronary intervention after presenting with acute coronary syndromes without ST-segment elevation. Am Heart J. 2006; 151:219-227.
[v] Kolm P, Yong Y, Veledar E, Mehta SR, O'Brien JA, Weinstraub WS. Cost-effectiveness of clopidogrel in acute coronary syndromes in Canada: A long-term analysis based on the CURE trial. Can J Cardiol. 2007; 13:1037-1042.
[vi] Weintraub WS, Mahoney EM, Lamy A, Culler S, Yuan Y, Caro J, Gabriel S, Yusuf S; CURE Study Investigators. Long-term cost-effectiveness of clopidogrel given for up to one year in patients with acute coronary syndromes without ST-segment elevation. J Am Coll Cardiol. 2005; 45:838-845.
[vii] Lamy A, Tong W, Gao P, Chrolavicius S, Gafni A, Yusuf S, Connolly SJ. The Cost of Clopidogrel Use in Atrial Fibrillation in the ACTIVE-A Trial. Can J Cardiol. 2011; In Press. Epub ahead of print.
[viii] Reed SD, Anstrom KJ, Bakhai A, Briggs AH, Califf RM, Cohen DJ, Drummond MF, Glick HA, Gnanasakthy A, Hlatky MA, O'Brien BJ, Torti FM Jr, Tsiatis AA, Willan AR, Mark DB, Schulman KA. Conducting economic evaluations alongside multinational clinical trials: toward a research consensus. Am Heart J. 2005 Mar;149(3):434-43.
[ix] Lamy A, Wang X, Gao P, Tong W, Gafni A, Dans A, Avezum A, Ferreira R, Young J, Yusuf S, Teo K; ONTARGET Investigators. The cost implications of the use of telmisartan or ramipril in patients at high risk for vascular events: the ONTARGET study. J Med Econ. 2011; 14: 792-7.
[x] Lamy A, Yusuf S, Pogue J, Gafni A. Cost implications of the use of Ramipril in high-risk patients based on the Heart Outcomes Prevention Evaluation (HOPE Study) Circulation 2003 ;107 (7) .960-965
[xi] André Lamy, X. Wang, R. Kent, K, Smith, A. Gafni. Economic Evaluation of the MEDENOX trial: A Canadian Perspective. Canadian Respiratory Journal, Vol. 9(3), 169-77, May/June 2002
[xii] Andre Lamy, Susan Chrolavicius, Amiram Gafni, Salim Yusuf, Feng Zhao, Bengt Jonsson, Peter Lindgren, Ameet Bakhai, Marcus Flather, Keith Fox, Steve Culler, Elizabeth Mahoney, William Weintraub. The Cost-Effectiveness of the Use of

Clopidogrel in Acute Coronary Syndromes based upon the CURE Study. American Heart Association Meeting November 2002, Chicago Ill.
[xiii] Smith KM, Lamy A, Arthur HM, Gafni A, Kent R. Outcomes and costs of coronary artery bypass grafting: comparison between octogenarians and septuagenarians at a tertiary care centre. CMAJ 2001;165:759-764.
[xiv] Ontario Case Cost Project. Ontario Case Cost Project (OCCP): Ontario Guide to Case Costing, Version 1.1, Ottawa, Ontario Case Cost Project; 1995.

Description and Rationale of Protocol Modifications

Most Recently Authorized Study Protocol: Version 1.0, April 24, 2012

Amended Study Protocol for this Submission: Version 2.0, June 14, 2012

Additions are in bold text. Deletions are in red text.

**1.**	
**Topic**	Sample Size (Sections 3.2, 6.1, 6.4, 6.5)
**Modification**	The sample size has been increased to 4,700 patients from 3,500 patients.
**Rationale**	The sample size was increased as it was felt that a smaller effect size (25%), if present, would still impact practice by both the investigators and funding agencies.

Original

3.2 Expected number of subjects

The total sample size for the study is 1,750 patients per group, for a total of 3,500 patients. Patients will be recruited from approximately 60 centres worldwide.

6.1 Analysis population

All randomized … At least 1,750 patients will be equally allocated to the LAA occlusion arm or no LAA occlusion arm.

6.4 Data Safety Monitoring Board (DSMB)

The independent Data Safety Monitoring Board (DSMB) will …. Two formal interim analyses will be undertaken when 50% (140 events) and 75% (210 events) of the expected events have occurred…

6.5 Sample size calculation

This study will enroll 3,500 patients with an average follow-up of 4 years which will allow us to detect a 30% relative risk reduction (RRR) in the primary outcome with an expected control event rate of 2.5% per year. This trial would have 83% power, accounting for a 2%/year loss of patients due to competing death…

Table 3: Sample Size for Primary Outcome

Reduction in Hazard Ratio	Power		
	80%	85%	90%
30%	3170	3630	4240
35%	2260	2580	3020

…… This study is powered to have **83**% power to detect a **30**% relative risk reduction. A **30**% treatment effect …… For the purpose of this trial, we plan to have sufficient statistical power to detect a reduction of **30**%.

Revision

3.2 Expected number of subjects

The total sample size for the study is **2,350** patients per group, for a total of **4,700** patients. Patients will be recruited from approximately 60 centres worldwide

6.1 Analysis population

All randomized … At least **2,350** patients will be equally allocated to the LAA occlusion arm

or no LAA occlusion arm.

6.4 Data Safety Monitoring Board (DSMB)

The independent Data Safety Monitoring Board (DSMB) will .... Two formal interim analyses will be undertaken when 50% (**188** events) and 75% (**282** events) of the expected events have occurred...

6.5 Sample size calculation

This study will enroll **4,700** patients with an average follow-up of 4 years which will allow us to detect a **25**% relative risk reduction (RRR) in the primary outcome with an expected control event rate of 2.5% per year. This trial would have **80**% power, accounting for a 2%/year loss of patients due to competing death...

Table 3: Sample Size for Primary Outcome

Reduction in Hazard Ratio	Power		
	80%	85%	90%
**25%**	**4700**	**5380**	**6300**
30%	3170	3630	4240
35%	2260	2580	3020

...... This study is powered to have **80**% power to detect a **25**% relative risk reduction. A **25**% treatment effect ...... For the purpose of this trial, we plan to have sufficient statistical power to detect a reduction of **25**%.

**2.**	
**Topic**	Questionnaire Verifying Stroke Free Status (QVSFS) (Sections 5.2.2 and 5.2.5)
**Modification**	We are now requiring the QVSFS to be completed at the 6-month follow-up visit and at all other visits thereafter (instead of just at the yearly visits).
**Rationale**	We added the QVSFS to the intermittent visits (i.e. the half year visits) to ensure that any new strokes are not missed.

Original

5.2.2 Visit schedule

Table 2: Schedule of visits.

	In-hospital Phase			Follow-up Phase			
	Baseline (Pre-op)	Cardiac Surgery	Hospital Discharge (Post-op)	30-Day Clinic Visit	6-Month, 1.5, 2.5, 3.5-Year Telephone Visits[1]	1, 2, 3, 4, 5-Year Clinic Visits[1]	Final Clinic Follow-up Visit
QVSFS[3]						✓	✓

[1] As required until common end date
[2] Pre-operative rhythm can be within 30 days prior to surgery (results from most recent ECG should be used)
[3] Questionnaire Verifying Stroke Free Status

5.2.5 Follow-up Visits

Follow-up will occur .... adjudication of events.

The validated Questionnaire for Verifying Stroke Free Status (QVSFS) will be administered at the yearly visits until the Final Follow-up Visit.

Revision

5.2.3 Visit schedule

Table 2: Schedule of visits.

	In-hospital Phase			Follow-up Phase			
	Baseline (Pre-op)	Cardiac Surgery	Hospital Discharge (Post-op)	30-Day Clinic Visit	6-Month, 1.5, 2.5, 3.5-Year Telephone Visits[1]	1, 2, 3, 4, 5-Year Clinic Visits[1]	Final Clinic Follow-up Visit
QVSFS[3]					✓	✓	✓

[1] As required until common end date
[2] Pre-operative rhythm can be within 30 days prior to surgery (results from most recent ECG should be used)
[3] Questionnaire Verifying Stroke Free Status

5.2.6 Follow-up Visits

Follow-up will occur …. adjudication of events.

The validated Questionnaire for Verifying Stroke Free Status (QVSFS) will be administered at the **6-month follow-up and at all visits thereafter** until the Final Follow-up Visit.

Description and Rationale of Protocol Modifications

Most Recently Authorized Study Protocol: Version 2.0, June 14, 2012

Amended Study Protocol for this Submission: Version 3.0, January 18, 2013

Additions are in bold text. Deletions are in red text.

**1.**	
**Topic**	Inclusion Criteria
**Modification**	Added the inclusion criterion of CHA_2DS_2-VASc ≥ 2.
**Rationale**	Patients having cardiac surgery are an intrinsically high risk group. However, as per suggestions from CIHR, we have tightened the inclusion criteria by adding the requirement of a minimum CHA_2DS_2-VASc score of at least 2 to ensure patients in the trial have some risk of stroke. Patients will need to have a minimum of two known risk factors for stroke in order to be eligible.
**Original**	**4.1 Inclusion Criteria**<br><br>1) Age ≥ 18 years of age<br>2) Undergoing a clinically indicated cardiac surgical procedure with the use of cardiopulmonary bypass<br>3) A documented history of atrial fibrillation or atrial flutter<br>4) Written informed consent
**Revision**	**4.1 Inclusion Criteria**<br><br>1) Age ≥ 18 years of age<br>2) Undergoing a clinically indicated cardiac surgical procedure with the use of cardiopulmonary bypass<br>3) A documented history of atrial fibrillation or atrial flutter<br>**4) CHA_2DS_2-VASc score ≥ 2**<br>5) Written informed consent

**2.**	
**Topic**	Exclusion Criteria
**Modification**	Added the exclusion criterion of mechanical valve implantation.
**Rationale**	Mechanical heart valve patients are at elevated risk of the primary outcome, and a dominant portion of this risk will not be affected by the intervention. The steering committee felt that this group should not be included as they would dilute the effect size.
**Original**	**4.2 Exclusion Criteria**<br>1) Patients undergoing any of the following procedures:<br>a. Off-pump cardiac surgery<br>b. Heart transplant<br>c. Complex congenital heart surgery<br>d. Sole indication for surgery is ventricular assist device insertion<br>e. Previous cardiac surgery (re-operation)<br>2) Patients who have had a previous placement of a percutaneous LAA closure device
**Revision**	**4.2 Exclusion Criteria**<br>1) Patients undergoing any of the following procedures:<br>a. Off-pump cardiac surgery<br>b. Heart transplant<br>c. Complex congenital heart surgery<br>d. Sole indication for surgery is ventricular assist device insertion<br>e. Previous cardiac surgery (re-operation)<br>**f. Mechanical valve implantation**<br>2) Patients who have had a previous placement of a percutaneous LAA closure device

**3.**	
**Topic**	Study Intervention
**Modification**	Removed closure from within the left atrium as an allowable LAA occlusion technique.
**Rationale**	This modification was made in order to have a more standardized approach to the intervention.
**Original**	**5.1 <u>Intervention</u>**<br>The intervention under investigation is surgical occlusion of the left atrial appendage which is compared to no left atrial appendage occlusion. The trial will permit the following techniques of LAA occlusion: 1) amputation of the LAA and closure; 2) stapler closure of the LAA; and 3) closure of the LAA from within the right atrium. The preferred technique is amputation and closure as demonstrated by the video found at http://www.youtube.com/watch?v=aPoeoAhIjGw.
**Revision**	**5.1 <u>Intervention</u>**<br>The intervention under investigation is surgical occlusion of the left atrial appendage which is compared to no left atrial appendage occlusion. The trial will permit the following techniques of LAA occlusion: 1) amputation of the LAA and closure **(cut and sew)**; **or** 2) stapler closure of the LAA. The preferred technique is amputation and closure as demonstrated by the video found at http://www.youtube.com/watch?v=aPoeoAhIjGw.

**4.**	
**Topic**	Definitions of Study Outcomes
**Modification**	Modified the definition of major bleed.
**Rationale**	Standard definitions for major bleeding on OAC do not apply at the time of cardiac surgery, as highlighted by guideline suggestions for major bleeding definitions. We have therefore adapted the BARC definition of major bleeding for perioperative bleeding and the ISTH definition for non-perioperative.
**Original**	**5.6.3 Definitions of Study Outcomes**<br><br>***Major bleed***<br>Defined as per ISTH: 1) Fatal bleeding, and/or 2) Symptomatic bleeding in a critical area or organ, such as intracranial, intraspinal, intraocular, retroperitoneal, intra-articular or pericardial, or intramuscular with compartment syndrome, and/or 3) Bleeding causing a fall in hemoglobin level of 2.0 g/dL or more, or leading to transfusion of two or more units of whole blood or red cells.
**Revision**	**5.6.3 Definitions of Study Outcomes**<br><br>***Major bleed***<br>**Major bleeding within the first 48 hours after surgery is defined as per BARC Type 4: 1) Perioperative intracranial bleeding within 48 hours; and/or 2) Reoperation after closure of sternotomy for the purpose of controlling bleeding; and/or 3) Transfusion of ≥ 5 units whole blood or packed red blood cells within a 48 hour period (note: cell saver products are not counted); and/or 4) Chest tube output ≥ 2L within a 24 hour period.**<br><br>**Major bleeding after 48 hours after surgery** is defined as per **modified** ISTH: 1) Fatal bleeding, and/or 2) Symptomatic bleeding in a critical area or organ, such as intracranial, intraspinal, intraocular, retroperitoneal, intra-articular or pericardial, or intramuscular with compartment syndrome, and/or 3) Bleeding causing a fall in hemoglobin level of **3.0** g/dL* or more, or leading to transfusion of two or more units of whole blood or red cells.<br>***corrected for transfusion (1 unit PRBC or 1 unit whole blood = 1 g/dL hemoglobin)**

Description and Rationale of Protocol Modifications

Most Recently Authorized Study Protocol: Version 3.0, January 18, 2013

Amended Study Protocol for this Submission: Version 4.0, November 18, 2013

Protocol Amendment Approval Signature

Dr. Richard Whitlock<br>Co-Principal Investigator<br>Population Health Research Institute	Name	2013-11-19<br>Date (yyyy-mm-dd)

Additions are in bold text. Deletions are in red text.

1.	
**Topic**	Study Personnel Contact Information
**Modification**	Updated Project Manager to Shirley Pettit and updated Research Coordinator to Kate Brady.
**Rationale**	Changes made to reflect updated study personnel for the trial.

Original

Project Manager	Name:	Ms. Susan Chrolavicius
	Address:	Population Health Research Institute<br>237 Barton Street East, C1-240<br>Hamilton, ON L8L 2X2
	Tel:	905-527-4322 x 40365
	Fax:	905-297-3779
	Email:	Susan.Chrolavicius@phri.ca

Research Coordinator	Name:	Ms. Jessica Vincent
	Address:	Population Health Research Institute<br>237 Barton Street East, C1-239<br>Hamilton, ON L8L 2X2
	Tel:	905-527-4322 x 40635
	Fax:	905-297-3779
	Email:	Jessica.Vincent@phri.ca

Revision

Project Manager	Name:	Ms. **Shirley Pettit**
	Address:	Population Health Research Institute<br>237 Barton Street East, C1-**235**<br>Hamilton, ON L8L 2X2
	Tel:	905-527-4322 x **40526**
	Fax:	905-297-3779
	Email:	**Shirley.Pettit@phri.ca**

Research Coordinator	Name:	Ms. **Kate Brady**
	Address:	Population Health Research Institute<br>237 Barton Street East, C1-**242**<br>Hamilton, ON L8L 2X2
	Tel:	905-527-4322 x **41198**
	Fax:	905-297-3779
	Email:	**Kate.Brady@phri.ca**

2.	
**Topic**	Study Intervention (Sections 5.1 and 5.2.4)
**Modification**	Added atrial appendage closure devices as an allowable LAA occlusion technique.
**Rationale**	Addition of approved technique to reflect regulatory approval for atrial appendage closure devices.
**Original**	5.1 Intervention<br><br>Research personnel will provide... recorded on corresponding special CRFs.<br><br>The intervention under investigation is surgical occlusion of the left atrial appendage which is compared to no left atrial appendage occlusion. The trial will permit the following techniques of LAA occlusion: 1) amputation of the LAA and closure (cut and sew); or 2) stapler closure of the LAA. The preferred technique is amputation and closure as demonstrated by the video found at http://www.youtube.com/watch?v=aPoeoAhIjGw.<br><br>5.2.4 Cardiac Surgery to Hospital Discharge<br><br>Research personnel will provide... recorded on corresponding special CRFs.<br><br>The unblinded surgeon will be required to provide details regarding intervention compliance in a separate confidential database to maintain the study blind. Details of technique used (i.e. amputation and closure, or stapler device), and whether or not the occlusion was successful (as defined in Section 5.6.3) will be required for those patients who have undergone the LAA occlusion.
**Revision**	5.1 Intervention<br><br>Research personnel will provide... recorded on corresponding special CRFs.<br><br>The intervention under investigation is surgical occlusion of the left atrial appendage which is compared to no left atrial appendage occlusion. The trial will permit the following techniques of LAA occlusion: 1) amputation of the LAA and closure (cut and sew); 2) stapler closure of the LAA; **or 3) use of an atrial appendage closure device that is approved by the applicable governing regulatory authority.** The preferred technique is amputation and closure as demonstrated by the video found at http://www.youtube.com/watch?v=aPoeoAhIjGw.<br><br>5.2.4 Cardiac Surgery to Hospital Discharge<br><br>Research personnel will provide... recorded on corresponding special CRFs.<br><br>The unblinded surgeon will be required to provide details regarding intervention compliance in a separate confidential database to maintain the study blind. Details of technique used (i.e. amputation and closure, stapler device, **or atrial appendage closure device**), and whether or not the occlusion was successful (as defined in Section 5.6.3) will be required for those patients who have undergone the LAA occlusion.

3.	
**Topic**	Data Safety Monitoring Board (DSMB) (Section 6.4)
**Modification**	An error in the expected number of primary outcome events was corrected.
**Rationale**	An error was noted that the number of expected primary outcome events was not correctly updated during a previous protocol amendment when the sample size of the trial was increased.
**Original**	6.4 Data Safety Monitoring Board (DSMB)<br><br>The independent Data Safety Monitoring Board (DSMB) will ensure patient safety, receive and review interim analyses of efficacy data, provide feedback to the Steering Committee, and ensure the study follows the highest standards of ethics. Over the median follow-up of 4 years, we expect 280 primary outcome events. Two formal interim analyses will be undertaken when 50% (188 events) and 75% (282 events) of the expected events have occurred.
**Revision**	6.4 Data Safety Monitoring Board (DSMB)<br><br>The independent Data Safety Monitoring Board (DSMB) will ensure patient safety, receive and review interim analyses of efficacy data, provide feedback to the Steering Committee, and ensure the study follows the highest standards of ethics. Over the median follow-up of 4 years, we expect **385** primary outcome events. Two formal interim analyses will be undertaken when 50% (**193** events) and 75% (**289** events) of the expected events have occurred.

Description and Rationale of Protocol Modifications

Most Recently Authorized Study Protocol: Version 4.0, November 18, 2013

Amended Study Protocol for this Submission: Final Version 5.0, April 22, 2015

Protocol Amendment Approval Signature

Dr. Richard Whitlock<br>Co-Principal Investigator<br>Population Health Research Institute	Name	2015-04-22<br>Date (yyyy-mm-dd)

Additions are in bold text. Deletions are in red text.

**1.**	
**Topic**	Limitations of oral anticoagulation therapy (which LAA occlusion may mitigate) (Section 1.4)
**Modification**	An error of anticoagulant order and bleeding rate was corrected.
**Rationale**	Anticoagulants were listed in an incorrect order with respective bleeding rates.
**Original**	Increased bleeding, both major and minor is inherent in all antithrombotic therapy. For example, in the recent RE-LY Trial, the annual rates of major bleeding were 3.4%, 2.7% and 3.1% for dabigatran 110 mg BID, 150 mg BID and warfarin, respectively; and minor bleeding rates were 13%, 15% and 16% per year.
**Revision**	Increased bleeding, both major and minor is inherent in all antithrombotic therapy. For example, in the recent RE-LY Trial, the annual rates of major bleeding were **2.7%, 3.1% and 3.4%** for dabigatran 110 mg BID, 150 mg BID and warfarin, respectively; and minor bleeding rates were 13%, 15% and 16% per year.

**2.**	
**Topic**	Primary Objective (Section 2.1)
**Modification**	Adjustment of primary objective to include only ischemic stroke or transient ischemic attacks with positive neuroimaging or systemic arterial embolism.
**Rationale**	The study is blinded and advocates for stroke prevention antithrombotic therapy per guidelines. Left atrial appendage occlusion should not have any effect on hemorrhagic strokes.
**Original**	The primary objective is to examine the impact of LAA occlusion on the incidence of stroke or systemic arterial embolism in patients with atrial fibrillation undergoing cardiac surgery with the use of cardiopulmonary bypass over the duration of follow-up.
**Revision**	The primary objective is to examine the impact of LAA occlusion on the incidence of **ischemic** stroke*** or transient ischemic attack with positive neuroimaging** or systemic arterial embolism **over the duration of follow-up** in patients with atrial fibrillation undergoing cardiac surgery with the use of cardiopulmonary bypass.

**3.**	
**Topic**	Secondary Objectives (Section 2.2)
**Modification**	Separated secondary objectives and safety objectives. Added three additional secondary objectives. Changed order of objectives and modified wording for clarification.
**Rationale**	1) A composite objective examining all cause stroke or transient ischemic attack with positive neuroimaging or systemic arterial embolism will be included as the primary objective has been modified to exclude hemorrhagic strokes.<br>2) A composite objective examining ischemic stroke or transient ischemic attack with positive neuroimaging or systemic arterial embolism or death will be included to account for death as a major competing risk.<br>3) We will examine the impact of left atrial appendage occlusion on the incidence of non-perioperative (greater than 30 days from the date of surgery) ischemic stroke or transient ischemic attack with positive neuroimaging or systemic arterial embolism as cardiac surgery can result in perioperative strokes and LAA occlusion is not expected to modify the incidence of surgery-related strokes.<br><br>For clarity, the objectives have been re-ordered and the safety objectives have been separated.
**Original**	2.2 Secondary objectives<br><br>The secondary objectives are over duration of follow-up (unless otherwise specified) are:<br><br>1) To determine total mortality<br>2) To determine post-operative safety outcomes (chest tube output in the first 24 hours postsurgery, post-operative re-exploration for bleeding within 48 hours post-surgery, and 30-day mortality).<br>3) To determine the incidence of re-hospitalization for heart failure.<br>4) To determine the incidence of major bleeding.<br>5) To determine the incidence of myocardial infarction.
**Revision**	2.2 Secondary objectives<br><br>The secondary objectives are over duration of follow-up (unless otherwise specified) are:<br>**1) To determine the incidence of all cause stroke or transient ischemic attack with positive neuroimaging or systemic arterial embolism.**<br>**2) To determine the incidence of ischemic stroke* or transient ischemic attack with positive neuroimaging or systemic arterial embolism or death.**<br>**3) To determine the incidence of ischemic stroke* or transient ischemic attack with positive neuroimaging or systemic arterial embolism > 30 days after surgery.**<br>4) To determine **the incidence of all cause death.**<br><br>**2.3 Safety objectives**<br><br>1) To determine the incidence of hospitalization for heart failure.<br>2) To determine post-operative safety outcomes<br>a. Chest tube output in the first **post-operative** 24 hours<br>b. **Re-operation** for bleeding within 48 hours post-surgery<br>c. 30-day mortality<br>3) To determine the incidence of major bleeding.<br>4) To determine the incidence of myocardial infarction.<br><br>* Ischemic stroke is defined as any stroke that is not documented as primary hemorrhagic.

**4.**	
**Topic**	Type of Study (Section 3.2)
**Modification**	Update the number of expected sites participating.
**Rationale**	Increasing number of expected sites as projections indicate additional sites will be needed to accomplish recruitment within targeted timeframe of study.
**Original**	The total sample size for the study is 2,350 patients per group, for a total of 4,700 patients. Patients will be recruited from approximately 60 centres worldwide.
**Revision**	The total sample size for the study is 2,350 patients per group, for a total of 4,700 patients. Patients will be recruited from approximately **80-100** centres worldwide.

**5.**	
**Topic**	Exclusion Criteria (Section 4.2)
**Modification**	Added clarification to exclusion criterion 1.e)
**Rationale**	Clarification provided to previous cardiac surgery to differentiate between previous cardiac surgery and percutaneous procedures.
**Original**	1) Patients undergoing any of the following procedures:<br>a. Off-pump cardiac surgery<br>b. Heart transplant<br>c. Complex congenital heart surgery<br>d. Sole indication for surgery is ventricular assist device insertion<br>e. Previous cardiac surgery (re-operation)<br>f. Mechanical valve implantation
**Revision**	1) Patients undergoing any of the following procedures:<br>a. Off-pump cardiac surgery<br>b. Heart transplant<br>c. Complex congenital heart surgery<br>d. Sole indication for surgery is ventricular assist device insertion<br>e. Previous cardiac surgery **requiring opening of the pericardium**<br>f. Mechanical valve implantation

**6.**	
**Topic**	Intervention (Section 5.1)
**Modification**	Types of LAA occlusion techniques permitted were added.
**Rationale**	As mini thoracotomy procedures are becoming more frequent, an occlusion technique for these cases will be accepted to reduce barriers to patient recruitment and to expand the generalizability of the study results. The Operations Committee recognizes that other occlusion methods or variations in technique may be employed at various institutions and wants to allow for inclusion of those methods provided that there are corresponding efficacy data warranting such inclusion.
**Original**	5.1 Intervention<br><br>The intervention under investigation is surgical occlusion of the left atrial appendage which is compared to no left atrial appendage occlusion. The trial will permit the following techniques of LAA occlusion: 1) amputation of the LAA and closure (cut and sew); 2) stapler closure of the LAA; or 3) use of an atrial appendage closure device that is approved by the applicable governing regulatory authority. The preferred technique is amputation and closure as demonstrated by the video found at http://www.youtube.com/watch?v=aPoeoAhIjGw.
**Revision**	5.1 Intervention<br><br>The intervention under investigation is surgical occlusion of the left atrial appendage which is compared to no left atrial appendage occlusion. The trial will permit the following techniques of LAA occlusion: 1) amputation of the LAA and closure (cut and sew); 2) stapler closure of the LAA; 3) use of an atrial appendage closure device that is approved by the applicable governing regulatory authority**; or 4) double layer linear closure from within the atrium is acceptable for mini thoracotomy procedures if successful closure will be confirmed by TEE. Other LAA interventions proven to be efficacious may be proposed for use and approved by the Operations Committee on a case-by-case basis with such approval clearly documented. The use of simple purse string closure of the LAA is strictly prohibited.** The preferred technique is amputation and closure as demonstrated by the video found at http://www.youtube.com/watch?v=aPoeoAhIjGw.

**7.**	
**Topic**	Visit Schedule (Section 5.2.2)
**Modification**	Oversight of INR collection at 6-Month, 1.5, 2.5, 3.5, 4.5-Year Telephone Visits corrected.
**Rationale**	For patients taking a vitamin K antagonist, there was an oversight in the visit schedule to include collection of patient's most recent INR per standard of care at the 6-Month, 1.5-Year, 2.5-Year, 3.5-Year, 4.5-Year telephone call visits and this oversight is now corrected.
**Original**	(see table below)

	In-hospital Phase			Follow-up Phase			
	Baseline (Pre-op)	Cardiac Surgery	Hospital Discharge (Post-op)	30-Day Clinic Visit	6-Month, 1.5, 2.5, 3.5, 4.5-Year Telephone Visits[1]	1, 2, 3, 4, 5-Year Clinic Visits[1]	Final Clinic Follow-up Visit
INR (if applicable)	✓		✓	✓		✓	✓

<table>
<tr><td>Revision</td><td>
<table>
<tr><td></td><td colspan="3">In-hospital Phase</td><td colspan="4">Follow-up Phase</td></tr>
<tr><td></td><td>Baseline (Pre-op)</td><td>Cardiac Surgery</td><td>Hospital Discharge (Post-op)</td><td>30-Day Clinic Visit</td><td>6-Month, 1.5, 2.5, 3.5, 4.5-Year Telephone Visits[1]</td><td>1, 2, 3, 4, 5-Year Clinic Visits[1]</td><td>Final Clinic Follow-up Visit</td></tr>
<tr><td>INR (if applicable)</td><td>✓</td><td></td><td>✓</td><td>✓</td><td>✓</td><td>✓</td><td>✓</td></tr>
</table>
</td></tr>
</table>

**8.**	
**Topic**	Follow-up Visits (Section 5.2.5)
**Modification**	Clarification that all follow-up visits may occur by telephone.
**Rationale**	The follow-up visits do not have assessments that necessitate an in-person follow-up visit so clarification is made that these visits can also be conducted by telephone for practicality and to accommodate patients with geographic barriers. This is also footnoted in Table 2 Schedule of Visits.
**Original**	5.2.5 Follow-up Visits<br>Follow-up will occur at 30 days after randomization, at one year and annually thereafter until the final follow-up visit (the common study end date is expected to be determined at approximately 5 years after the first patient is randomized). Patients will be contacted at 6 month intervals by telephone to avoid patients lost to follow-up. Event CRFs should be completed as soon as the investigating site becomes aware of the event. Supporting documentation for each event is required and should be forwarded to the LAAOS III Project Office as soon as it is available to ensure timely adjudication of events.
**Revision**	5.2.5 Follow-up Visits<br>Follow-up will occur at 30 days after randomization, at one year and annually thereafter until the final follow-up visit (the common study end date is expected to be determined at approximately 5 years after the first patient is randomized). **Follow-up visits may occur as office visits or by telephone.** Patients will be contacted at 6 month intervals by telephone to avoid patients lost to follow-up. Event CRFs should be completed as soon as the investigating site becomes aware of the event. Supporting documentation for each event is required and should be forwarded to the LAAOS III Project Office as soon as it is available to ensure timely adjudication of events.

**9.**	
**Topic**	Encouraging compliance (Section 5.4)
**Modification**	Added the clarification that surgeon's occlusion method of choice should be approved.
**Rationale**	Various left atrial appendage techniques exist and other techniques may be developed over the course of the trial. It is important to highlight that a technique should be approved by the Operations Committee to ensure that the technique is associated with anatomical LAA occlusion and a low risk for late failure of LAA occlusion.
**Original**	5.4 Encouraging Compliance<br>Intervention non-compliance is not expected to be a major issue in this trial. The intervention…concise. Surgeons are permitted to use their occlusion method of choice for patients randomized to this arm and therefore compliance with the allocated intervention is expected to be high. However, we do foresee circumstances where the surgeon may elect not to occlude the appendage (e.g. unrecognized adhesions or other anatomical considerations). Pilot work suggests that the frequency of such an occurrence will be minimal (less than 1% of cases).
**Revision**	5.4 Encouraging Compliance

	Intervention non-compliance is not expected to be a major issue in this trial. The intervention…concise. Surgeons are permitted to use their **approved** occlusion method of choice for patients randomized to this arm and therefore compliance with the allocated intervention is expected to be high. However, we do foresee circumstances where the surgeon may elect not to occlude the appendage (e.g. unrecognized adhesions or other anatomical considerations). Pilot work suggests that the frequency of such an occurrence will be minimal (less than 1% of cases).

**10.**	
**Topic**	Primary Outcome(Section 5.6.1)
**Modification**	Adjustment of primary outcome to include only ischemic stroke or transient ischemic attacks with positive neuroimaging or systemic arterial embolism.
**Rationale**	The study is blinded and advocates for stroke prevention antithrombotic therapy per guidelines. Left atrial appendage occlusion should not have any effect on hemorrhagic strokes. Transient ischemic attacks with positive neuroimaging will be upgraded to strokes during blinded adjudication and included in primary outcome based on recommendations from the American Stroke Association and the American Heart Association.
**Original**	The primary outcome is the first occurrence of stroke or systemic arterial embolism over the duration of follow-up.
**Revision**	The primary outcome is the first occurrence of **ischemic*** stroke **or transient ischemic attack with positive neuroimaging** or systemic arterial embolism over the duration of follow-up.

**11.**	
**Topic**	Secondary Outcomes (Section 5.6.2)
**Modification**	Separated secondary outcomes and safety outcomes. Added three additional secondary outcomes. Changed order of outcomes and modified wording for clarification.
**Rationale**	1) A composite outcome examining all cause stroke or transient ischemic attack with positive neuroimaging or systemic arterial embolism will be included as the primary outcome has been modified to exclude hemorrhagic ischemic strokes.<br>2) A composite outcome examining ischemic stroke or transient ischemic attack with positive neuroimaging or systemic arterial embolism or death will be included to account for death as a major competing risk.<br>3) We will examine the impact of left atrial appendage occlusion on the incidence of non-perioperative (greater than 30 days from the date of surgery) ischemic stroke or transient ischemic attack with positive neuroimaging or systemic arterial embolism as cardiac surgery can result in perioperative strokes and LAA occlusion is not expected to modify the incidence of surgery-related strokes.<br><br>For clarity, the outcomes have been re-ordered and the safety outcomes have been separated.
**Original**	5.6.2 Secondary Outcomes<br>The secondary outcomes over the duration of follow-up (unless otherwise specified) are:<br>1) Total mortality<br>2) Operative safety outcomes (chest tube output in the first post-operative 24 hours, rate of post-operative re-exploration for bleeding in the first 48 hours post-surgery and 30-day mortality)<br>3) Re-hospitalization for heart failure<br>4) Major bleed<br>5) Myocardial infarction

**Revision**	5.6.2 Secondary Outcomes<br>The secondary outcomes over the duration of follow-up (unless otherwise specified) are:<br>**1) All cause stroke or transient ischemic attack with positive neuroimaging or systemic arterial embolism**<br>**2) Composite of ischemic stroke* or transient ischemic attack with positive neuroimaging or systemic arterial embolism or death**<br>**3) Ischemic stroke* or transient ischemic attack with positive neuroimaging or systemic arterial embolism occurring > 30 days after surgery**<br>**4) All cause death**<br><br>5.6.3 Safety Outcomes<br><br>1) Hospitalization for heart failure<br>2) Operative safety outcomes<br>a. Chest tube output in the first post-operative 24 hours<br>b. **Re-operation** for bleeding **within** the first 48 hours post-surgery<br>c. 30-day mortality<br>3) Major bleed<br>4) Myocardial infarction<br><br>*** Ischemic stroke is defined as any stroke that is not documented as primary hemorrhagic.**<br>**† All components of composite outcomes will also be reported individually.**

**12.**	
**Topic**	Definitions of Study Outcomes (Section 5.6.4)
**Modification**	Updated stroke definition to include transient ischemic attack (TIA) with positive neuroimaging.
**Rationale**	Based on recommendations from the American Stroke Association and the American Heart Association.
**Original**	Stroke<br>Diagnosis of stroke will require new focal neurological symptoms with rapid onset, lasting at least 24 hours. All strokes will be classified as definite ischemic, definite hemorrhagic or type uncertain.
**Revision**	Stroke<br>Diagnosis of stroke will require new focal neurological symptoms with rapid onset, lasting at least 24 hours. All strokes will be classified as definite ischemic, definite hemorrhagic or type uncertain. **Transient ischemic attacks (TIAs) with positive neuroimaging will be upgraded to stroke during blinded outcome adjudication.**

**13.**	
**Topic**	Definitions of Study Outcomes (Section 5.6.4)
**Modification**	Added definition for transient ischemic attack (TIA).
**Rationale**	Transient ischemic attacks are being collected in the trial as clinical outcomes and adjudicated to ensure that the event should not be upgraded to a stroke and so an event definition has been included.
**Original**	N/A

**Revision**	**Transient Ischemic Attack (TIA)**<br>**An episode of a new focal neurologic deficit with rapid onset with signs or symptoms lasting <24 hours. TIAs with positive neuroimaging should be classified as a stroke, regardless of duration of symptoms.**

**14.**	
**Topic**	Definitions of Study Outcomes (Section 5.6.4)
**Modification**	Removed adjudication information from sub-classification of death.
**Rationale**	Death will not be adjudicated, sub-classification of death as cardiovascular or non-cardiovascular will be as reported by site personnel.
**Original**	Sub-Classification of Death<br>All deaths will be classified as either cardiovascular or non-cardiovascular. Cardiovascular death is defined as any death with a cardiovascular cause and includes those deaths occurring within 30 days of a cardiovascular procedure (e.g. cardiac surgery, percutaneous transluminal coronary angioplasty), cardiac arrest, myocardial infarction, pulmonary embolus, stroke, hemorrhage, or deaths due to an unknown cause. Non-cardiovascular death is defined as deaths due to a clearly documented non-cardiovascular cause (e.g. trauma, infection, malignancy). The research personnel will forward the Event Adjudication Committee all relevant clinical notes, laboratory tests, diagnostic imaging reports, and autopsy information from any patient who dies.
**Revision**	Sub-Classification of Death<br>All deaths will be classified as either cardiovascular or non-cardiovascular. Cardiovascular death is defined as any death with a cardiovascular cause and includes those deaths occurring within 30 days of a cardiovascular procedure (e.g. cardiac surgery, percutaneous transluminal coronary angioplasty), cardiac arrest, myocardial infarction, pulmonary embolus, stroke, hemorrhage, or deaths due to an unknown cause. Non-cardiovascular death is defined as deaths due to a clearly documented non-cardiovascular cause (e.g. trauma, infection, malignancy).
