# Supplementary material for: Stroke Mechanism and Severity After Left Atrial Appendage Occlusion: Insights From the LAAOS III Randomized Clinical Trial
Source: JAMA Neurol. 2025 Nov 17;83(1):76–82. doi: 10.1001/jamaneurol.2025.4478 (PMC12624455; doi:10.1001/jamaneurol.2025.4478)
Supplement: Supplement 2. — Statistical Analysis Plan. [file jamaneurol-e254478-s002.pdf]

DocuSign Envelope ID: 58EBE868-868F-44D0-B13A-68108E4DA309

LAAOS III

Left Atrial Appendage Occlusion Study III

STATISTICAL ANALYSIS PLAN

FINAL VERSION 1.0

January 28, 2021

Statistical Analysis Plan v1.0 Approvals:

By signing the below, I designate my approval of the above-named version of the Statistical Analysis Plan.

Co- Principal Investigator: Dr. Richard Whitlock

Signature: DocuSigned by: Dr. Richard Whitlock 933778A89E0347E... Date: 2021-Jan-28 | 09:46 PST
yyyy-mm-dd

Co- Principal Investigator: Dr. Stuart Connolly

Signature: DocuSigned by: Stuart Connolly B45F34C28A8243C... Date: 2021-Jan-28 | 13:23 EST
yyyy-mm-dd

Statistician: Patrick Sheridan

Signature: DocuSigned by: Patrick Sheridan 91169F04CFCD477... Date: 2021-Jan-28 | 13:08 EST
yyyy-mm-dd

Version History:

Version	Date	Author	Summary of Changes
1.0	2021-01-28	Richard Whitlock	First finalized version.

DocuSign Envelope ID: 58EBE868-868F-44D0-B13A-68108E4DA309

Table of Contents

1 List of Abbreviations 4
2 Background and Study Design 4
3 Study Hypothesis 5
4 Study Objectives 5
4.1 Primary Objective 5
4.2 Secondary Objectives 5
4.2.1 Safety objectives 5
5 Population to be Analyzed 6
6 Baseline and Operative Characteristics 6
7 Treatment Compliance 6
8 Lost to Follow-up 6
9 Outcomes and Definitions 7
9.1 Primary Outcome 7
9.2 Secondary Outcomes 7
9.2.1 Efficacy Outcomes 7
9.2.2 Safety Outcomes 7
9.3 Definitions of Outcomes 8
10 Outcome Analyses 9
10.1 Analyses of Primary Outcomes 9
10.2 Analyses of Secondary Outcomes 9
10.2.1 Analysis of Efficacy Outcomes 9
10.2.2 Analyses of Safety Outcomes 10
11 Subgroup Analysis 10
12 Adherence to the Protocol 11
13 Sensitivity Analyses 11
13.1 .... Landmark Analysis (Landmark at 30 days) 11
13.2 .... Per Protocol Analysis 12
13.3 .... As Treated Analysis 12
14 APPENDIX A: Table Outlines 13

1 List of Abbreviations

Abbreviation	Definition
AF	Atrial fibrillation
$CHADS_2$ Score	Congestive heart failure (1 point), Hypertension (1 point), Age >75 (2 points), Diabetes Mellitus (1 point), Stroke or TIA (2 points)
CHA_2DS_2-VASc Score	Congestive heart failure/LV dysfunction (1 point), Hypertension (1 point), Age ≥ 75 (2 points), Diabetes Mellitus (1 point), Stroke/TIA/ thromboembolism (2 points), Vascular disease (prior MI, PAD or aortic plaque) (1 point), Age 65-74 (1 point), Sex (1 point for female)
CPB	Cardiopulmonary bypass
CRF	Case report form
DSMB	Data safety monitoring board
ECG	Electrocardiogram
ICU	Intensive care unit
INR	International normalized ratio
IRB	Institutional Review Board
LA	Left atrium
LAA	Left atrial appendage
LAAOS	Left atrial appendage occlusion study
LVEF	Left ventricular ejection fraction
MI	Myocardial infarction
OAC	Oral anticoagulant
PHRI	Population Health Research Institute
QVSFS	Questionnaire Verifying Stroke Free Status
RBC	Red blood cells
RCT	Randomized controlled trial
REB	Research Ethics Board
RR	Relative risk
TEE	Transesophogeal Echocardiogram
TIA	Transient ischemic attack
VKA	Vitamin K Antagonist

2 Background and Study Design

Atrial fibrillation (AF) is present in 10% of patients coming for cardiac surgery, resulting in an annual stroke risk of about 4.5%. Studies indicate that a large proportion of strokes in AF come from the left atrial appendage (LAA). Two moderately-sized trials using percutaneous LAA closure devices suggest that LAA occlusion may reduce stroke; however, these studies are underpowered, employ expensive technology, and exclude patients with valvular disease who represent over 50% of AF patients having cardiac surgery. Surgical LAA occlusion at the time of heart surgery is a safe, one-time, $10 intervention, which unlike oral anticoagulation is not susceptible to non-compliance and non-persistence. Each year in North America, there are over 500,000 open-heart surgeries performed. Techniques for complete surgical LAA occlusion are now well defined and well established. Thus, if LAAOS III demonstrates benefit, practice guidelines will change and LAA occlusion will rapidly become standard adjunctive therapy for AF patients undergoing cardiac surgery, preventing hundreds of strokes per year in Canada alone.

DocuSign Envelope ID: 58EBE868-868F-44D0-B13A-68108E4DA309

Study Design:
Patients are randomized to surgical LAA occlusion, or not, at the time of cardiac surgery. All patients continue to receive antithrombotic therapy according to international guidelines. Patients, research nurses, and primary care physicians are blinded to treatment arm. Patients ≥18 years undergoing a cardiac surgery with documented atrial fibrillation/flutter and a CHA2DS2-VASc score ≥ 2 are eligible.

3 Study Hypothesis
Our hypothesis is that LAA occlusion will reduce stroke and will benefit virtually all AF patients if completed at the time of routine cardiac surgery.

4 Study Objectives

4.1 Primary Objective
The primary objective is to examine the impact of LAA occlusion on the incidence of ischemic stroke* or transient ischemic attack with positive neuroimaging or systemic arterial embolism over the duration of follow-up in patients with atrial fibrillation undergoing cardiac surgery with the use of cardiopulmonary bypass.

4.2 Secondary Objectives
The secondary objectives over duration of follow-up (unless otherwise specified) are to determine the impact of LAA occlusion on:

1) all-cause stroke or transient ischemic attack with positive neuroimaging or systemic arterial embolism,

2) ischemic stroke* or transient ischemic attack with positive neuroimaging or systemic arterial embolism or death,

3) ischemic stroke* or transient ischemic attack with positive neuroimaging or systemic arterial embolism > 30 days after surgery,

4) all-cause death.

4.2.1 Safety objectives
The safety objectives over duration of follow-up (unless otherwise specified) are to determine the impact of LAA occlusion on:

1) readmission to hospital for heart failure (time to first event and total occurrences),

2) post-operative safety outcomes:
    a. Chest tube output in the first post-operative 24 hours,
    b. Re-operation for bleeding within 48 hours post-surgery,

c. 30-day mortality,

3) major bleeding,

4) myocardial infarction.

* Ischemic stroke is defined as any stroke that is not documented as primary hemorrhagic. All components of the composite will be reported individually.

5 Population to be Analyzed

The intention to treat principle (ITT), in which all participants who underwent cardiac surgery will be included in their assigned treatment groups regardless of adherence, will guide all analyses.

A secondary analysis of the primary, secondary, and safety outcomes will be performed on 1) ITT on all participants irrespective of whether they underwent cardiac surgery, 2) per protocol (including only the patients who were treated as allocated, therefore excluding treatment arm crossovers) , and 3) as treated (including all patients based on whether they had their LAA occluded or not, therefore excluding any patients that did not have cardiac surgery due to cancellation or death prior to surgery) bases.

6 Baseline and Operative Characteristics

Baseline and operative characteristics by group will be reported in tabular format by treatment (see Appendix A).

7 Treatment Compliance

In patients who underwent surgery, we will assess treatment compliance by calculating the proportion of patients that received the treatment as per allocation in each group. We will report the proportion of patients randomized but who never underwent surgery. The average time from randomization to surgery will be reported by group. The use of oral anticoagulants in follow-up will reported by group. For patients on VKA, the time in therapeautic range and the proportion of patients on OAC at each follow-up.

8 Lost to Follow-up

Every attempt will be made to minimize the percent of individuals who are lost to follow-up. When a patient does not attend a visit, at least 3 further attempts to ascertain vital status and /or stroke status will be made through contacting relatives, friends, neighbours and examining physician and hospital charts.

Individuals who are still lost to follow-up will have their follow-up censored at their last date of contact. As a sensitivity analysis under the assumption of missing at random, a

DocuSign Envelope ID: 58EBE868-868F-44D0-B13A-68108E4DA309

propensity score for the probability of having a primary outcome event will be built using baseline factors in the study population. The predicted score will then be calculated for each lost to follow-up patient and those lost to follow-up patients whose predicted probability of an event is greater than the observed probability for the study population as a whole will be assumed to have had an event at the next day of the last follow-up. For those lost to follow-up patients not assumed to have had an event, their follow-up time will be the median length of follow-up of the trial.

9 Outcomes and Definitions

9.1 Primary Outcome

The primary outcome is the first occurrence of ischemic stroke or type uncertain stroke or transient ischemic attack with positive neuroimaging or systemic arterial embolism over the duration of follow-up.
* Ischemic stroke is defined as any stroke that is not documented as primary hemorrhagic.

9.2 Secondary Outcomes

Secondary outcomes include:

9.2.1 Efficacy Outcomes

1) all-cause stroke or transient ischemic attack with positive neuroimaging or systemic arterial embolism

2) composite of ischemic stroke or transient ischemic attack with positive neuroimaging or systemic arterial embolism or death

3) ischemic stroke or transient ischemic attack with positive neuroimaging or systemic arterial embolism occurring > 30 days after surgery

4) all-cause death.

   * Ischemic stroke is defined as any stroke that is not documented as primary hemorrhagic.

9.2.2 Safety Outcomes

1) Hospitalization for heart failure
2) Operative safety outcomes
   a) Chest tube output in the first post-operative 24 hours
   b) Re-operation for bleeding within the first 48 hours post-surgery
   c) 30-day mortality
3) Major bleed
4) Myocardial infarction

DocuSign Envelope ID: 58EBE868-868F-44D0-B13A-68108E4DA309

9.3 Definitions of Outcomes

Stroke
Diagnosis of stroke will require new focal neurological symptoms with rapid onset, lasting at least 24 hours. All strokes will be classified as definite ischemic, definite hemorrhagic or type uncertain. Transient ischemic attacks (TIAs) with positive neuroimaging will treated as an ischemic stroke.

Transient ischemic attack (TIA)
An episode of a new focal neurologic deficit with rapid onset with signs or symptoms lasting <24 hours. TIAs with positive neuroimaging should be classified as a stroke, regardless of duration of symptoms.

Systemic arterial embolism
Systemic arterial embolism will be judged to occur where there is a clinical history consistent with an acute loss of blood flow to a peripheral artery (or arteries), which is supported by objective evidence of embolism.

Major bleeding
Major bleeding **within the first 48 hours after surgery** is defined as per BARC Type 4: 1) Perioperative intracranial bleeding within 48 hours; and/or 2) Reoperation after closure of sternotomy for the purpose of controlling bleeding; and/or 3) Transfusion of ≥ 5 units whole blood or packed red blood cells within a 48 hour period (note: cell saver products are not counted); and/or 4) Chest tube output ≥ 2L within a 24 hour period.

Major bleeding **after 48 hours after surgery** is defined as per modified ISTH: 1) Fatal bleeding, and/or 2) Symptomatic bleeding in a critical area or organ, such as intracranial, intraspinal, intraocular, retroperitoneal, intra-articular or pericardial, or intramuscular with compartment syndrome, and/or 3) Bleeding causing a fall in hemoglobin level of 3.0 g/dL* or more, or leading to transfusion of two or more units of whole blood or red cells.
* corrected for transfusion (1 unit PRBC or 1 unit whole blood = 1 g/dL hemoglobin)

Hospitalization with heart failure
Re-hospitalization with an overnight stay or prolongation of an existing hospitalization due to heart failure which requires both clinical (i.e. any of the following signs: elevated jugular venous pressure, respiratory râles, crepitations, or presence of S3) and radiographic evidence (e.g. vascular redistribution, interstitial pulmonary edema, or frank alveolar pulmonary edema).

Efficacy of Occlusion Technique
Successful occlusion is defined as TEE Doppler assessment demonstrating an absence of flow across the suture line and a stump of <1 cm.

Myocardial infarction
Perioperative MI (≤48 hours post-operatively) is defined as the presence of new Q-waves or a new left bundle branch block on electrocardiogram, combined with a biomarker (CK-

MB or troponin) elevation of at least 5 times the upper reference limit. **Late MI** (>48 hours) is defined as ischemic symptoms, ECG changes consistent with myocardial infarction (new significant Q waves in two contiguous leads) or evolving ST-segment or T-wave changes in two contiguous leads signifying ischemia or new left bundle branch block (LBBB) or ST segment elevation and elevated cardiac markers (troponins or CK-MB) in the necrosis range. Myocardial injury occurring after a percutaneous coronary intervention (PCI) are included in the late perioperative MI group but are defined as elevation of cardiac markers at least 3 times upper limit of normal (ULN) within 24 hours of percutaneous coronary intervention (PCI) or characteristic evolution of new ECG changes.

24-Hour Chest Tube Output
Total chest tube output in the first 24 hours or until the tubes are removed, whichever comes earlier.

10 Outcome Analyses

For all outcome analyses, time 0 is defined as the start time of index surgery unless otherwise specified.

10.1 Analyses of Primary Outcomes

The ITT principle will guide the analyses unless otherwise specified. A time-to-event analysis will be used to test the primary outcome variable. The primary outcome will be presented using Kaplan-Meier survival curves and be compared between groups using a log rank test. The treatment effect as measured by the hazard ratio and 95% confidence interval will be derived by the Cox proportional hazards model. A p-value of <0.05 for the proportional hazards model will be considered as significant. The proportional hazards assumption will also be tested by graphical means.

10.2 Analyses of Secondary Outcomes

10.2.1 Analysis of Efficacy Outcomes

1) **All cause stroke or transient ischemic attack with positive neuroimaging or systemic arterial embolism:** Time-to-event analysis as per the primary outcome.

2) **Ischemic stroke* or transient ischemic attack with positive neuroimaging or systemic arterial embolism or death:** Time-to-event analysis as per the primary outcome.

3) **Ischemic stroke* or transient ischemic attack with positive neuroimaging or systemic arterial embolism occurring > 30 days after surgery.** We will only analyze the patients who have survived the first 30 days after surgery, irrespective of whether experienced a primary outcome event in the first 30 days. The primary outcome will be presented using Kaplan-Meier survival curves and compared between groups using a

DocuSign Envelope ID: 58EBE868-868F-44D0-B13A-68108E4DA309

log rank test. The treatment effect as measured by the hazard ratio and 95% confidence interval will be derived by the Cox proportional hazards model. A p-value of <0.05 for the proportional hazards model will be considered as significant. The proportional hazards assumption will also be tested by graphical means.

4) **All cause death:** Time-to-event analysis as per the primary outcome.

* Ischemic stroke is defined as any stroke that is not documented as primary hemorrhagic.

10.2.2 Analyses of Safety Outcomes

1) **Hospitalization for heart failure:** Time-to-event analysis as per the primary outcome.

2) **Operative safety outcomes**

   a) **Chest tube output in the first post-operative 24 hours:** Mean chest tube output within the first 24 hours post-operatively will be calculated in milliliters for each group (with associated standard deviation) and compared via a student t-test, or Mann-Whitney U test if nonparametric.

   b) **Major bleeding within 48 hrs of operation:** We will calculate the frequency of re-operation for bleeding within the first 48 hours post-operatively for each group and compare with a Pearson chi-square test. We will calculate the relative risk and corresponding 95% confidence intervals for this event.

   c) **Re-operation for bleeding within the first 48 hours post-surgery:** We will calculate the frequency of re-operation for bleeding within the first 48 hours post-operatively for each group and compare with a Pearson chi-square test. We will calculate the relative risk and corresponding 95% confidence intervals for this event.

   d) **30-day mortality:** We will calculate the frequency of death within the first 30 days post-operatively for each group and compare via a Pearson chi-square test. We will calculate the relative risk and corresponding 95% confidence intervals for this event.

3) **Major bleed after 48 hrs after surgery:** Time-to-event analysis.

4) **Myocardial infarction:** Time-to-event analysis.

11 Subgroup Analysis

Additional Cox models will be used to evaluate interactions between treatment and subgroups of interest: sex (male vs. female), rheumatic heart disease (yes vs. no), OAC used at baseline (DOAC vs. VKA vs. none), successful occlusion by trial definition (yes vs. no),

CHA_2DS_2-VASc score (<=4vs. >4), surgery type (Any valve vs. all others), and concomitant atrial ablation procedure (yes vs. no).

The test of interaction between each subgroup factor and the treatment group will be done by including a product term in the model already containing treatment and the subgroup factor. Significant interactions in the analysis of the co-primary outcomes will be interpreted as "flags" to prompt further investigation. Following the test of interaction, the treatment effect will be estimated separately within each level of a subgroup variable using the logistic regression model that was used in the main analysis for the outcome of interest. Table 1 summarizes the subgroups and the a priori hypothesis for each.

Table 1. Variables for Subgroup Analysis

Variable	Subgroups	A Priori Hypothesis
Sex	Male vs. female	LAA occlusion may be more protective in women than men
Rheumatic heart disease	Yes vs. no	LAA occlusion is less protective in patients with rheumatic heart disease
OAC at baseline	DOAC vs. VKA vs. neither	Patients with no OAC derive greater benefit
CHA_2DS_2-VASc score		Patients with higher score derive greater benefit
Surgery type	(Any valve vs. all others)	Patients having valve surgery derive less benefit
Atrial ablation procedure	Yes vs. no	Patients having atrial ablation derive less benefit
Successful occlusion	Yes vs. no	Patients with successful occlusion derive greater benefit

12 Adherence to the Protocol

Summary statistics (counts and percentage of patients) for reported protocol deviations will be presented as the total number of deviations and deviations within each category (e.g. inclusion/exclusion criteria, randomization, wrong treatment received).

13 Sensitivity Analyses

13.1 Landmark Analysis (Landmark at 30 days)

1) **Hospitalization for heart failure:** We will only analyze the patients who have survived without experiencing a hospitalization for heart failure in the first 30 days after surgery. Using landmark analysis at 30 days, as per the primary outcome.

DocuSign Envelope ID: 58EBE868-868F-44D0-B13A-68108E4DA309

13.2 Per Protocol Analysis

1) Including only the patients who were treated as allocated, a time-to-event analysis will be used to test the primary outcome variable. The primary outcome will be presented using Kaplan-Meier survival curves and be compared between groups using a log rank test. The treatment effect as measured by the hazard ratio and 95% confidence interval will be derived by the Cox proportional hazards model. A p-value of <0.05 for the proportional hazards model will be considered as significant. The proportional hazards assumption will also be tested by graphical means.

2) **Hospitalization for heart failure:** Per protocol time-to-event analysis as per the primary outcome.

13.3 As Treated Analysis

1) Including all patients based on whether they had their LAA occluded or not, a time-to-event analysis will be used to test the primary outcome variable. The primary outcome will be presented using Kaplan-Meier survival curves and be compared between groups using a log rank test. The treatment effect as measured by the hazard ratio and 95% confidence interval will be derived by the Cox proportional hazards model. A p-value of <0.05 for the proportional hazards model will be considered as significant. The proportional hazards assumption will also be tested by graphical means.

2) **Hospitalization for heart failure:** As treated time-to-event analysis as per the primary outcome.

DocuSign Envelope ID: 58EBE868-868F-44D0-B13A-68108E4DA309

14 APPENDIX A: Table Outlines

Table 2. Baseline Characteristics

Characteristic	Occlusion	No Occlusion
**Demographics**		
Age (SD)		
Type of AF<br>paroxysmal<br>persistent<br>permanent		
First AF documentation<br><6 months<br>6 months – 2 years<br>>2 years		
Male (%)		
Height (cm)		
Weight (kg)		
Preop creatinine (mmol/L)		
Preop hemoglobin (g/dL)		
Ethnicity		
**Coexisting Medical Conditions**		
MI (%)		
Stroke (%)		
TIA (%)		
Rheumatic heart disease (%)		
Peripheral arterial disease (%)		
Heart failure (%)		
NYHA class (%)<br>I-II<br>III<br>IV		
Thromboembolism (%)		
Diabetes mellitus (%)		
Aortic Plaque (%) (Y, N, Unknown)		
Smoking Status<br>Never<br>Current<br>Former		
Hypertension %)		
BMI (kg/m2)		
CHA_2DS_2VASc (mean)		
CHA_2DS_2VASc (category by 0 - 2; 3 and 4; ≥5)		
Rhythm on baseline ECG<br>AF<br>Aflutter<br>Sinus<br>Other		

LV Ejection Fraction (mean)		
LV Grade (%)<br>1<br>2<br>3<br>4		
**Preoperative Medications (within 7 days) (%)**		
ASA		
Other antiplatelet		
No OAC (Neither NOAC or VKA)		
Vitamin K Antagonist		
NOAC<br>Dabigatran<br>Rivaroxaban<br>Apixaban<br>Other		
Diuretic		
Anti-arrhythmic drug		
Digoxin		
ACE/ARB		
Beta-Blocker		
Statin		
Rate-controlling CCB		
Entresto		
**LAAOS III Recruitment Globally**		
Proportion patients recruited from:<br>i. North America (Canada, USA),<br>ii. Asia (China, India, Hong Kong, Malaysia, Japan)<br>iii. South America (Brazil, Argentina, Colombia),<br>iv. Europe (Austria, Belgium, Czech Republic, Ireland, Italy, Germany, Greece, Netherlands, Poland, Portugal, Russia, Spain, Switzerland, United Kingdom),<br>v. Africa and Middle East (Egypt, Iran),<br>vi. Oceania (Australia, New Zealand)		

Table 3. Operative Characteristics

Characteristic	Occlusion	No Occlusion
Bypass time (SD)		
Cross clamp time (SD)		
Procedure<br>i. Isolated CABG (%)<br>ii. Isolated valve (%)<br>iii. Any Valve Procedure (%)<br>Mitral<br>Aortic		

Tricuspid<br>Mitral<br>iv. Any aortic procedure (%)<br>v. Other (%)		
Atrial Ablation procedure (%)		
Pulmonary vein isolation		
Complete Left ablation		
Biatrial lesions		
PVI confirmed		
Energy used for ablation<br>Radiofrequency<br>Cryotherapy<br>Microwave<br>Laser<br>HIFU<br>Cut and sew		
Received treatment as assigned (%)		
Method of LAA occlusion (%) and success (%)		

Primary Outcome Tables

**Outcome**	**Occlusion**	**No Occlusion**	**HR (95% CI)**	**p-value**
Primary- ischemic stroke* or transient ischemic attack with positive neuroimaging or systemic arterial embolism				
Components of Composite<br>• ischemic stroke* or transient ischemic attack with positive neuroimaging<br>• systemic arterial embolism				

Secondary Outcomes Tables

**Binary Outcomes**				
**Outcomes**	**Occlusion**	**No Occlusion**	**HR (95% CI)**	**p-value**
All cause stroke or transient ischemic attack with positive neuroimaging or systemic arterial embolism				
All cause stroke or				

transient ischemic attack with positive neuroimaging or systemic arterial embolism or death				
Primary > 30 days after surgery				
All-cause death				
Stroke				
Hospitalization for heart failure				
Major bleed				
Myocardial infarction				
30-day Mortality				
Re-operation for bleeding			**RR**	
**Continuous Outcomes**				
**Outcomes**	**Occlusion**	**No Occlusion**	**Mean Difference (95% CI)**	**p-value**
Chest tube output (ml)				

SENSITIVITY ANALYSIS TABLES

Landmark Analyses (30 days)

Outcome	Occlusion	No Occlusion	HR (95% CI)	p-value
Primary- ischemic stroke* or transient ischemic attack with positive neuroimaging or systemic arterial embolism				
Hospitalization for heart failure				

Per Protocol Analyses

Outcome	Occlusion	No Occlusion	HR (95% CI)	p-value
Primary- ischemic stroke* or transient ischemic attack with positive neuroimaging or systemic arterial embolism				
Hospitalization for Heart Failure				

DocuSign Envelope ID: 58EBE868-868F-44D0-B13A-68108E4DA309

As Treated Analyses

Outcome	Occlusion	No Occlusion	HR (95% CI)	p-value
Primary- ischemic stroke* or transient ischemic attack with positive neuroimaging or systemic arterial embolism				
Hospitalization for Heart Failure				
