# Supplementary material for: Stroke Mechanism and Severity After Left Atrial Appendage Occlusion: Insights From the LAAOS III Randomized Clinical Trial
Source: JAMA Neurol. 2025 Nov 17;83(1):76–82. doi: 10.1001/jamaneurol.2025.4478 (PMC12624455; doi:10.1001/jamaneurol.2025.4478)
Supplement: Supplement 3. — eFigure 1. Algorithm Used for the Adjudication of Stroke Subtype eFigure 2. Cumulative Rates of First Stroke Due to Small Vessel Disease During Follow-Up e Figure 3. Cumulative Rates of First Stroke Due to Large-Artery Atherosclerosis During Follow-Up eFigure 4. Cumulative Rates of First Stroke Due to Other Defined Etiology During Follow-Up eFigure 5. Cumulative Rates of First Stroke Due to an Uncertain Etiology During Follow-Up eTable. Localization, Vascular Territory, and Subtype of First and Recurrent Ischemic Strokes in the Per-Protocol Population [file jamaneurol-e254478-s003.pdf]

Supplementary Online Content

Katsanos AH, Whitlock RP, Belley-Côté EP, et al. Stroke mechanism and severity after left atrial appendage occlusion: insights from the LAAOS III randomized clinical trial. *JAMA Neurol*. Published online November 17, 2025.
doi:10.1001/jamaneurol.2025.4478

eFigure 1. Algorithm Used for the Adjudication of Stroke Subtype

eFigure 2. Cumulative Rates of First Stroke Due to Small Vessel Disease During Follow-Up

e Figure 3. Cumulative Rates of First Stroke Due to Large-Artery Atherosclerosis During Follow-Up

eFigure 4. Cumulative Rates of First Stroke Due to Other Defined Etiology During Follow-Up

eFigure 5. Cumulative Rates of First Stroke Due to an Uncertain Etiology During Follow-Up

eTable. Localization, Vascular Territory, and Subtype of First and Recurrent Ischemic Strokes in the Per-Protocol Population

This supplementary material has been provided by the authors to give readers additional information about their work.

© 2025 Katsanos AH et al. *JAMA Neurology*.

eFigure 1. Algorithm used for the adjudication of stroke subtype

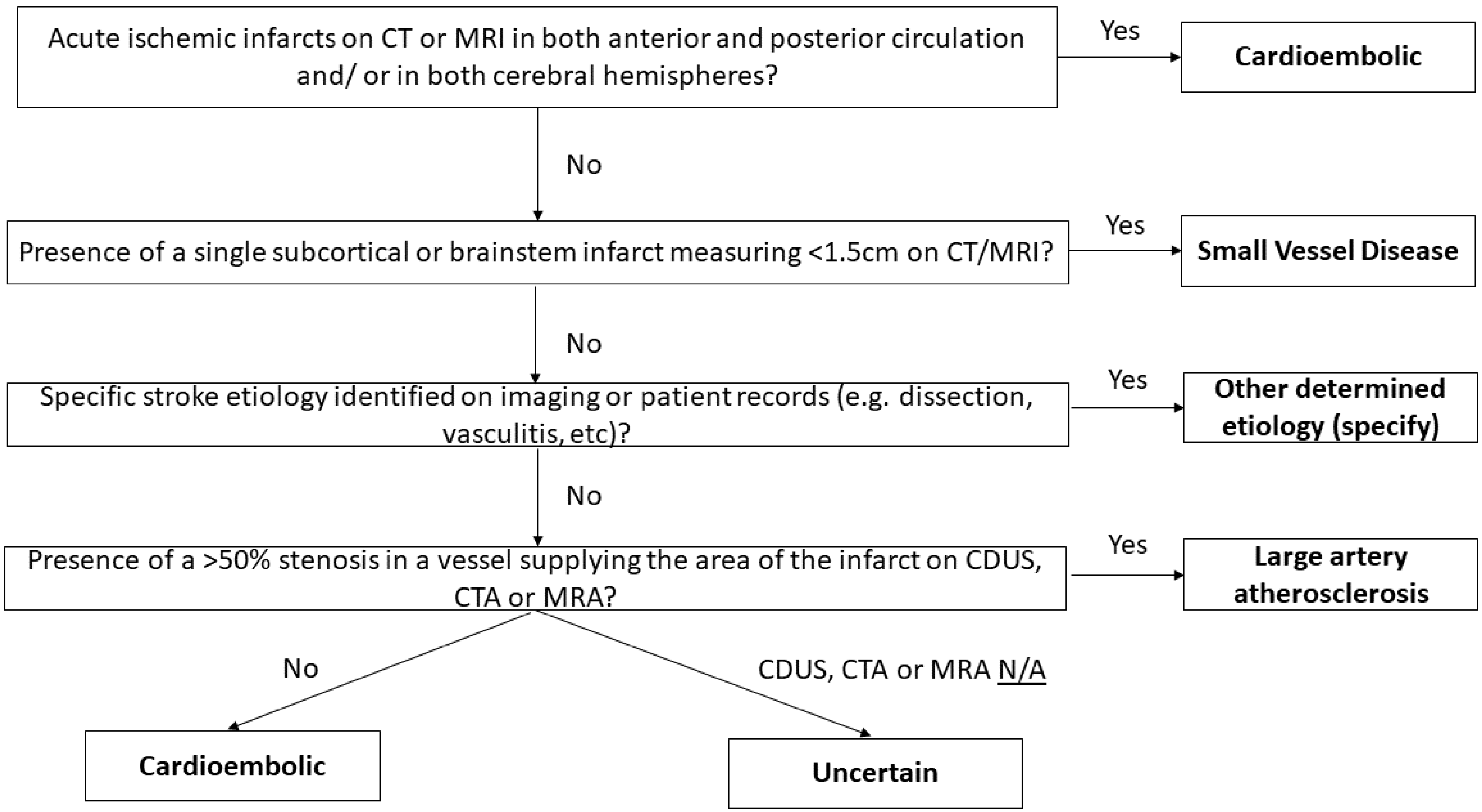

© 2025 Katsanos AH et al. *JAMA Neurology*.

eFigure 2. Cumulative rates of first stroke due to small vessel disease during follow-up.

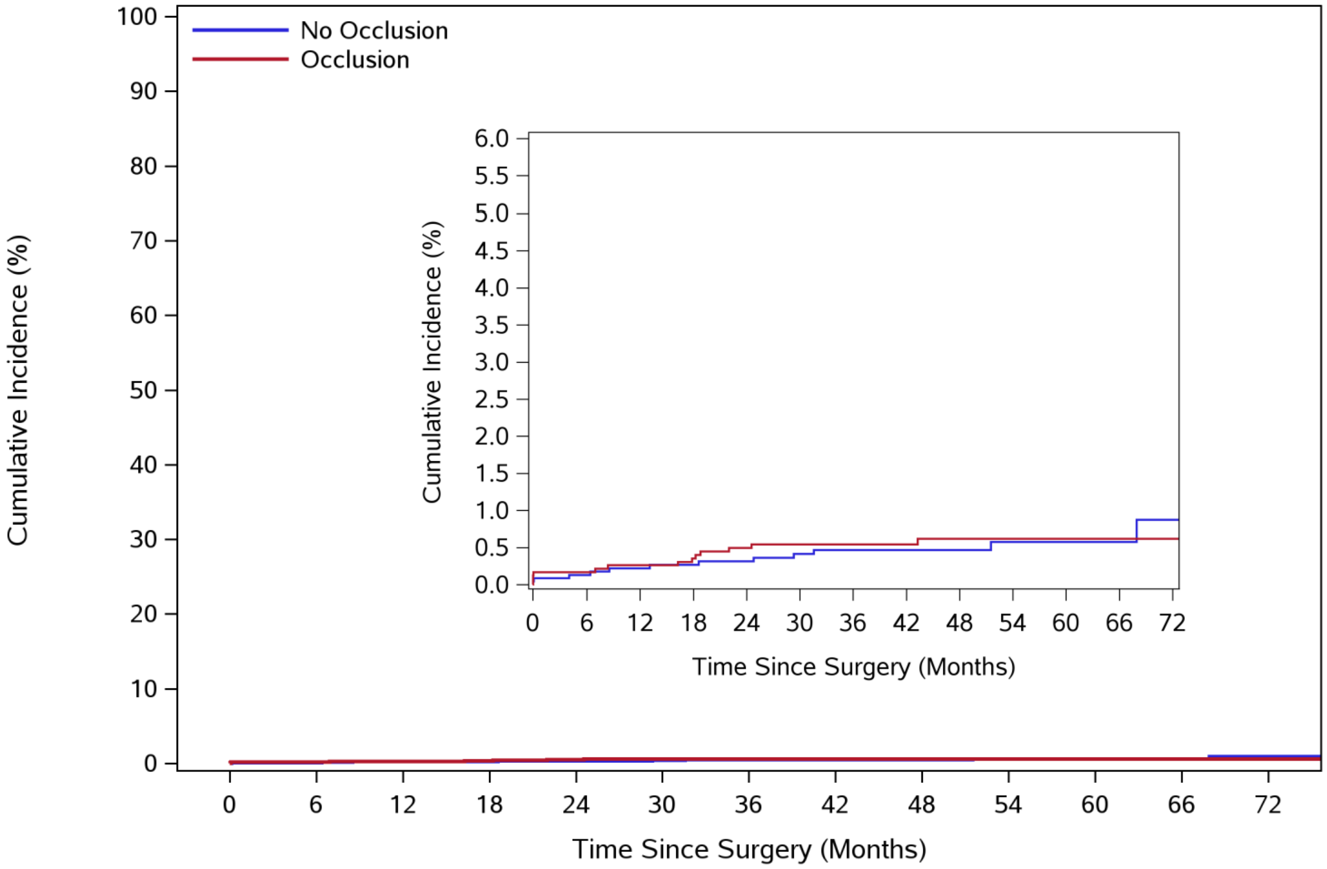

No. at Risk:

No Occlusion	2391	2198	2151	2106	2065	1982	1682	1380	1067	787	575	377	226
Occlusion	2379	2216	2162	2120	2077	2010	1700	1392	1081	807	566	358	205

© 2025 Katsanos AH et al. *JAMA Neurology*.

eFigure 3. Cumulative rates of first stroke due to large-artery atherosclerosis during follow-up.

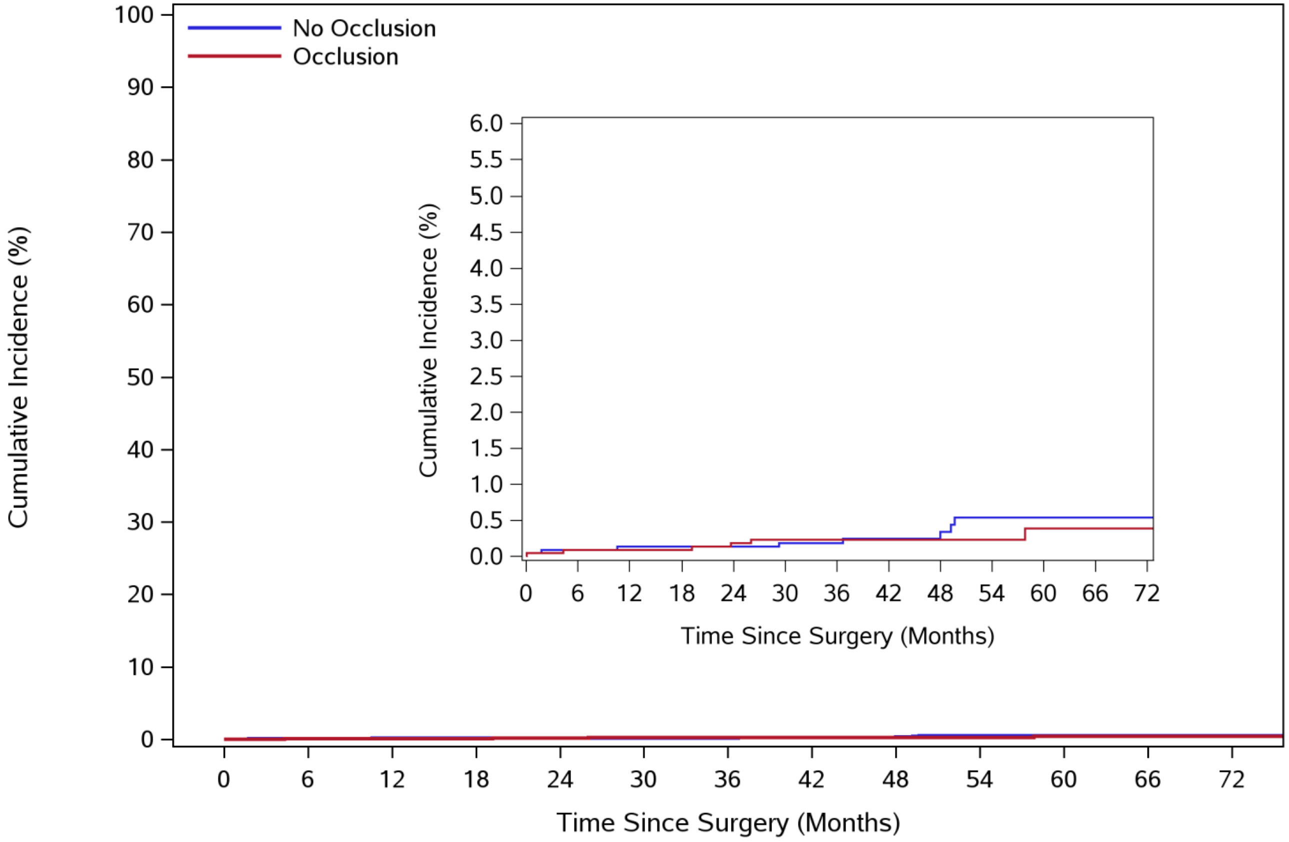

No. at Risk:

No Occlusion	2391	2200	2153	2109	2069	1988	1687	1382	1068	786	574	375	226
Occlusion	2379	2218	2165	2124	2081	2014	1704	1396	1084	808	566	358	205

© 2025 Katsanos AH et al. *JAMA Neurology*.

eFigure 4. Cumulative rates of first stroke due to other defined etiology during follow-up.

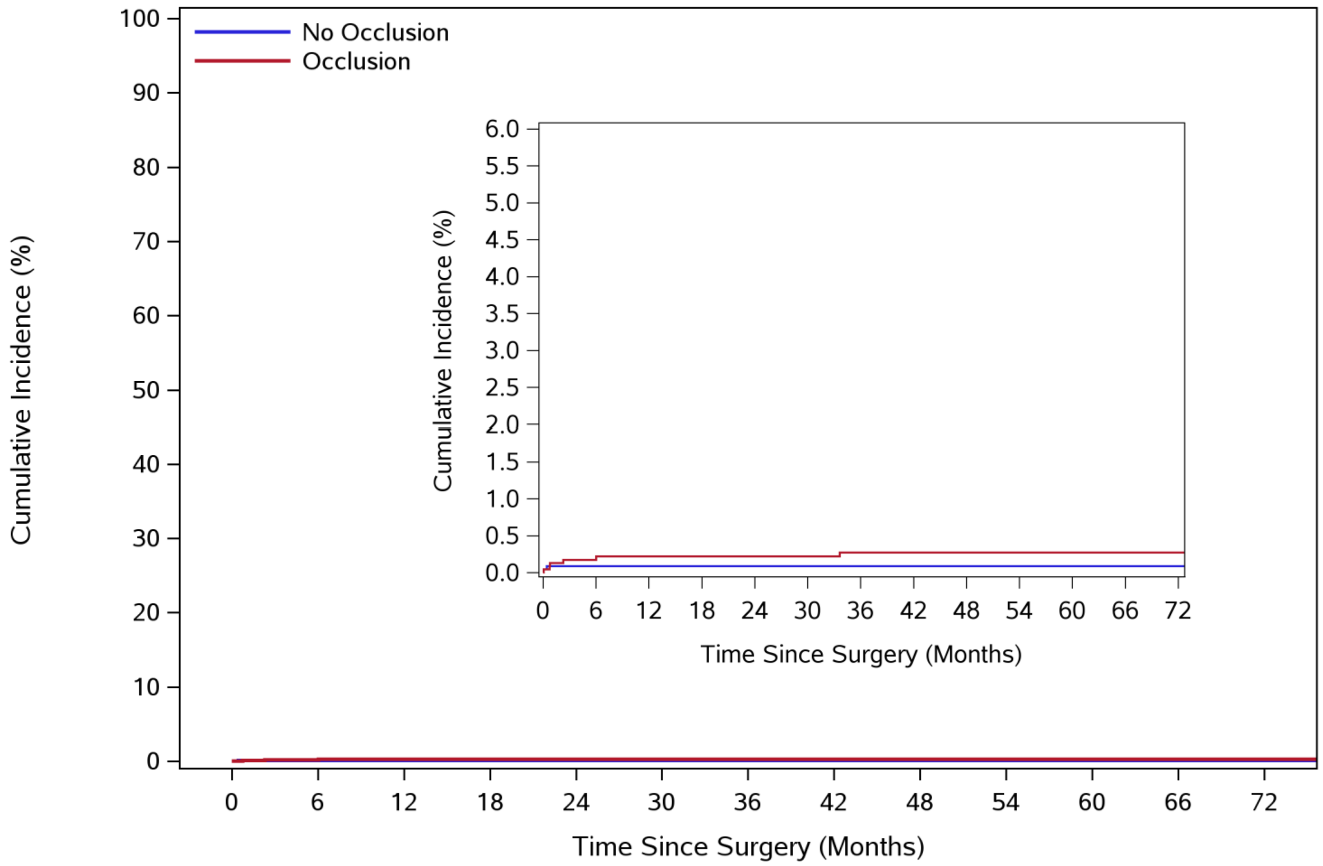

No. at Risk:

	0	6	12	18	24	30	36	42	48	54	60	66	72
No Occlusion	2391	2199	2154	2110	2070	1989	1689	1385	1070	790	577	378	227
Occlusion	2379	2217	2163	2122	2080	2014	1702	1395	1083	808	567	359	205

© 2025 Katsanos AH et al. *JAMA Neurology*.

eFigure 5. Cumulative rates of first stroke due to an uncertain etiology during follow-up.

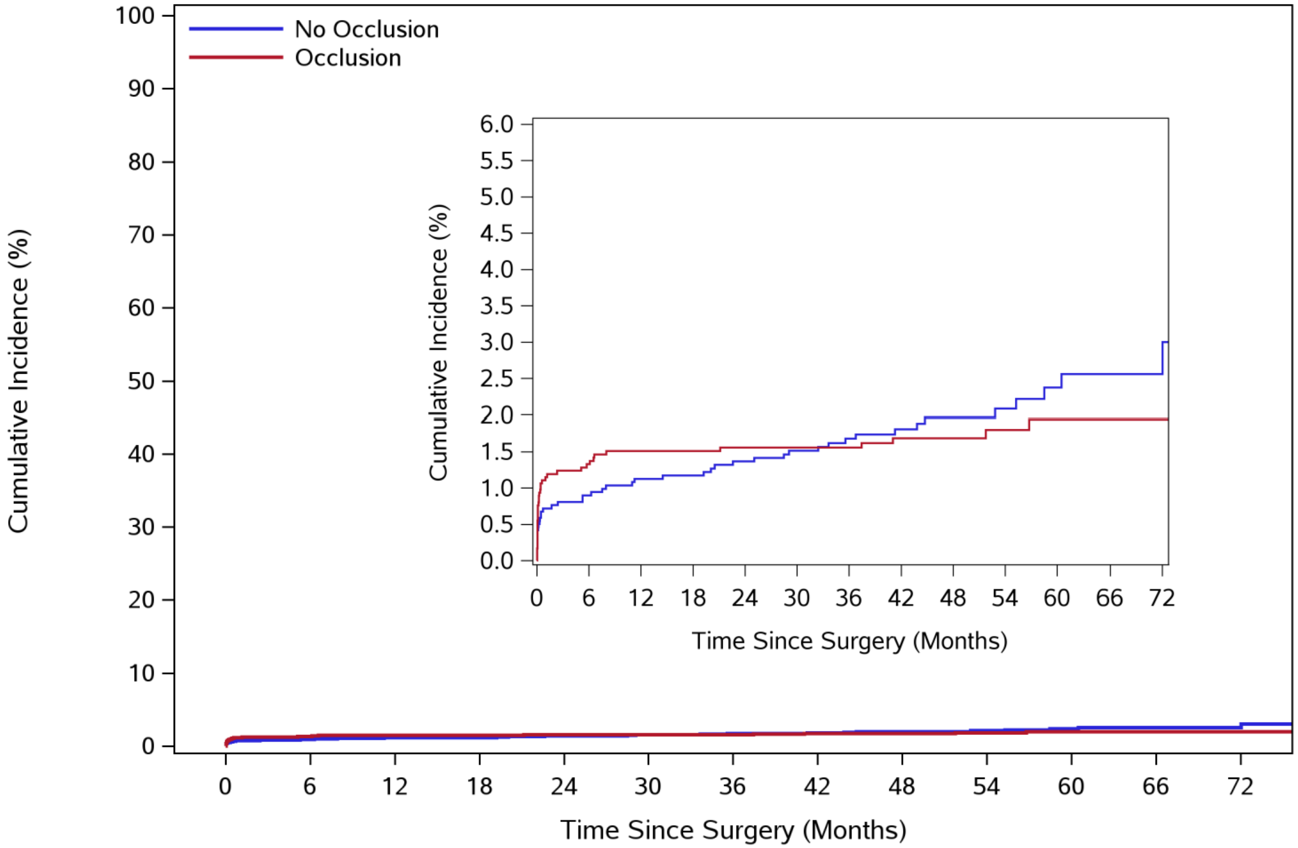

No. at Risk:													
No Occlusion	2391	2183	2133	2089	2047	1966	1667	1365	1054	781	568	369	221
Occlusion	2379	2191	2138	2097	2058	1992	1682	1374	1072	798	559	354	202

© 2025 Katsanos AH et al. *JAMA Neurology*.

eTable. Localization, vascular territory, and subtype of first and recurrent ischemic strokes in the per-protocol population.

	LAAO	No LAAO	Difference in Proportion (95% CI)	p-value
No. of Strokes	107	173		
**Localization**				
Cortical - no. (%)	50/106 (47.2%)	104/170 (61.2%)	-14.0% (-26.0%, -2.0%)	0.023
Subcortical - no. (%)	33/106 (31.1%)	36/170 (21.2%)	10.0% (-0.8%, 20.7%)	0.063
Uncertain - no. (%)	23/106 (21.7%)	30/170 (17.7%)	4.1% (-5.7%, 13.8%)	0.406
**Vascular territory**				
Multiple- no. (%)	26/106 (24.5%)	42/171 (24.6%)	-0.03% (-10.5%, 10.4%)	0.995
Single - no. (%)	65/106 (61.3%)	100/171 (58.5%)	2.8% (-9.0%, 14.7%)	0.640
Uncertain - no. (%)	15/106 (14.2%)	29/171 (17.0%)	-2.8% (-11.5%, 5.9%)	0.534
**Subtype***				
Cardioembolism - no. (%)	48/107 (44.9%)	101/172 (58.7%)	-13.9% (-25.8%, -1.9%)	0.024
Small vessel disease - no. (%)	10/107 (9.4%)	12/172 (7.0%)	2.4% (-4.3%, 9.1%)	0.475
Large artery atherosclerosis - no. (%)	7/107 (6.5%)	10/172 (5.8%)	0.7% (-5.1%, 6.6%)	0.805
Other defined etiology - no. (%)	5/107 (4.7%)	2/172 (1.2%)	3.5% (-0.8%, 7.8%)	0.111
Uncertain - no. (%)	37/107 (34.6%)	47/172 (27.3%)	7.3% (-4.0%, 18.5%)	0.199

*based on modified TOAST (Trial of Org 10172 in Acute Stroke Treatment) criteria

© 2025 Katsanos AH et al. *JAMA Neurology*.
