# Supplementary material for: Stroke Mechanism and Severity After Left Atrial Appendage Occlusion: Insights From the LAAOS III Randomized Clinical Trial
Source: JAMA Neurol. 2025 Nov 17;83(1):76–82. doi: 10.1001/jamaneurol.2025.4478 (PMC12624455; doi:10.1001/jamaneurol.2025.4478)
Supplement: Supplement 4. — Data Sharing Statement. [file jamaneurol-e254478-s004.pdf]

Data Sharing Statement

Katsanos. Stroke Mechanism and Severity After Left Atrial Appendage Occlusion. *JAMA Neurol*. Published November 17, 2025. doi:10.1001/jamaneurol.2025.4478

Data
Additional Information: ClinicalTrials.gov number, NCT01561651
Data available: No
